# Long-Term Improvement in Hippocampal-Dependent Learning Ability in Healthy, Aged Individuals Following High Intensity Interval Training

**DOI:** 10.14336/AD.2024.0642

**Published:** 2025-06-27

**Authors:** Daniel G. Blackmore, Mia A. Schaumberg, Maryam Ziaei, Samuel Belford, Xuan Vinh To, Imogen O’Keeffe, Anne Bernard, Jules Mitchell, Emily Hume, Grace L. Rose, Thomas Shaw, Ashley York, Markus Barth, Elizabeth J. Cooper, Tina L. Skinner, Fatima Nasrallah, Stephan Riek, Perry F. Bartlett

**Affiliations:** ^1^Queensland Brain Institute, The University of Queensland, Brisbane, Australia.; ^2^Clem Jones Centre for Ageing Dementia Research, Queensland Brain Institute, The University of Queensland, Brisbane, Australia.; ^3^School of Human Movement and Nutrition Science, The University of Queensland, Brisbane, Australia.; ^4^School of Health, University of the Sunshine Coast, Sippy Downs, Australia.; ^5^Sunshine Coast Health Institute, Birtinya, Queensland, Australia.; ^6^The Centre for Advanced Imaging, The University of Queensland, Brisbane, Australia.; ^7^Queensland Cyber Infrastructure Foundation, The University of Queensland, Brisbane, Australia.; ^8^School of Electrical Engineering and Computer Science, The University of Queensland, Brisbane, Australia.; ^9^Graduate Research School, University of the Sunshine Coast, Sippy Downs, Australia.

**Keywords:** exercise, hippocampal-dependent, memory, biomarkers

## Abstract

Physical exercise may reduce dementia risk in aging, but varying reports on its effectiveness make it challenging to ascribe what level of exercise will have significant longer-term effects on important functions such as hippocampal-based learning and memory. This study compared the effect of three different 6-month exercise regimens on hippocampal-dependent cognition in healthy, elderly individuals. Participants, aged 65-85 with no cognitive deficits, were randomly assigned to one of three exercise interventions (low (LIT), medium (MIT), and High intensity interval training (HIIT), respectively). Each participant attended 72 supervised exercise sessions over a 6-month period. A total of 151 participants completed all sessions. Cognitive testing for hippocampal performance occurred monthly, as did blood collection, and continued for up to 5 years following initiation of the study. Multimodal 7 Tesla MRI scans were taken at commencement, 6 and 12 months. After 6 months, only the HIIT group displayed significant improvement in hippocampal function, as measured by paired associative learning (PAL). MRI from the HIIT group showed abrogation of the age-dependent volumetric decrease within several cortical regions including the hippocampus and improved functional connectivity between multiple neural networks not seen in the other groups. HIIT-mediated changes in the circulating levels of brain-derived neurotrophic factor (BDNF) and cortisol correlated to improved hippocampal-dependent cognitive ability. These findings demonstrate that HIIT significantly improves and prolongs the hippocampal-dependent cognitive health of aged individuals. Importantly, improvement was retained for at least 5 years following initiation of HIIT, suggesting that the changes seen in hippocampal volume and connectivity underpin this long-term maintenance. Sustained improvement in hippocampal function to this extent confirms that such exercise-based interventions can provide significant protection against hippocampal cognitive decline in the aged population. The changes in specific blood factor levels also may provide useful biomarkers for choosing the optimal exercise regimen to promote cognitive improvement.

## INTRODUCTION

Aging dementia, including Alzheimer's disease (AD), is a significant health concern, with more than 130 million people worldwide predicted to suffer from the condition by 2050 [1]. Delaying the onset of dementia by five years would result in a decreased prevalence of 41% by 2050 [2]. Therefore, it is critical to identify approaches that delay, slow or even reverse age-associated cognitive decline. Modifiable lifestyle factors such as physical activity [3] have been proposed to be effective at altering the trajectory of aging dementia.

A key feature of aging dementia is the decline in specific domains of cognitive function, especially those related to spatial learning and memory. The hippocampus is a critical region of the brain that is responsible for the consolidation of spatial information into memories and is particularly susceptible to age [4], with reports of age-dependent decreased hippocampal volume [5] and connectivity [6]. Physical exercise is a promising non-invasive approach to ameliorate age-associated hippocampal cognitive deficits. Rodent studies have clearly demonstrated that physical exercise improves hippocampal-dependent spatial learning [7, 8], through several key exercise-mediated mechanisms including increased neural stem cell (NSC) activation [9, 10], hippocampal neurogenesis [11], and improved hippocampal connectivity [12]. Some of these processes, including NSC activation and neurogenesis, have also been linked to specific circulating biomarkers [8, 13], and the duration of exercise has been shown to be critical for these effects [8, 12].

Despite the work conducted in adult and aged animal models demonstrating exercise-mediated improvement in hippocampal-dependent cognition, there is a lack of studies examining the extent to which physical exercise affects hippocampal function in aged humans. Indeed, there is only one study that expressly examined hippocampal-dependent spatial learning in the healthy elderly following MIT exercise [14]. While the authors found a marked increase in hippocampal volume with 12 months of MIT exercise, no improvement in hippocampal-dependent spatial memory above that of the control group was observed [14]. The same study also measured the change in concentration of BDNF but found no correlation to hippocampal cognitive ability. However, most studies that have examined exercise-mediated changes in circulating analytes and cognitive function have been limited to one factor [15] or, at most, a few analytes, as recently reviewed [16]. Such an approach limits the ability to accurately identify circulating biomarkers that may be involved in exercise-mediated changes in cognitive ability. Further, in a recent umbrella review of meta-analyses examining the link between physical exercise and various domains of cognition in healthy populations, only small effect sizes were found, cautioning against claims of exercise-mediated effects on cognition [17]. A lack of accounting for key moderators such as active controls and baseline differences between groups were identified as major limitations when reporting the effect of exercise on cognition [17]. These, coupled with a heterogenous population, a propensity to rely on self-reported measures including exercise intensity [14, 18], and the duration of exercise intervention ranging from weeks [19] to years [20], make the large variation in reported outcomes unsurprising. There is evidence that the age-associated volumetric atrophy in the hippocampus [21] and decrease in key functional networks [22] such as the default mode network (DMN), can be ameliorated following exercise [23, 24]. However, as with cognitive studies, the amount of exercise required to achieve optimal outcomes in structural and functional connectivity remains unclear.

Therefore, to address these shortcomings, we conducted a randomized control study to investigate the effect of exercise on the healthy elderly. Our primary objective was to measure changes in cognitive function following exercise. This was examined using a longitudinal approach where we took advantage of electronic cognitive testing as described below. The secondary outcomes we also examined included: 1) if exercise could improve hippocampal-dependent spatial learning in healthy, aged individuals, 2) if there was a dose-dependent component, 3) if hippocampal cognitive improvements could be predicted by longitudinal changes in biochemical and/or physiological parameters and 4) if any exercise-mediated long-term improvement in hippocampal learning was observed. To do this, we performed a multimodal, randomized 6-month exercise intervention with increasing doses of exercise comprising an active control group (low intensity training- LIT), a MIT group and a high intensity interval training (HIIT) group. Participants exercised three times a week under constant supervision by qualified exercise physiologists to ensure that personalized target heart rates (HR) were reached and maintained during the exercise sessions. Numerous parameters were recorded monthly to examine each participant before, during and following the exercise intervention. These included cognitive function, as assessed electronically through multiple CANTAB® tests, including the hippocampal-dependent paired associated learning (PAL) test. Other cognitive domains including working memory, visual working memory, and emotional recognition were also examined. Monthly blood samples were also collected immediately before and after exercise to examine changes in several circulating biochemical markers. Training diaries recorded HR, and treadmill parameters for MIT and HIIT participants multiple times each session. Multimodal, high resolution brain scans using ultra-high field magnetic resonance imaging (MRI) were also obtained for a subset of participants at the initiation and at completion of the intervention, as well as 6 months later.

We found that:

- Hippocampal-dependent cognition improved significantly in the HIIT group after 6 months of exercise and that the improvement was maintained for up to 5 years from the initiation of the exercise intervention.
- In the LIT and MIT groups, hippocampal-dependent cognition was maintained during the exercise intervention and showed no age-related decline compared with baseline performance at the later time points.
- HIIT abrogated the age-associated decreases in brain volume observed in the LIT and MIT groups.
- Significant increases in functional connectivity occurred between multiple key resting-state network circuits following 6 months of HIIT.
- An increase in initial exercise-mediated cortisol and BDNF levels predicted cognitive improvement in HIIT participants.

## METHODS

### Study design and participants

We conducted a multidomain, randomized controlled study over a period of 6 years at a single site located in a purpose-built clinic at The University of Queensland (UQ). The participants who were recruited ranged from 65-85 years of age. Detailed recruitment, screening, inclusion, exclusion, randomization, and withdrawal of participants are described in S1 Appendix. Inclusion criteria included the ability to provide written, informed consent to participant in the study, be between 65-85 years of age, have to ability to communicate in English, no history of stroke, heart or brain surgery or brain trauma, stratified as “not high risk of experiencing a cardiac event during exercise’ according to pre-exercise screening, no presently diagnosed mental illness or cognitive impairment, as per baseline Addenbrooke’s cognitive examination-revised (ACE-R) scores, not be medicated for dementia or psychiatric illnesses, have a healthy body mass index; and be able and willing to commit to the duration of the exercise program.

Exclusion criteria included 1) being younger than 65 or older than 85 years of age; 2) illness or disability that precluded exercise or hindered completion of the study; 3) poorly controlled hypertension, cardiomyopathy, unstable angina, heart failure or severe arrhythmia, cancer, or chronic communicable infectious diseases; 4) current use of antipsychotics and/or antidepressants; 5) current or planned use of dehydroepiandrosterone, testosterone, transdermal oestrogens or other medications known to affect the growth hormone releasing hormone/growth hormone/insulin-like growth factor-1 axis; 6) currently diagnosed significant psychiatric illness, such as depression or schizophrenia, or dementia; 7) any contraindication for 7 Tesla MRI investigation, including recent joint replacements, stents, brain or heart surgery, bullet or shrapnel wounds and metal in the eye; 8) tobacco use, excessive alcohol intake (more than four alcoholic drinks per day), excessive caffeine intake (more than four cups of coffee per day); 9) excessive exercise involvement (more than twice the weekly recommendations for adults or >600 min moderate to vigorous exercise per week); 10) test results that indicated study participation was unsafe or; 11) participation in conflicting studies.

**Table 1:** Demographic and descriptive baseline data.

Exercise group	LIT (n=53)	MIT (n=44)	HIIT (n=54)	p value
**Age**	72.0 (±0.57)	71.9 (±0.63)	71.7 (±0.59)	n.s.
**Gender**	30F:24M	24F:20M	27F:27M	n.s.
**Education (y)**	15.3 (±0.54)	15.3 (±0.48)	15.7 (±0.56)	n.s.
**Weight (kg)**	73.22 (±1.62)	71.72 (±2.03)	73.97 (±1.77)	n.s.
**Height (cm)**	167.9 (±1.22)	166.2 (±1.20)	169.0 (±1.16)	n.s.
**BMI**	25.9 (±0.54)	25.9 (±0.87)	25.8 (±0.49)	n.s.
**WHR**	0.82 (±0.02)	0.86 (±0.02)	0.87 (±0.01)	n.s.
**DASS**	9.14 (±1.32)	8.39 (±1.10)	7.76 (±1.07)	n.s.
**INS**	5.44 (±0.59)	5.07 (±0.66)	4.31 (±0.59)	n.s.
**Resting HR**	68.5 (±1.30)	67.2 (±1.30)	69.0 (±1.49)	n.s.
**Resting BP**	128/70 (±1.7/1.2)	125/69 (±2.0/1.4)	130/72 (±2.1/1.3)	n.s.
**Grip strength**	31.29 (±1.3)	32.38 (±1.5)	32.94 (±1.4)	n.s.
**MMSE**	28.67 (±0.16)	28.91 (±0.19)	28.81 (±0.16)	n.s.
**ACE-R**	92.22 (±0.55)	94.04 (±0.68)	93.33 (±0.56)	n.s.

Comparison of basic demographic and descriptive data prior to the initiation of the exercise intervention. All groups were evenly distributed and showed no significant differences. All data represent a group mean (±SE). Statistical comparisons were conducted using one-way ANOVA with Bonferroni post-hoc tests.

Randomization of participants*:* We randomly assigned participants to one of three arms using a centrally controlled computer-generated randomization list, generated by an independent staff member. The treatment was assigned using sealed envelopes based on order of recruitment and stratified for sex only. All investigators were blinded to the allocation order; however, due to practicality, outcome assessors and exercise trainers were not blinded to the allocation treatment arm.

**Figure 1.**
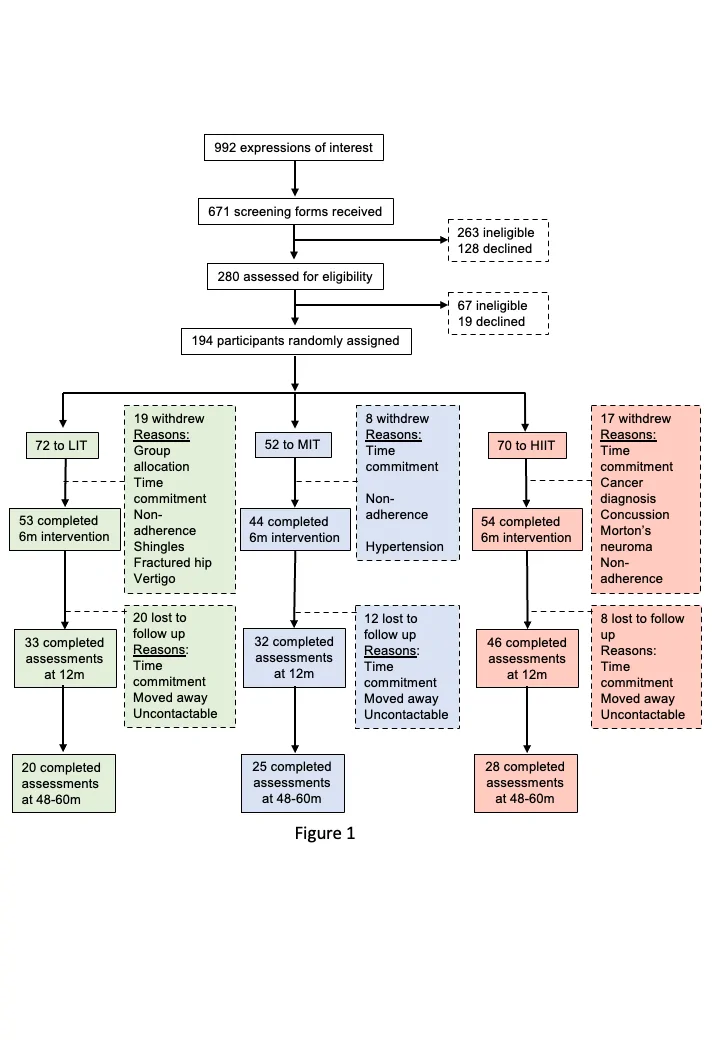
**Cohort schematic illustrating recruitment and participation for each group during the 6-month exercise trial and beyond**. The number of participants who were screened and assigned to each group is provided. The reasons for withdrawal are summarized.

After screening, 194 participants were recruited into the study, randomised into one of three groups in a 1:1:1 ratio with 151 completing the 6-month exercise intervention (see Fig. 1 for cohort diagram). Baseline demographic and descriptive data compared between all three exercise groups showed no significant differences (Table 1).

### Procedures

Participants completed three, 36-45 min exercise sessions per week over a 6-month period for a total of 72 exercise sessions; all training sessions were supervised by qualified exercise physiologists and were conducted in a small group setting. Exercise intensity was individually prescribed and regularly monitored via HR. Visual HR cues were provided on an individualized poster and HR was recorded at regular intervals during each session in a digital training diary. Peak aerobic capacity (VO_2peak_) and peak HR (HR_peak_) were determined via a maximal graded exercise test to volitional fatigue using automated indirect calorimetry and 12-lead ECG cardiac monitoring (COSMED). This was conducted at baseline and at the 3- and 6-month time points and served as the basis for the calculation of HR intensity zones for each participant during each exercise session.

All groups completed a 10 min warm up, followed by an exercise session at their appointed intensity with a 5 min cool-down period at the end of each session. The LIT control group conducted 30 min exercise sessions that included 5-8 stretching, balance, range of motion and relaxation tasks where HR was maintained at 45-55% HR_peak_ to ensure a negligible cardiometabolic effect. The MIT exercise sessions comprised of continuous treadmill walking at 60-75% HR_peak_ that lasted 30 min. Participants were required to reach their target HR at the end of the warm-up and were instructed to maintain the HR for the duration of the session. Each exercise session for the HIIT group totalled 25 min and comprised of four, 4 min working periods at 85-95% of HR_peak_ interspersed by three, 3 min active recovery intervals at 60-70% HR_peak_. Participants were required to reach their target HR within the first 2 min of the interval and maintain this HR until the end of the 4 min interval. In the MIT and HIIT groups, participants were free to alter the treadmill speed and/or treadmill incline to maintain the required HR (see S2 Appendix for detailed description for physiological associated methods and safety parameters). The total period for each session was therefore: LIT- 45 min, MIT - 45 min and HIIT- 40min.

Cognitive testing was conducted electronically each month during the exercise intervention and then every 6 months during the follow-up period which extended up to 60 months. Testing was carried out using the Cambridge Automated Neuropsychological Test Battery (CANTAB; Cambridge Cognition Ltd., 2015). These tests were self-administered using an iPad touch screen interface while in the clinic. Outcomes were automatically recorded in the application for later retrieval (see S3 Appendix).

MRI was conducted on a subset of participants prior to exercise (0 months), immediately following completion of the exercise intervention (6 months) and 6 months after completion of the exercise intervention (12 months). Scans were conducted at the Centre for Advanced Imaging at UQ using an ultra-high field 7T MRI, Siemens scanner (see S4 Appendix for detailed description of MRI-associated methods).

Blood biochemical properties were obtained from monthly blood samples that were collected immediately pre- and post-exercise. ELISAs were conducted as per the manufacturer’s recommendations for a total of 17 analytes (see S5 Appendix for detailed description of biochemical associated methods).

Physiological and functional fitness measurements were collected during in-person visits. At-home questionnaires were also completed by participants during the intervention and beyond (see S6 Appendix). Habitual physical activity was measured for 7 consecutive days using tri-axial accelerometry (Actigraph^®^) at 0, 3, 6, 12 and 42 months. This was done in the absence of the prescribed exercise sessions.

### Safety

An event was defined as any untoward medical occurrence in a participant undergoing an intervention. The reporting period for an adverse event was defined as the period from initiation of the study treatment to the end of the 12-month follow-up. All adverse events were recorded using authorized incident report forms and were reviewed by Occupational Health and Safety staff, project staff and the heads of organizational units. The principal investigator registered all adverse events with the ethical review committee within 24 h of the incident. No adverse events resulted in hospitalization. During the study there were a total of 2 adverse events. One participant from the LIT group fell into the moderate adverse event range (cardiac discomfort that occurred at home). One participant from the HIIT group fell into the severe adverse event range (injured knee requiring physiotherapy).

### Data storage, access and sharing

In accordance with the Australian Privacy Principles 2014, the Australian Code of the Responsible Conduct of Research and NHMRC National Guidelines for the Ethical Conduct of Human Research (2007), data were de-identified by referencing through a six-character participant code. DICOM images from the brain imaging sessions were anonymized by only using the participant code. Digital data including DICOM images and scanned paperwork in pdf format were stored on an access-controlled dedicated shared network drive with high integrity permanent backup capability, with access limited to authorized research and IT staff (see S7 Appendix for detailed description of data storage and access associated methods).

### Primary and secondary outcomes

The primary outcome - changes in cognitive function using CANTAB following exercise - was assessed at the completion of the 6-month exercise study using the detailed methodology provided in Appendix 3. The secondary outcomes examined in this manuscript include changes in blood biomarkers at month intervals for the duration of the intervention, changes in hippocampal volume, and changes in resting state functional activity. These outcomes were also assessed at the completion of the exercise intervention study and were measured using the methods described above and in the relevant supplementary appendices.

### Statistical analysis

Statistical description was provided as means and standard deviation (SD), mean and standard error (SEM) or median and inter-quartile range (IQR) for continuous data and frequency and percentage for categorical data as appropriate. Normality of the distribution of continuous variables was tested using the Shapiro-Wilk test with an alpha level <0·05. Data were log-transformed when appropriate and re-checked for normality of distribution. Associations between continuous variables was assessed using Pearson’s correlation coefficient for data normally distributed or Spearman’s correlation coefficient when normality assumption was not met. The comparison in continuous outcomes between groups at baseline was performed using one-way analysis of variance (ANOVA) where appropriate. Analysis of continuous outcomes over time and between groups was performed using either two-way repeated measures (RM) ANOVA or linear mixed-effects models (LMM) considering time, group and group by time as main effect and participants as random effect. Post-hoc tests with Bonferroni adjustments were performed where appropriate. Potential confounders affect such as age, gender, BMI were considered and tested in the model as fixed effects but were not found significant and therefore not included in the final models presented. Variables showing a significant difference at baseline were included in the regression analyses for the cognitive function outcome. Cohen’s d= “(µ1-µ2)/SD pooled” was used to calculate effect sizes where appropriate. Comparison of the functional connectivity between groups was assessed and reported using q-values, using an optimised false discovery rate (FDR) approach. Analyses were performed using the R statistical software (version 4.2), SPSS (version 22.0, SPSS, Inc.) and GraphPad Prism (version 10.0.3). P-values were two-tailed, with *p*< 0.05 considered statistically significant.

### Ethics and Trial Registration

The ethics approval number was 2016-01-038-A-5 (Bellberry Ltd- https://bellberry.com.au). The Universal Trial Number (UTN) is: U1111-1185-5102. The trial application approval number from ANZCTR is ACTRN12618000700235.

## RESULTS

### HIIT exercise significantly improves hippocampal-dependent spatial learning following 6 months of exercise

**Figure 2.**
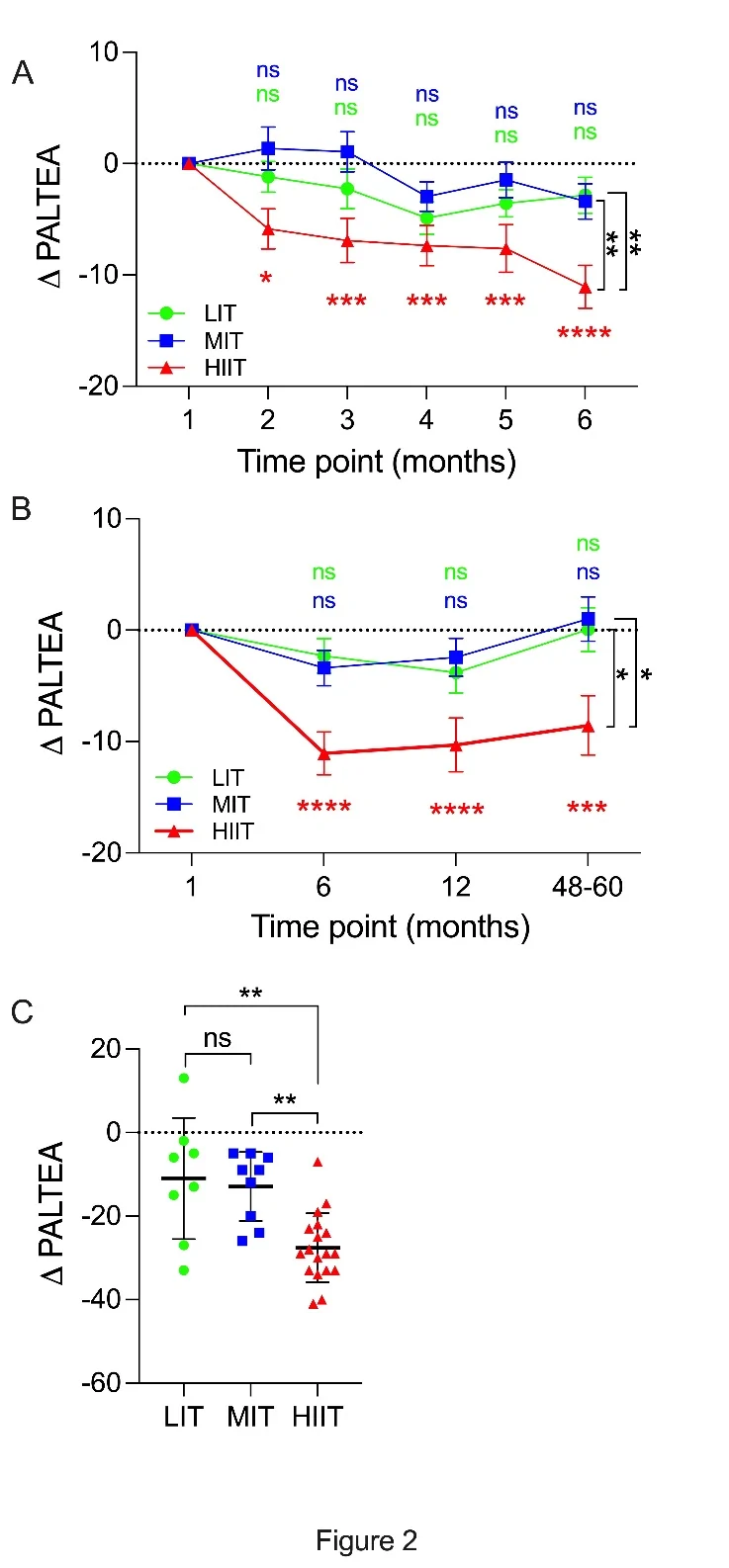
**Learning performance was significantly improved during the exercise intervention period and beyond**. (**A**) Only the HIIT group exhibited significantly improved PALTEA performance (fewer errors) during the exercise intervention. Neither the LIT nor the MIT group exhibited altered PALTEA performance during the exercise intervention. At the 6-month timepoint the HIIT group significantly outperformed the LIT group (mean±SE; two-way RM-ANOVA [time x exercise intensity effect F (10,738) = 2.272, *p*= 0.012], with Bonferroni post-hoc tests, *p*= 0.004 and *p*= 0.008 respectively, effect sizes LIT: HIIT = 0.72, MIT: HIIT = 0.74). (**B**) The improvement in PALTEA performance for the HIIT group observed following 6 months of exercise was maintained for up to 5 years following exercise initiation. Conversely, both the LIT and MIT groups remained stable at each time point measured (mean±SE; two-way RM-ANOVA [time x exercise intensity effect F (6,317) = 4.076, *p*= 0.0006], repeated measures with Bonferroni’s post-hoc tests, *p*= 0.036 and *p*= 0.017 respectively, 12m effect sizes; LIT: HIIT = 0.5, MIT: HIIT effect size = 0.62. >48m effect sizes; LIT: HIIT = 0.73, MIT: HIIT = 0.77). (**C**) Comparing cognitive performance for participants who were +1SD above mean PALTEA performance at the start of testing showed that the HIIT group significantly improved and outperformed both the LIT and MIT groups (mean±SD; one-way ANOVA F (2,32) = 10.71, *p*= 0.0003, with Bonferroni’s post hoc tests, effect sizes: LT: HIIT= 1.4, MIT: HIIT= 1.7). Within group statistical comparisons are represented by significance indicators of the same colour whereas black significance indicators represent differences between groups. ns = non-significant, *p< 0.05, **p< 0.01, ***p< 0.001, ****p< 0.0001.

Longitudinal changes in PALTEA performance during the intervention period showed that only the HIIT group demonstrated a significant improvement in ∆PALTEA (decreased errors) during each month of the exercise intervention, with the largest improvement being reached after 6 months of exercise. In contrast, both the LIT and MIT groups remained stable at each time point during the 6-month exercise intervention (Fig. 2A). By the completion of the exercise intervention, the PALTEA performance for the HIIT group was significantly better than that of both the LIT and MIT groups (*p*= 0.004 and *p*= 0.008 respectively).

Prior to, and during the intervention, participants underwent a comprehensive battery of physiological tests. We found no significant differences either between groups or within groups for physiological measures such as weight, BMI, grip strength or blood pressure at either baseline (Table 1) or immediately following 6 months of exercise (Supp. Table 1). A graded balance test was also performed with both the LIT and HIIT group demonstrating a significant increase in balance at the conclusion of the exercise intervention (Supplementary Fig. 1A). Cardiorespiratory tests were also conducted immediately prior to, at the 3-month, 6-month and 12-month periods (Supplementary Fig. 1B). There was a main effect of exercise intensity during the testing period when we examined V0_2_/kg (2-way RM-ANOVA, exercise intensity: F (2,149) = 4.675, *p* =0.011) with post hoc analysis showing a significant difference between LIT and HIIT groups at the 3-month test period. While both MIT and HIIT groups showed an increase in cardiorespiratory fitness during the exercise intervention period, this did not reach significance. Further, comparing the change in V0_2_/kg to ∆PALTEA showed no correlation between these two metrics.

### Hippocampal-dependent cognitive improvements are maintained for up to 5 years following initiation of the HIIT intervention

Participants were re-tested 6 months after the completion of the exercise intervention (the 12-month timepoint) and then regularly for the following 4 years. The results showed that the HIIT group maintained the PALTEA improvement (decreased errors) not only at the 12-month time point but continued to do so for up to 5 years following the initiation of the exercise trial (Fig. 2B). PALTEA performance for the LIT and MIT groups was not significantly different from baseline (Fig. 2B). At each testing period following the conclusion of the exercise trial, the HIIT group performed significantly better than both the LIT and MIT groups. These improvements were not due to differences in lifestyle or physical activity following the 6-months exercise period as measured by both the Godin Leisure Time Physical Activity Questionnaire and accelerometry data (Supplementary Fig. 1). Rather, the increase in, and retention of improved cognitive function in the HIIT group appeared to be a direct result of the 6-month HIIT exercise intervention.

### Participants who initially perform poorly on PALTEA show marked improvement with HIIT

As there was a large variation in PALTEA performance at screening, we examined whether the group with the highest errors (poor performers), which were +1SD above mean at screening, responded to the HIIT. We found that this group showed the largest increase in performance which again was maintained for 5 years. Interestingly, the poor performers in the MIT group also showed some improvement, although the magnitude of the effect was much smaller than that seen with the HIIT group (Fig. 2C, and Supplementary Fig. 2).

**Figure 3.**
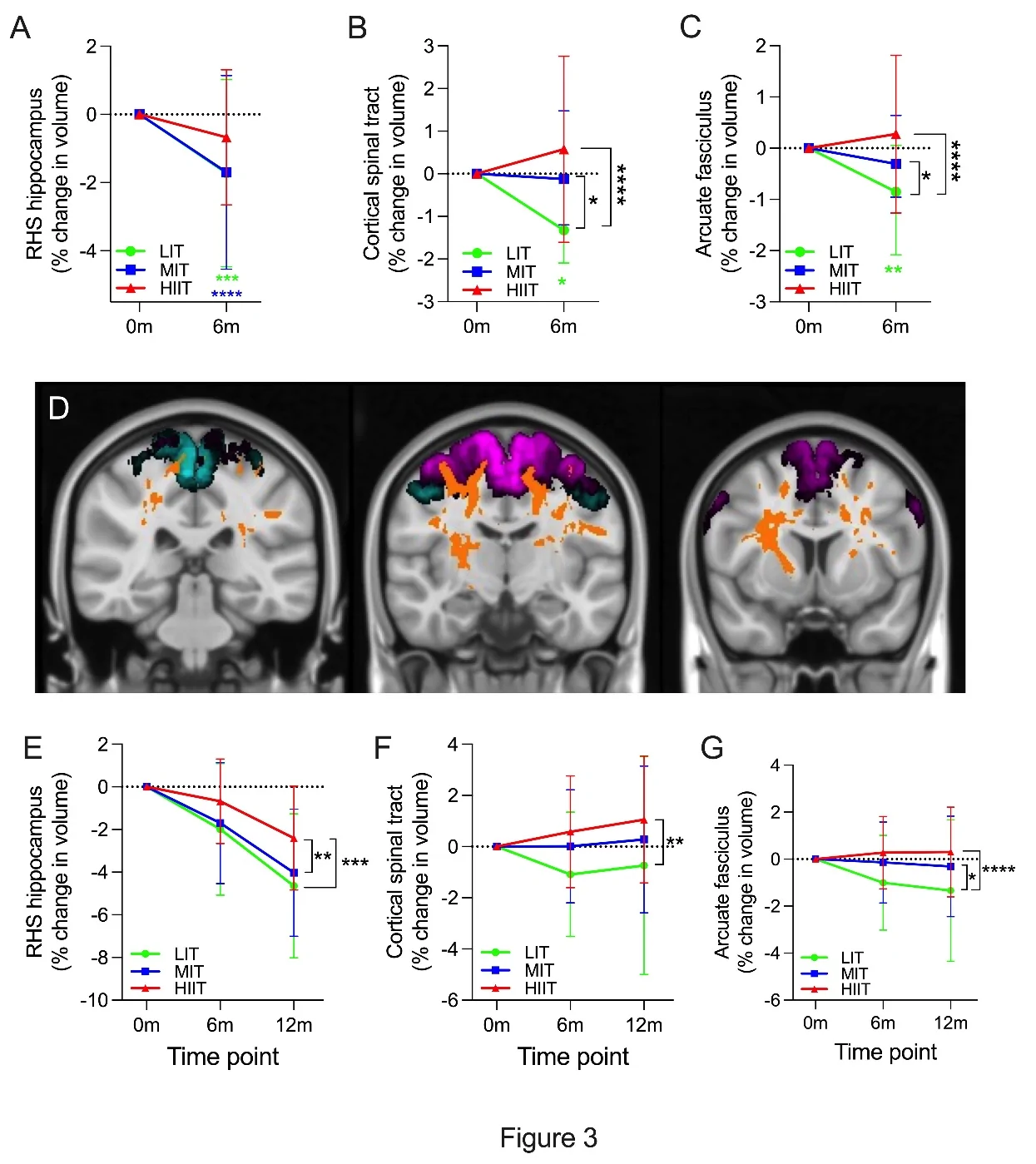
**HIIT exercise prevents volumetric loss in specific brain regions**. (**A**) Comparison of the % change in volume from baseline to the end of the exercise intervention showed that the hippocampal volume from the right-hand side (RHS) remained stable for the HIIT group. However, both the LIT and MIT groups showed a significant reduction (mean±SD; mixed effects model with post hoc tests, *p*< 0.001 and *p*< 0.0001 respectively). (**B**) Following the intervention, the cortical spinal tract remained stable for both the HIIT and MIT groups; however, there was a significant decrease in the LIT group. Differences in % volume change were also observed between the MIT and LIT groups and the HIIT and LIT groups (mean±SD; mixed effects model with post hoc tests [time x exercise intensity effect F (2,214) = 4.71, *p*= 0.01], with Bonferroni post-hoc tests). (**C**) Following exercise there was a significant % difference in the volume of white matter associated with the arcuate fasciculus in the HIIT group compared to both the MIT and LIT groups (mean±SD; mixed effects model with post hoc tests [time x exercise intensity effect F (2,214) = 4.718, *p*= 0.001], with post-hoc tests. (**D**) The increased white matter of both the cortical spinal tract and the arcuate fasciculus, as compared between the HIIT and LIT groups, was shown to connect to the premotor cortex (purple) and the primary motor cortex (cyan) by voxel-wise analysis. (**E**) The amount of volumetric loss in the RHS Hippocampus after 12 months was significantly reduced in the HIIT group compared to both the LIT and MIT groups. (**F**) At the 12-month time point the HIIT group showed an increase in cortical spinal tract volume whereas the LIT showed a decrease, resulting in a significant difference between these groups (*p*= 0.004). (**G**) The arcuate fasciculus remained stable in the HIIT and MIT groups whereas a significant decrease was observed in the LIT group at the 12-month time point (mean±SD; mixed effects model with post hoc tests). Coloured significance indicators are relative to baseline cognitive performance over time for each respective group whereas black significance indicators represent significant differences between groups. *p< 0.05, **p< 0.01, ***p< 0.001, ****p< 0.0001.

In contrast to PALTEA, we found little evidence that exercise affected other cognitive domains including working memory (WM), visual working memory and emotional recognition (Supplementary Fig. 3). The LIT control group showed comparable improvement to MIT and HIIT groups during WM testing for all parameters including total errors (∆WMTE), between trial errors (∆WMBE) and strategy to complete the task (Supplementary Fig. 3A-C), suggesting that these measures were not sensitive to exercise intensity. Visual working memory, as tested by the delayed match to sample (DMS) task, showed no significant difference between groups, and did not show significant improvement during the intervention (Supplementary Fig. 3D-I). In addition, we found no difference in the time required to correctly identify emotional facial cues using the emotional recognition task (ERT) between groups or with time (Supplementary Fig. 3J-O). Taken together, these results show that the HIIT intervention was specific to the hippocampal-dependent spatial learning task.

### Age-related volumetric decreases within specific brain regions are eliminated in response to HIIT

To quantitatively compare, in an unbiased fashion, changes in whole brain volumes that occurred between and within exercise groups, we modified an established deformation-based framework pipeline [25]. We found that, by the end of the 6-month exercise intervention, the right-hand side (RHS) hippocampal volume remained stable in the HIIT group but decreased significantly in both the LIT and MIT groups (Fig. 3A and Supplementary Fig. 4A-C, *p*= 0.0011 and *p*= 0.0002 respectively), whereas all groups showed a significant decrease in volume for the left-hand side hippocampus (Supplementary Fig. 4D-F).

We also found significant decreases in the volume of the corticospinal tract (CST) (Fig. 3B) and the arcuate fasciculus (Fig. 3C) in the LIT group (paired t-tests, *p*= 0.011 and *p*= 0.006 respectively), whereas the volume in both the MIT and HIIT groups remained stable (Supplementary Fig. 4G-I). When examining the longitudinal change for each participant as a change in percentage volume from baseline, we found an exercise intensity-dependent difference in CST volume, with the volume in both the MIT and HIIT groups being significantly larger than that in the LIT group at the completion of the exercise intervention (Fig. 3B; *p*= 0.014 and *p*< 0.0001 respectively). Exercise intensity-dependent, post-exercise changes were also observed for the arcuate fasciculus, with significant differences between the 6-month v baseline difference of LIT compared to MIT and HIIT groups (Fig. 3C and Supplementary Fig. 4J-L; *p*= 0.012 and *p*< 0.0001 respectively), with the LIT group having a significantly higher volume decrease going from the baseline to the 6-month timepoint. Voxel-wise analysis showed significant differences in white matter between the LIT and HIIT group. These areas were shown to be connected to the premotor cortex and the motor cortex (Fig. 3D).

The final MRI scan, at 12 months showed significantly less RHS hippocampal volumetric loss in the HIIT group when compared to both the MIT and LIT groups (Fig. 3E). Similarly, in the HIIT group, the volume of both the CST and arcuate fasciculus remained stable at the 12-month time point whereas significant volumetric loss was observed in the LIT group (Fig. 3F and G respectively).

### Resting-state functional connectivity significantly increases following HIIT exercise

**Figure 4.**
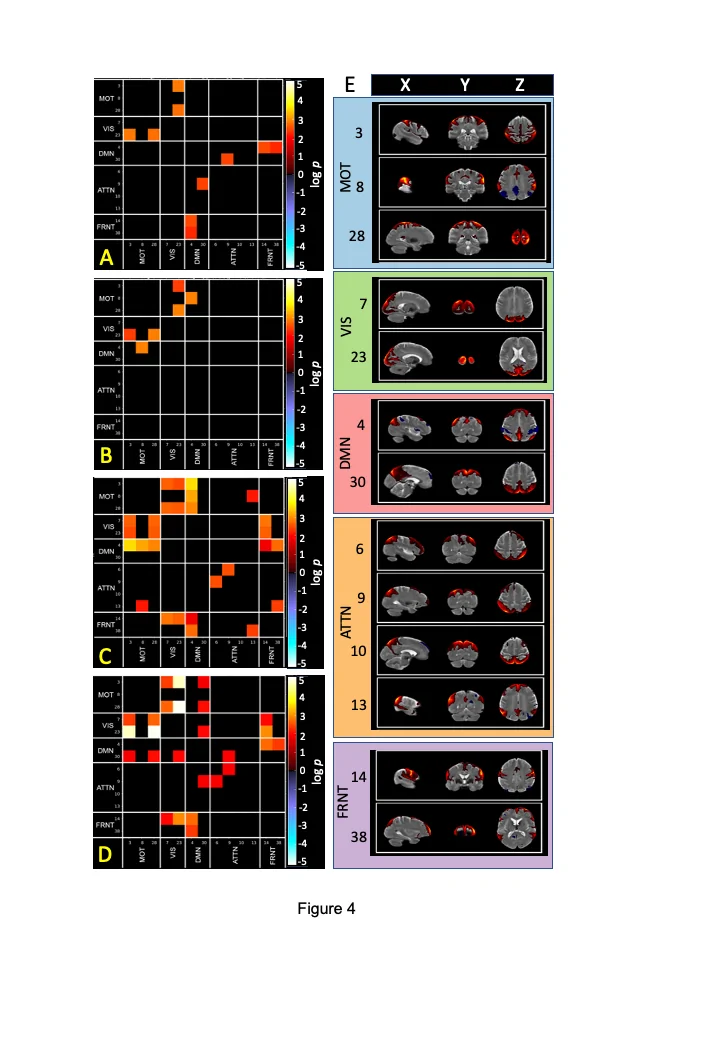
**HIIT exercise improves connectivity between multiple resting-state networks**. (**A**) Comparison of baseline to post-exercise scans revealed FC in the HIIT group significantly increased between network pairs including the DMN-FRNT, DMN-ATTN and VIS-MOT networks when FDR was corrected at Q< 0.2. (**B**) Following exercise, the HIIT group showed increased network FC between the DMN-MOT and VIS-MOT network pairs compared to the LIT group. (**C**) Comparison of the MIT and HIIT groups following exercise revealed increases in connectivity in the HIIT group, including in the DMN-FRONT, DMN-MOT, VIS-FRNT, ATTN-FRNT and VIS-MOT circuits (FDR corrected at Q <0.1). (**D**) Comparison of Group x Time (the change in connectivity between baseline and following exercise) interactions between the LIT and HIIT groups showed that the HIIT group had significant FC increases between the DMN-MOT, DMN-VIS, DMN-FRNT, DMN-ATTN, VIS-MOT and VIS-FRNT networks (FDR corrected at Q< 0.1). (**E**) Representative MRI scans showing the regions of the brain that belong to the independent components (ICs) used to determine the MOT, VIS, DMN, ATTN and FRNT networks. Hot colours represent significant increases in functional connectivity (FC) while cool colours represent a decrease in FC. The X, Y and Z planes are shown for each ICs. Motor (MOT), visual (VIS), default mode (DMN), attention (ATTN) and frontal (FRNT) networks. The numbers listed for each network group represent different anatomical regions of the brain. Significance was calculated as a log *p* value and only those that survived FDR correction were included.

We next examined whether the 6-month exercise intervention affected functional connectivity (FC) during a resting-state scan. When comparing the within-group change in FC strength, we found that only the HIIT group exhibited significant increases between several inter-network pairs including the motor-visual (MOT-VIS), default mode-attention (DMN-ATTN) and DMN-frontal (DMN-FRNT) network following exercise compared to baseline (Fig. 4A). Between group effect changes in FC network pairs were also examined following exercise, with the HIIT group showing increased connectivity between networks in comparison with the LIT (Fig. 4B) and MIT groups (Fig. 4C). In both cases, the HIIT group showed significant increases in the following inter-network FC pairs: MOT-VIS and DMN-MOT (two-way RM-ANOVA; FDR corrected at Q <0.1). Examining the Group x Time effects between exercise groups revealed that the HIIT group exhibited significant increases in FC between numerous network pairs compared to the LIT control group. These included the DMN-MOT, DMN-VIS, DMN-FRNT, DMN-ATTN, VIS-MOT and VIS-FRNT (Fig. 4D; two-way RM-ANOVA; FDR corrected at Q <0.1). The X, Y, Z planes for each independent components (ICs) that showed significant FC interactions are shown in Fig. 4E (all ICs generated are included in Supplementary Appendix 9). Of note, at the 12-month time point, we found no significant differences in FC for any network pairs of the exercise groups relative to baseline (data not shown).

The longitudinal changes to functional connectivity (∆FC) for these regions of interest were then compared to exercise-mediated changes in PALTEA performance (∆PALTEA). The LIT group showed both positive (ATTN06-MOT28) and negative FC interaction (DMN04-VIS23 and FRNT38-ATTN13) changes (Supplementary Fig. 5A) whereas the MIT group demonstrated a single positive interaction (DMN04-VIS23; Supplementary Fig. 5B). The HIIT group, however, had three positive interactions between ∆FC and ∆PALTEA (FRNT14-ATTN06, DMN04-VIS23 and ATTN06-MOT28; Supplementary Fig. 5C).

**Figure 5.**
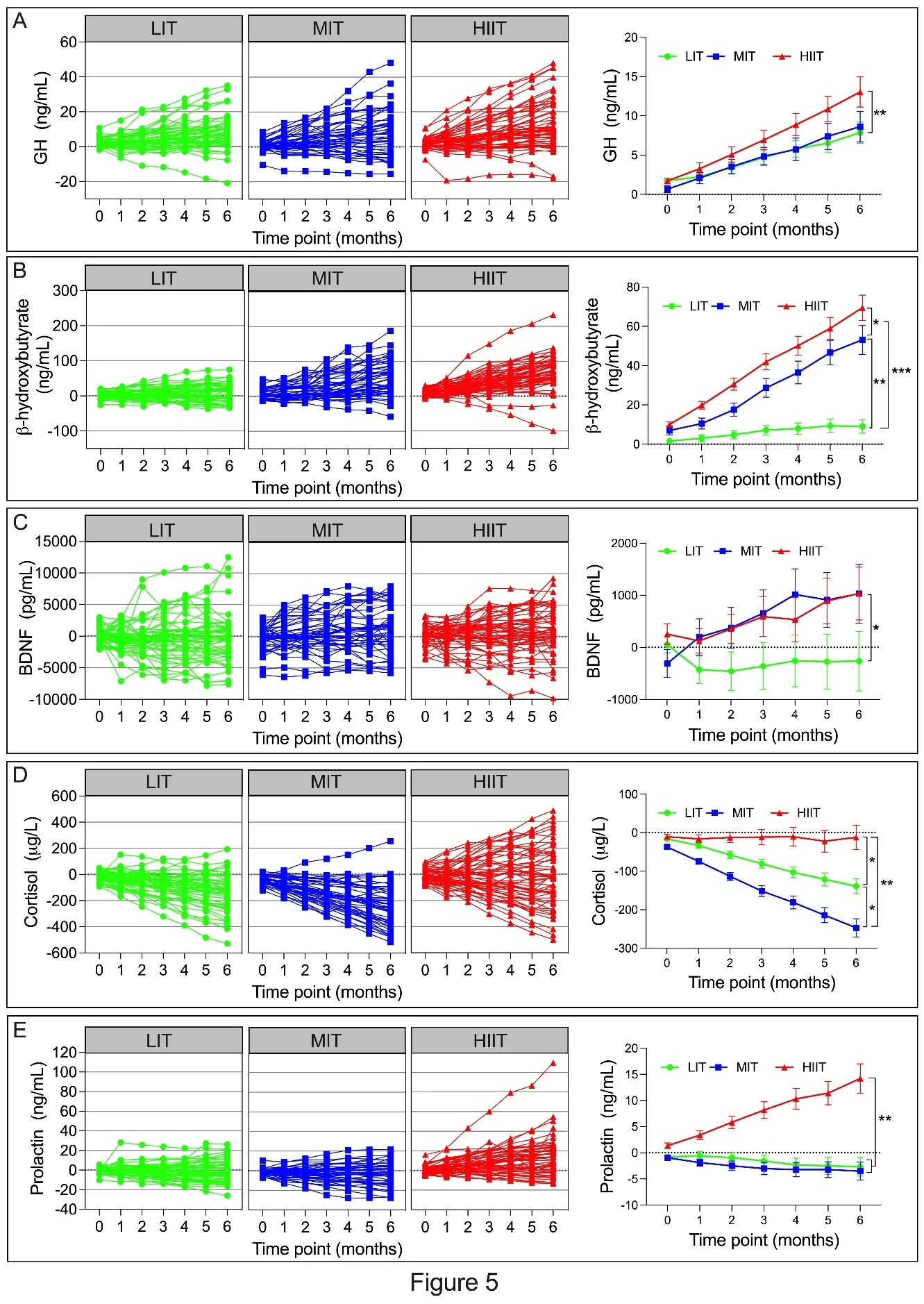
**Cumulative analyte deltas show biochemical and exercise intensity-specific changes**. Tracking individual participants cumulative pre-post exercise deltas (∆) revealed large differences both between and within groups. (**A**) The majority of individuals in each exercise group had a positive cumulative ΔGH value. The cumulative ∆GH slope was significantly higher for the HIIT group than the LIT group (mean±SE; two-way RM-ANOVA [time x exercise intensity effect F (12,888) = 2.802, *p*= 0.0009], with post hoc tests, effect size LIT: HIIT = 0.43). (**B**) Several of the LIT participants had a negative cumulative ∆BHB value whereas the majority of MIT and HIIT individuals had a positive value. The cumulative ∆BHB slope was significantly higher for both the HIIT and MIT groups compared to the LIT group, and the HIIT slope was significantly steeper than that of the MIT group. Following 6 months of exercise there was a significant difference in ∆BHB between groups (mean±SE; two-way RM-ANOVA [time x exercise intensity effect F (12,870) = 20.39, *p*< 0.0001], with post hoc tests, effect sizes LIT: HIIT = 1.62, LIT: MIT = 1.13). (**C**) There was a large spread in cumulative ΔBDNF between participants for each exercise intensity. The cumulative ∆BDNF slope for the LIT group was close to zero after 6 months of exercise whereas both the MIT and HIIT groups had positive values (mean±SE; two-way RM-ANOVA [time x exercise intensity effect F (12,883) = 1.814, *p*= 0.042], with post hoc tests, effect size LIT: HIIT = 0.33). (**D**) The majority of participants in the MIT and LIT groups had a negative cumulative ∆cortisol value whereas the HIIT group had a large spread in cumulative values. Both the LIT and MIT groups had significant negative cumulative ∆cortisol slopes whereas that of the HIIT group remained steady (mean±SE; two-way RM-ANOVA [time x exercise intensity effect F (12,888) = 16.63, *p<* 0.0001], with post hoc tests, effect sizes LIT: HIIT = 0.68, MIT: HIIT = 1.18). (**E**) The LIT and MIT participants were evenly distributed with negative and positive cumulative ∆prolactin values whereas the majority of HIIT participants had positive ∆prolactin levels. Both the LIT and MIT cumulative ∆prolactin levels remained stable over 6 months whereas the HIIT group exhibited a significant positive slope (mean±SE, two-way, RM-ANOVA [time x exercise intensity effect F (12,888) = 17.16, *p<* 0.0001], with post-hoc tests, effect sizes LIT: HIIT = 0.99, MIT: HIIT = 1.07). * *p*< 0.05, ** *p*< 0.01, and *** *p*< 0.001.

### Circulating biomarkers differentially respond to exercise intensity

To examine any biochemical changes accompanied by the different intensities of exercise and may be associated with the observed hippocampal-dependent cognitive improvement, we next analysed several circulating factors, some of which have previously been reported to be modulated by exercise in humans and/or animal models. Of the 17 analytes initially examined (see Supplementary Table 1), only those exhibiting exercise-mediated changes were analysed further (Supplementary Fig. 6). Both growth hormone (GH) and β-hydroxybutyrate (BHB) showed consistent post-exercise increases relative to pre-exercise levels for all groups (Supplementary Fig. 6A-B respectively) while cortisol, prolactin and BDNF showed post-exercise responses that were dependent on exercise intensity (Supplementary Fig. 6C-E respectively).

We also calculated the cumulative pre-post exercise delta value (∆) at each month for each biomarker (Fig. 5). This approach provides insight into the exercise-mediated longitudinal changes in circulating biomarkers at both the individual and group level. We found that cumulative ∆GH increased for all groups during the exercise intervention, with the largest increase occurring in the HIIT group. This value was significantly higher than that of either the LIT or MIT group at the completion of the intervention (Fig. 5A). The longitudinal change in cumulative ∆BHB, however, displayed an exercise intensity-dependent increase (Fig. 5B): with a negligible change observed in the LIT group, a significant increase in the MIT group, and exhibiting the highest cumulative increase in ∆BHB in the HIIT group. In the case of BDNF, which has been extensively examined for its association with exercise-mediated changes and cognition, we found that MIT and HIIT produced a similar increase in ∆BDNF whereas the ∆BDNF levels remained stable in the LIT group (Fig. 5C). Examination of the changes in cortisol levels revealed that most LIT and MIT participants showed a decrease in ∆cortisol levels whereas HIIT participants were evenly split between increased and decreased cortisol levels and so remained stable as a group overall (Fig. 5D). An increase in cumulative ∆prolactin levels was restricted to the HIIT group whereas the levels in both the LIT and MIT groups remained stable during the exercise intervention (Fig. 5E). Inflammation markers including IL-1β, IL-6, IL-10, IL-12 and IFNγ were also examined (Supplementary Fig. 7) and showed no significant differences between groups over the duration of the exercise intervention.

### Exercise-mediated changes in cortisol predict PAL ability and correlate to endpoint hippocampal cognitive function in HIIT participants

**Figure 6.**
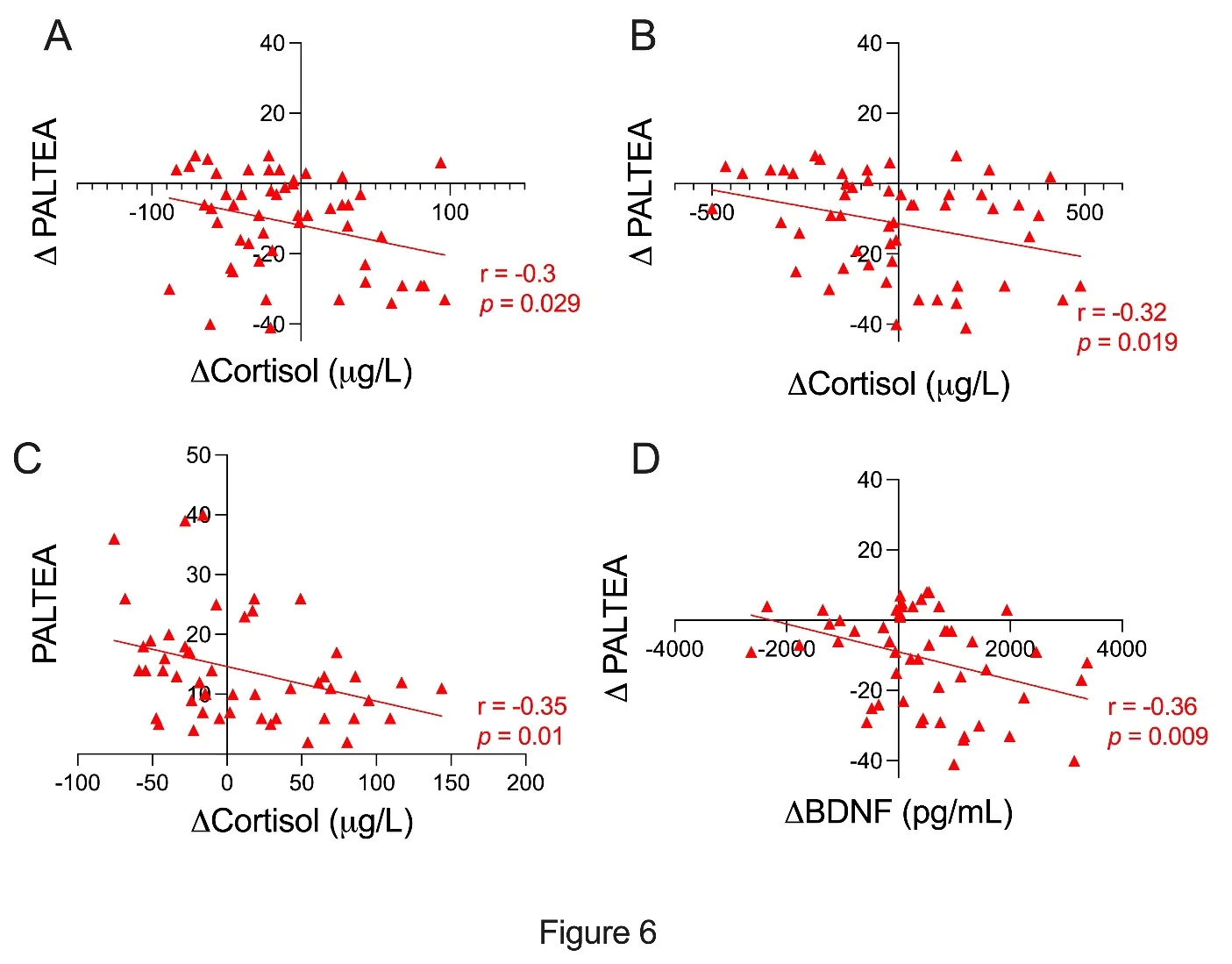
**Pre/post exercise delta values for cortisol and BDNF predict improved learning ability for the HIIT group**. (**A**) The HIIT group showed a significant correlation between the initial exercise-mediated cortisol pre/post delta values (∆) and learning ability (∆PALTEA), with higher cortisol values correlating to improved learning. (**B**) The total cumulative ∆cortisol levels at 6 months showed that higher ∆cortisol levels correlated to better learning ability for the HIIT group. (**C**) The 6-month pre/post delta Δcortisol levels correlated to superior endpoint cognitive ability for the HIIT group. (**D**) The HIIT group showed a significant correlation between the initial, pre/post-exercise-mediated ΔBDNF values and PALTEA learning ability. Spearman correlations were used for analysis.

We next determined if correlations between circulating analytes and hippocampal cognitive function were present. Only those that showed significance are presented here. Restricted to the HIIT group, we found a significant correlation between the initial pre-post exercise delta value of cortisol (∆cortisol) and improved PALTEA performance at the end of the exercise intervention (Fig. 6A). When the delta between pre/post-exercise cortisol levels across the entire period of the exercise intervention was accumulated, we found a significant positive correlation with improved PALTEA learning for the HIIT group (Fig. 6B). We also found that the 6-month pre/post-exercise delta value for cortisol positively correlated to the endpoint PALTEA performance for the HIIT group (Fig. 6C).

### The initial change in BDNF predicts hippocampal cognitive improvement for HIIT participants

In the HIIT group, we found no relationship between the absolute levels of circulating BDNF and PALTEA performance for either pre- or post-exercise samples (Supplementary Fig. 8A-E) until the 6-month timepoint where a higher post-exercise BNDF concentration correlated to better PALTEA ability (Supplemenatry Fig. 8F). Surprisingly, when we compared the exercise-mediated difference between pre-and post-exercise BDNF (∆BDNF), we found that higher initial ∆BDNF concentrations significantly correlated with improved PALTEA learning (∆PALTEA) at the end of the trial (Fig. 6D). This correlation remained significant after 1 month of exercise (Supplementary Fig. 9A) and there was a strong trend for an association between higher cumulative ∆BDNF concentration and improved ∆PALTEA learning which was maintained until the end of the exercise intervention (Supplementary Fig. 9). No correlations between BDNF and PALTEA were observed for the LIT or MIT groups (data not shown).

### Decreased prolactin levels in the MIT group correlate to improved hippocampal cognitive function

**Figure 7.**
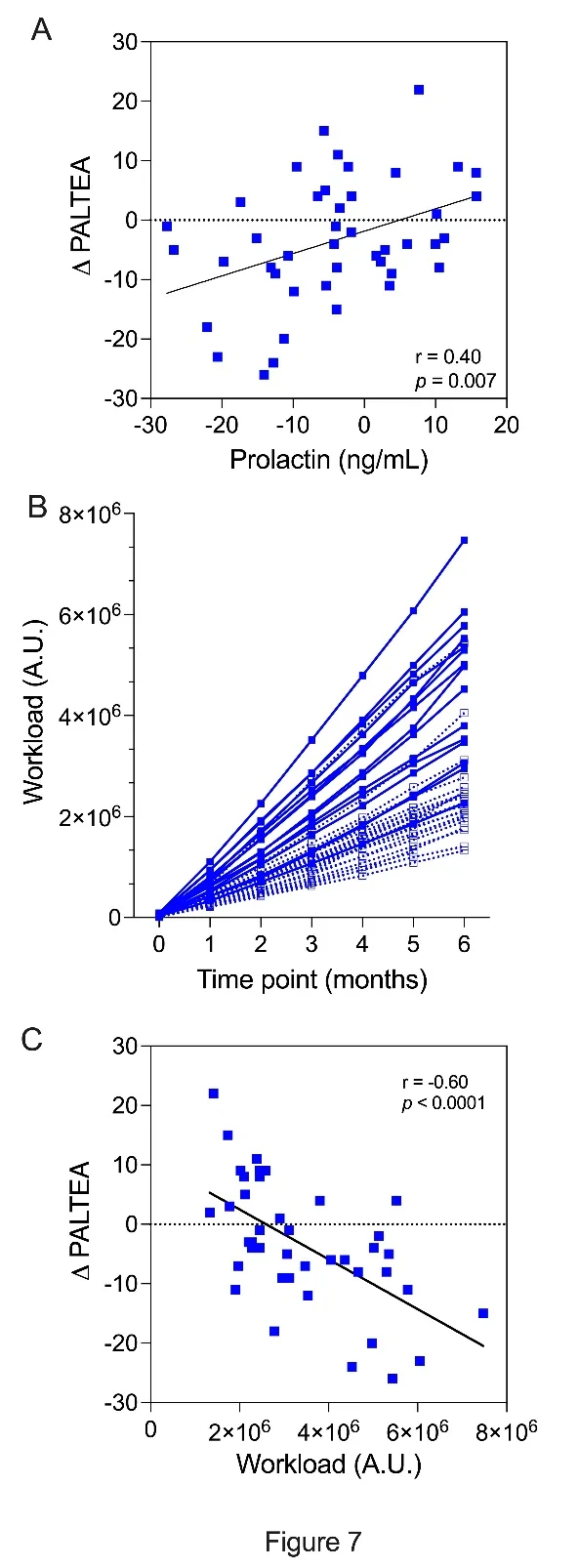
**Lower prolactin levels and higher cumulative exercise workload correlate to improved cognition in MIT participants**. (**A**) Exercise-mediated changes in total cumulative Δprolactin correlated to improved PALTEA performance for the MIT group. (**B**) Individual MIT workload levels subdivided based on gender, with solid lines representing males and dotted lines representing females. (**C**) The total cumulative workload after 6 months of exercise for the MIT group significantly correlated to ∆PALTEA change with higher total workload correlating to better PALTEA improvement. Spearman correlations were used for analysis.

For the MIT group alone, we found that exercise-mediated changes in cumulative ∆prolactin levels correlated to improved PALTEA performance (Fig. 7A). This relationship emerged at 2 months, was strongest at 4 months and was maintained throughout the remainder of the exercise intervention (Supplementary Fig. 10A-F). We then conducted a subgroup analysis for the MIT group using a change of ±1SD PALTEA performance to divide the MIT group into improvers, stable participants and deteriorators. This showed that those participants with improved hippocampal cognition had significantly lower prolactin levels than those that deteriorated (Supplementary Fig. 10G).

### Higher workload levels correlate with improved PAL performance

Controlling exercise dose is typically accomplished by reaching and maintaining specified HR ranges, an approach we also used. However, controlling for target HR necessarily results in different mechanical work for each participant. As multiple parameters, including HR, treadmill speed and treadmill angle, were recorded every session, we were able to accurately calculate the exercise workload throughout the trial. Calculation of the workload in this manner clearly revealed the difference in the total exercise workload that each participant completed during the intervention for both the HIIT (Supplementary Fig. 10H) and MIT groups (Fig. 7B). Although we did not find a correlation between HIIT exercise workload and improved cognitive function (data not shown), the MIT group exhibited a strong correlation between higher workload levels and improved PALTEA performance (Fig. 7C).

## DISCUSSION

This is the first study, to our knowledge, to reveal that a 6-month HIIT intervention significantly improves hippocampal-dependent spatial learning, as measured by PAL, in healthy, aged human individuals. At the completion of the exercise intervention PAL in the HIIT group had improved significantly from baseline as well as when compared to both the LIT and MIT groups. Remarkably, the hippocampal-dependent PAL improvement in the HIIT group was retained for up to 5 years. The non-hippocampal-dependent WM cognitive tests failed to show significant differences between exercise groups whereas visual working memory and ERT revealed no improvement during the testing period. High-resolution MRI showed that HIIT abrogated age-dependent volumetric decreases in specific brain regions, including the hippocampus, which is especially sensitive to aging. Resting-state MRI indicated that the HIIT-mediated hippocampal-dependent cognitive improvement was associated with enhanced functional connectivity between key networks, including the DMN and those associated with attention, motor function and vision. Biomarker analysis revealed a variety of exercise-mediated changes; however, only increased BDNF and cortisol levels correlated to improved hippocampal-dependent cognitive function in the HIIT group, whereas a decrease in prolactin correlated to improved hippocampal-dependent cognition in the MIT group.

An age-dependent decrease in hippocampal function has been clearly established in both humans [4, 26] and rodents [7, 8] and we chose PAL to assess the potential for exercise to ameliorate this loss because PAL is a hippocampal-dependent cognitive paradigm [27] that is sufficiently sensitive to identify those at risk of developing dementia [28, 29]. Importantly, the test-re-test reliability of the computerized and automated PAL test we used is robust and falls into the acceptable to high range [30, 31]. At the conclusion of the 6-month exercise intervention, only the HIIT group showed significantly improved PAL performance, whereas both the MIT and LIT groups remained stable. Previous studies examining hippocampal-dependent cognitive changes following MIT also reported no improvement when using neuropsychological tests including the mini-mental state examine (MMSE) [32, 33] or Montreal cognitive assessment (MoCA) [34, 35], which are unlikely to possess the required sensitivity to discriminate subtle changes [27], especially within cognitively healthy individuals. Similarly, an electronic match/non-match spatial learning task following MIT exercise did not detect significant improvements above controls [14]. Initial PAL scores allowed us to examine the effect of exercise on those who may be most at risk of developing cognitive impairments, namely those that began the intervention more than 1SD above the median performance. Encouragingly, we found that they showed the greatest improvement in PAL performance with HIIT; moreover, they also responded to MIT, although to a much lesser degree, suggesting that even moderate exercise may be beneficial to this at-risk group.

Surprisingly, the improvements in hippocampal performance following HIIT exercise were not observed with other cognitive domains such as WM, visual working memory or emotional recognition, where we found minimal changes and no significant differences between the groups at the conclusion of the 6-month exercise period. The lack of an exercise intensity-mediated effect on working memory was of interest given that it has been the focus of several studies and meta-analyses with some concluding no effect [36] while others found only small effect sizes [37]. A lack of sensitivity in WM cognitive tests may have also contributed to this result as a recent study found that PAL was superior to WM at successfully discriminating cognitive abilities in high functioning elderly individuals [38]. More studies are therefore required to determine whether exercise can indeed mediate WM or if more sensitive and challenging cognitive tests are required for determining subtle differences over time. Regardless, our results highlight the specificity of HIIT in significantly improving hippocampal-dependent spatial learning in the healthy elderly.

The maintenance of hippocampal function in the active control LIT group for up to 5 years was an intriguing observation, especially as this contrasts to previous reports that multiple cognitive domains significantly decline within a 4 year re-test period during normal aging [39]. A possible explanation for this may be associated with the improvement in balance that occurred during the intervention. Indeed, it has been demonstrated previously that a 12-week balance training paradigm was sufficient to improve spatial learning, even in the absence of altered cardiorespiratory fitness [40]. Thus our 6-month, LIT motor function and balance regimen may be a viable approach to maintain cognitive function for participants that have reduced physical ability and movement limitations.

Another major aim we addressed with our study is the long-term effectiveness of an exercise intervention [41], which has significant ramifications for the trajectory of cognitive decline [42]. As exercise at high intensity becomes increasingly difficult in older individuals, intervention at a younger age that has a significant impact on long-term performance becomes increasingly important. Our results demonstrate that a relatively acute, 6-month HIIT intervention can maintain the improvement in hippocampal function for at least 4.5 years with no diminution of performance. Importantly, this was observed even though there was no discernable change to the original physical or lifestyle practice of the participants once the exercise intervention was complete. Nor was there a significant change in cardiorespiratory fitness during the intervention, indicating that this did not play a major role in improving hippocampal function.

It is well established that there is an age-associated decrease in whole brain volume [43, 44] and the hippocampus is especially susceptible to volumetric decrease [5, 43], with an annual hippocampal volumetric loss between 1.4-2.81% being reported [21, 45] which increases beyond 65 years of age [5]. A concomitant decrease in specific domains of cognitive function such as executive function and spatial learning is also typically reported [21, 46]. Using high-resolution 7T MRI we found that HIIT maintained hippocampal volume at 6 and 12 months whereas both the LIT and MIT groups exhibited significant volumetric decreases at the completion of the exercise intervention. This effect was most profound in the right hippocampus which has been identified as having a role in spatial learning [47]. In support of this, while one study previously reported a significant increase in hippocampal volume in healthy aged individuals [14] following 12 months of MIT exercise, our findings are more in line with the meta-analytic examination of the literature which shows retention of volume rather than an overall increase [48] is more likely.

As with our hippocampal findings, only HIIT mitigated the age-associated decrease observed in the white matter tracts for the CST and arcuate fasciculus. The CST, which connects the motor cortex to the spinal cord to enable voluntary control of the distal extremities, decreases during age [49]. This is the first report of the effect of exercise on CST volume in the elderly. We also found that the arcuate fasciculus was maintained following HIIT exercise as opposed to decreases observed in the LIT and MIT groups. The arcuate fasciculus is a bundle of axons that connects the temporal and inferior parietal cortices to the frontal temporal lobe and is involved in producing and understanding language. Interestingly, it has been reported that healthier middle aged people exhibit higher fasciculus integrity compared to age-matched sedentary individuals [50], although a 6-month MIT intervention failed to improve the integrity of these tracts in aged individuals [51]. These results indicate that HIIT exercise ameliorates the age-dependent decrease in volume observed in multiple regions including the hippocampus and arcuate fasciculus, both of which have been associated with learning [21] and memory [52].

It has been suggested that changes in the functional organization of the brain may precede both structural differences and clinical symptoms, especially in relation to disorders such as Alzheimer’s disease [53]. Synchronization of blood oxygenation level dependent (BOLD) signals in the absence of explicit stimuli between regions is referred to as resting-state MRI [6], which provides a measure of FC both within and between networks. In aged mice, we recently demonstrated significant decreases in FC within the hippocampus which could be reversed via an exercise-mediated increase in hippocampal neurogenesis [12]. We therefore examined if FC was also altered during exercise in our cohort of elderly participants. We found increases in FC between the DMN-ATTN and DMN-FRNT networks in the HIIT group, suggesting that these changes contributed to the observed improvement in PAL performance. This is supported by our recent finding that exercise in aged mice increases FC between the entorhinal cortex [12], which is part of the ATTN network [54], and the hippocampus (included in the DMN), resulting in improved performance in hippocampal-dependent spatial learning [12]. Further, in young adults, an increase in FC within the ATTN network correlates to improved spatial cognitive tasks [55]. Taken together, these findings demonstrate that HIIT exercise increases the FC between networks that have been identified to be either altered during aging or play a role in cognitive function [6, 55, 56]. Considering the similarities in brain structure and function between rodents and humans, our studies in aged mice following exercise showing increased hippocampal neurogenesis [8, 12], provide crucial mechanistic insight as to how exercise in humans may abrogate the age-associated decrease in hippocampal cognitive function by reducing the trajectory of hippocampal volume decrease and improve FC between key networks.

To gain insight into putative humoral mechanisms which may be associated with, or mediate the improved hippocampal cognitive function, multiple studies have explored various circulating biomarkers in humans and animals [14, 15, 57]. Foremost amongst these analytes is the neurotrophin BDNF. In animal models, circulating levels of BDNF have been reported to directly correlate to those in the hippocampus [58]. This, combined with the established role of BDNF in neurogenesis and synaptic plasticity, makes circulating BDNF an attractive potential biomarker of human cognitive health [14, 19]. Considering that humans [15] and animals [8] both show exercise-mediated increases in circulating BDNF, similar mechanisms may be involved in mediating cognitive improvement. For example, animal studies have shown that an exercise-mediated increase in BDNF, specifically in hippocampal tissue, directly correlate to improved hippocampal-dependent spatial learning performance [59] as well as increased neurogenesis [60], which we and others have shown to be critical for hippocampal-dependent spatial learning [8, 12, 61].

Our approach of calculating the acute exercise-mediated changes in BDNF (∆BDNF) revealed a similar increase in BDNF for both the MIT and HIIT groups at the completion of the exercise intervention. A similar study found that a single session of MIT or HIIT exercise in middle aged and elderly participants resulted in BDNF increasing to the same degree; however, no correlation between pre- or post-exercise BDNF and cognitive function was observed [62]. Interestingly, we found that in the HIIT, but not the MIT or LIT groups, it was the difference between pre- and post-exercise BDNF levels (∆BDNF) that predicted improved hippocampal-dependent learning ability in the HIIT group at the end of the exercise program rather than the absolute concentration of either the pre- or post-exercise circulating BDNF levels. As we observed this correlation from the start of the exercise program, it implies an intrinsic capacity of the individuals to respond to HIIT. This response is unlikely to be associated with a previous history in exercise as we found no evidence of a relationship between initial concentrations of BDNF and initial hippocampal cognitive ability. Indeed, preliminary data suggest that the observed correlations between higher initial ∆BDNF and ∆cortisol levels with improved PALTEA performance may be due to primed epigenetic factors that allow for immediate responses to external stimuli, a prospect we are currently investigating.

As with our recent study in aged mice [8], we found no change in circulating inflammation markers during exercise in all groups and did not observe any correlations between inflammation markers and hippocampal cognitive function. Low level chronic inflammation has often been associated with advanced age [63]; however the effect of exercise on circulating cytokines remains to be fully elucidated, as highlighted in recent meta-analyses [63-65]. While this remains an area for further examination, it suggests that modulation of inflammatory cytokines may not play a crucial role in the observed effects of exercise.

Chronically elevated cortisol levels have been associated with deficits in cognition, particularly in the aged population [66]; however, acute elevations in cortisol induced by physiological stimuli such as exercise have also been reported to be beneficial [57]. We found that the HIIT intervention maintained total ∆cortisol levels whereas a decrease was observed in both the LIT and MIT groups. One study examined the effect of vigorous physical activity on cortisol and quality of life (QoL) outcomes and found that a moderate, non-pathological increase in cortisol was associated with a better perception of QoL [57]. Focusing on the exercise-mediated change in cortisol, we found that a higher initial ∆cortisol level predicted improved PAL performance and a higher total Δcortisol correlated with end-point cognitive ability for the HIIT group. Taken together, these results suggest that initial exercise-mediated changes in both BDNF and cortisol appear to be good candidates to predict the effectiveness of HIIT in improving cognitive function in the healthy elderly. How the exercise-dependent increase in cortisol mediates hippocampal-dependent cognitive improvement, however, remains to be elucidated. There is some suggestion that cortisol acts indirectly by modulating BDNF gene expression [67], whereas others have shown that acute cortisol exposure enhances neurogenesis and hippocampal-dependent learning in rodent models [68].

The divergence in hippocampal learning response, coupled with the exercise intensity-dependent differences of multiple circulating factors between ostensibly similar exercise interventions (MIT and HIIT) suggests that some components of the HIIT intervention is intrinsically different from more traditional steady-state exercise. A lack of significant differences in cardiorespiratory fitness between MIT and HIIT during the intervention suggests mechanisms other than those related to cardiorespiratory fitness are involved. One major difference between the interventions is the exercise workload experienced, especially by the leg skeletal muscles. Importantly, myokines which are factors released following skeletal muscle contractions [69] in an exercise intensity-dependent manner, have been associated with improvements in hippocampal function [70]. Indeed, BDNF is one of the most extensively studied myokines in relation to brain function [71].

More recently, it has been found that the exercise induced secretion of factors extends beyond skeletal muscle to other organs and is now collectively referred to as exerkines [72]. In support of a role of exerkines in hippocampal-dependent learning, we recently demonstrated that the exerkine platelet factor 4 was effective at increasing hippocampal neurogenesis and cognitive function in old mice [73]. With multiple organs now shown to be sensitive to exercise, the level of crosstalk and signaling cascades becomes far more complex. For example, β-hydroxybutyrate, which we showed increased in an exercise intensity-dependent manner, was originally described as an alternate energy source secreted from the liver for use by peripheral organs including the brain [74]. More recently however, β-hydroxybutyrate has been shown to have additional downstream effects, including changes to BDNF expression [75]. Similarly, our observed exercise intensity-mediated release of cortisol from adrenal glands, and growth hormone and prolactin from the pituitary gland, fall into this definition of exerkines. Collectively, our findings add to the expanding identification of exercise-mediated metabolite release from multiple organs and underscore the emerging picture of complexity required to positively influence hippocampal function. Additionally, the physiological switch between aerobic and anaerobic exercise and the effect on myokines and exerkines has yet to be thoroughly explored.

In line with differential responses between aerobic and anaerobic exercise, we noted a difference in response between the MIT and HIIT groups for Prolactin. In this instance, an exercise-mediated decrease in ∆prolactin correlated to improved PAL performance, a finding that was restricted to the MIT group. This is likely due to the exercise-intensity mediated switch that has been observed in young adults [76] in which prolactin decreases following moderate intensity exercise but increases when anaerobic, high intensity exercise is undertaken. Our finding suggests that the physiological switch in prolactin secretion patterns due to exercise intensity is retained in the elderly.

The differential response in prolactin and other biomarkers between the MIT and HIIT groups highlights the existence of exercise intensity-dependent physiological responses. This is likely due to the switch between aerobic and anaerobic exercise. In our study, MIT exercise was purely aerobics whereas HIIT included a combination of both aerobic and anaerobic components. The switch between aerobic and anaerobic exercise is an important distinction that has not received the appropriate level of attention, and future studies should consider this variable when analysing circulating biochemical markers. Taken together, we would argue that the exercise-mediated improvement in hippocampal function requires the complex orchestration of multiple organs and mechanisms, some of which we and others have identified, while others have yet to be discovered and will require further studies to be fully elucidated.

### Limitations

Our study was highly supervised to ensure that participants conducted the appropriate exercise level. Regardless of this, there were limitations. For example, this study only examined the effect of exercise on aged individuals that did not exhibit cognitive deficits. We also did not include an isolated non-exercise, sedentary control group to determine the effects of social interactions alone may have on performance. Selection of participants was designed to include healthy aged individuals capable of participating in a 6-month exercise intervention. It therefore remains to be seen if this type of exercise intervention would be possible in less physically healthy individuals. Further, it is currently unknown if other modalities of HIIT such as low impact exercise bikes or rowing machines would provide similar hippocampal-dependent cognitive improvements.

### Conclusions

This is the first study to our knowledge to identify that a HIIT paradigm for the healthy elderly is suitable and effective at significantly improving and retaining long-term hippocampal-dependent learning, for up to 5 years. Our finding of individualized, exercised-mediated responses of biomarkers as predictors for improved hippocampal functional outcomes offers a quantifiable metric to provide an effective exercise regimen. The improvement, and long-term retention of hippocampal learning ability following HIIT exercise provides a new insight into how the elderly could be insulated from cognitive decline even though their exercise capabilities may decline with advanced age. This approach could greatly enhance the capacity of clinicians to tailor personalized exercise paradigms, including those at risk of cognitive decline.

## Supplementary Materials

The Supplementary data can be found online at: www.aginganddisease.org/EN/10.14336/AD.2024.0642.

## Current Affiliations

Maryam Ziaei: Kavli Institute for Systems Neuroscience and Jebsen Centre for Alzheimer's disease, Norwegian University of Science and Technology, Trondheim, Norway

Elizabeth J. Cooper: Australian eHealth Research Centre, CSIRO, Herston, QLD 4029

Thomas Shaw: School of Electrical Engineering and Computer Science, UQ. Centre for Advanced Imaging, UQ

Emily Hume: University of Southern Queensland, School of Health and Medical Sciences, Ipswich.
