# Supplementary material for: Long-Term Improvement in Hippocampal-Dependent Learning Ability in Healthy, Aged Individuals Following High Intensity Interval Training
Source: Aging Dis. 2024 Jun 27;16(3):1732–54. doi: 10.14336/AD.2024.0642 (PMC12096939; doi:10.14336/AD.2024.0642)

Long-Term Improvement in Hippocampal-Dependent Learning Ability in Healthy, Aged Individuals Following High Intensity Interval Training

Daniel G. Blackmore, Mia A. Schaumberg, Maryam Ziaei, Samuel Belford, Xuan Vinh To, Imogen O'Keeffe, Anne Bernard, Jules Mitchell, Emily Hume, Grace L. Rose, Thomas Shaw, Ashley York, Markus Barth, Elizabeth J. Cooper, Tina L. Skinner, Fatima Nasrallah, Stephan Riek, Perry F. Bartlett

© 2024. Blackmore DG et al. Published online at http://www.aginganddisease.org/EN/10.14336/AD.2024.042

SUPPLEMENTARY DATA

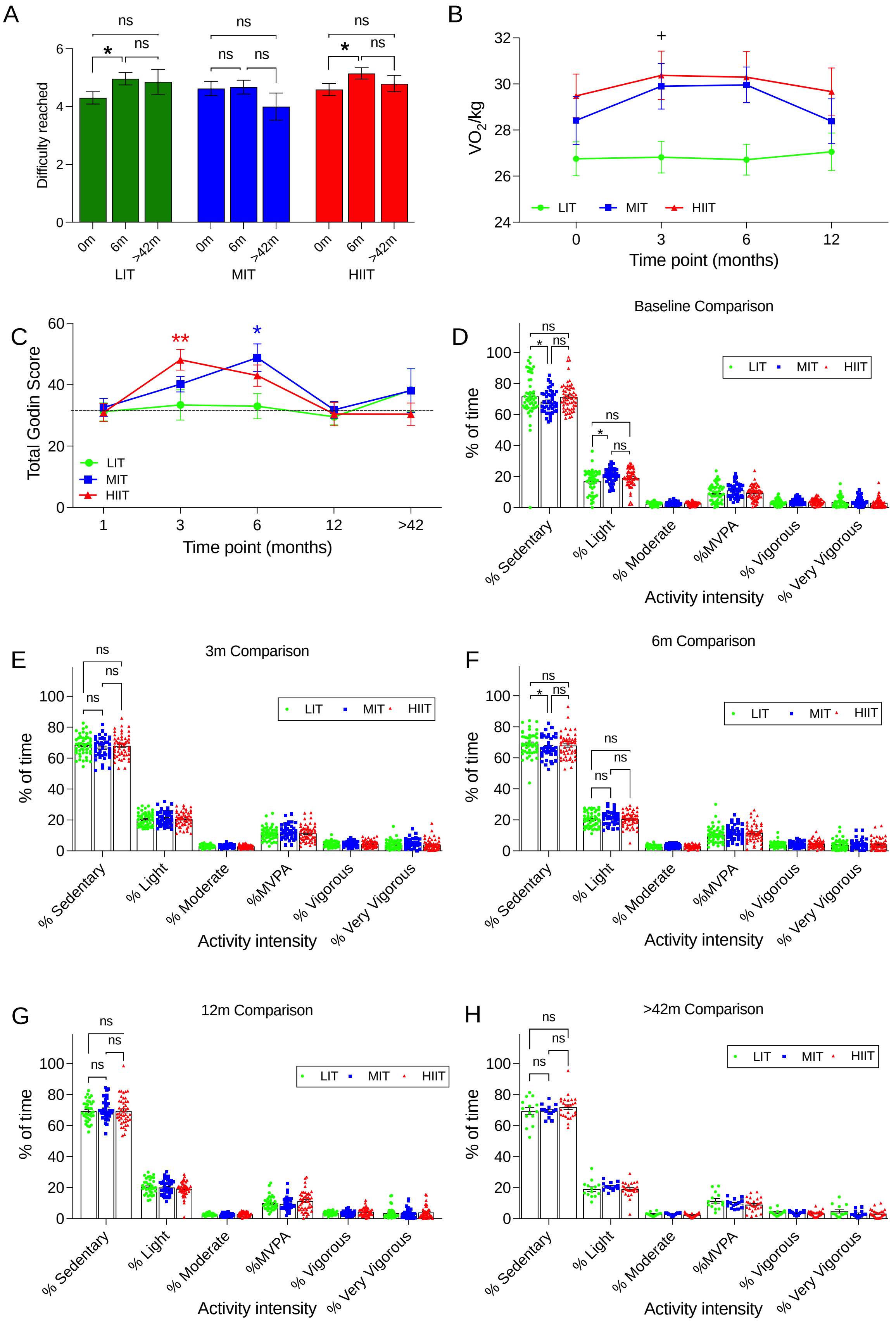

© 2024. Blackmore DG et al. Published online at http://www.aginganddisease.org/EN/10.14336/AD.2024.042

Supplementary Figure 1. Cardiorespiratory fitness and physical activity for participants remains relatively stable both during exercise training and following the intervention. (**A**) Balance improved for both the LIT and HIIT groups following 6-months of exercise (mean±SE; two-way RM-ANOVA [time effect F (2,199) = 5.75, *p* = 0.0037] with Bonferroni post-hoc tests). (**B**) There was a main effect of exercise intensity for $V0_2$/kg during the exercise intervention and following the trial (mean±SE; two-way RM-ANOVA [exercise intensity effect F (2,149) = 4.675, *p* = 0.011). Only the 3-month test period showed a significant difference between the LIT and HIIT group (post hoc test, *p* = 0.0413). (**C**) The Godin leisure activity questionnaire revealed a self-reported increase in total leisure activity for the HIIT and MIT groups at the 3- and 6-month periods respectively. However, all groups returned to baseline levels after the completion of the intervention and then remained stable. (**D**) Before the initiation of exercise, participants from the MIT group spent significantly more time sedentary compared to the LIT group. (**E**) At the 3-month timepoint there were no differences on activity between the groups (**E**). (**F**) At the 6-month timepoint the MIT group showed more sedentary time compared to the LIT group. (**G**) Following the exercise intervention, all groups had similar physical activity profiles at the 12-month time point. (**H**) No difference in activity was also observed in the long-term follow up (mixed effects model with Bonferroni post hoc tests). *p< 0·.5, and **p< 0.01.

© 2024. Blackmore DG et al. Published online at http://www.aginganddisease.org/EN/10.14336/AD.2024.042

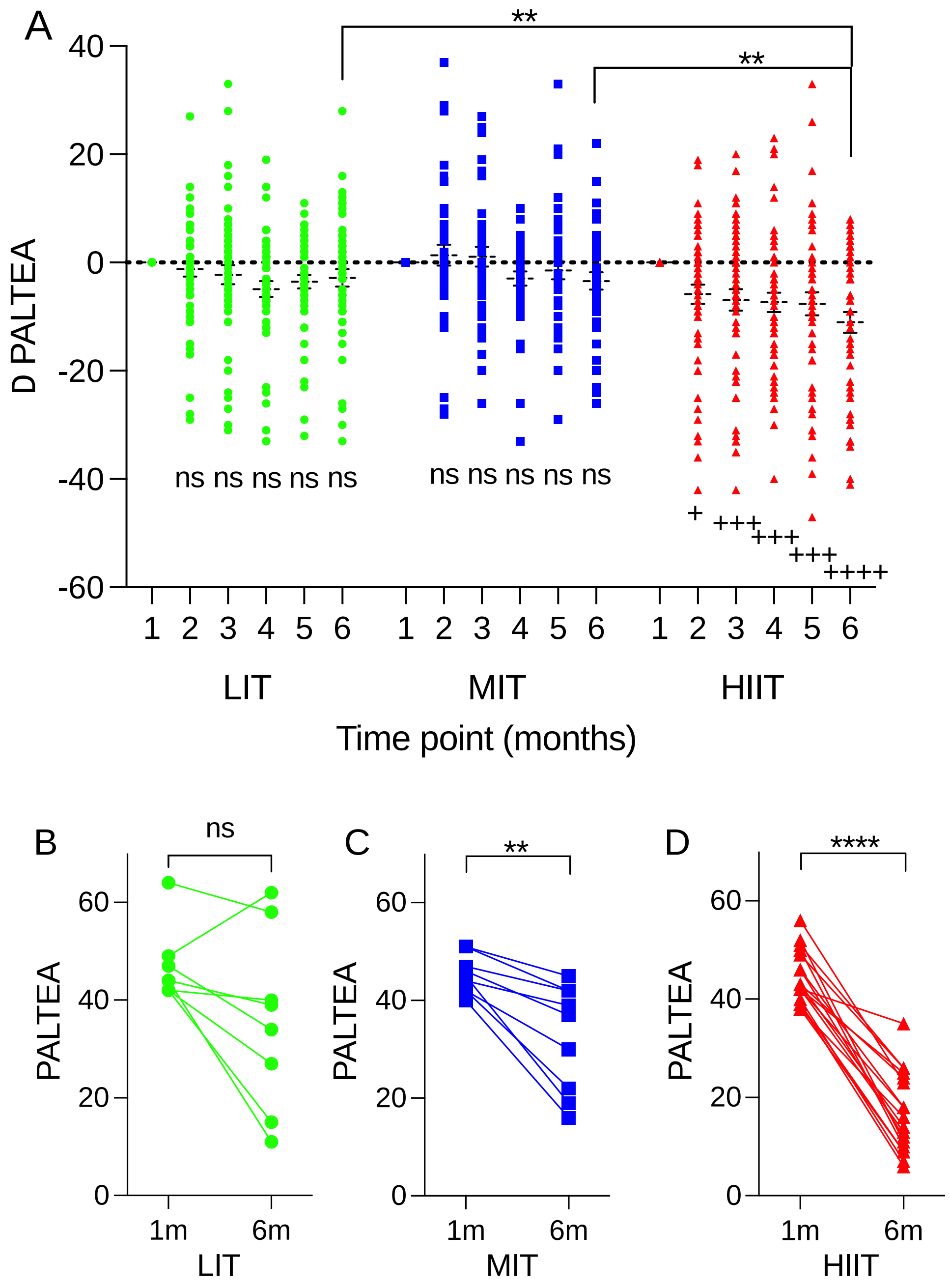

Supplementary Figure 2. **Distribution of PALTEA performance over the exercise intervention and only initial poor PALTEA performers from the MIT and HIIT groups improved at the completion of the exercise intervention.** (**A**) Raincloud plot showing distribution of performance of each participant over the 6-month testing period. This is the same data as Fig 2A. At the 6-month timepoint the HIIT group significantly outperformed the LIT group (mean±SE; two-way RM-ANOVA [time x exercise intensity effect F (10,738) = 2.272, p= 0.012], with Bonferroni post-hoc tests, p= 0.004 and p= 0.008 respectively) + p< 0.05, +++ p< 0.001, ++++ p< 0.0001 within group differences compared to 1-month. (**B**) LIT participants who were above the mean+1SD at the initiation of exercise did not improve during the intervention (paired t-test, t=2.141, df= 7, p= ns). (**C**) There was significant improvement in PALTEA performance for the MIT participants (paired t-test, t=4.668, df= 8, p= 0.002 and, (**D**) HIIT participants (paired t-test, t=14.17, df= 17, p< 0.0001) who started the intervention above the mean+1SD level. ns= non-significant, ** p< 0.01 and **** p< 0.0001).

© 2024. Blackmore DG et al. Published online at http://www.aginganddisease.org/EN/10.14336/AD.2024.042

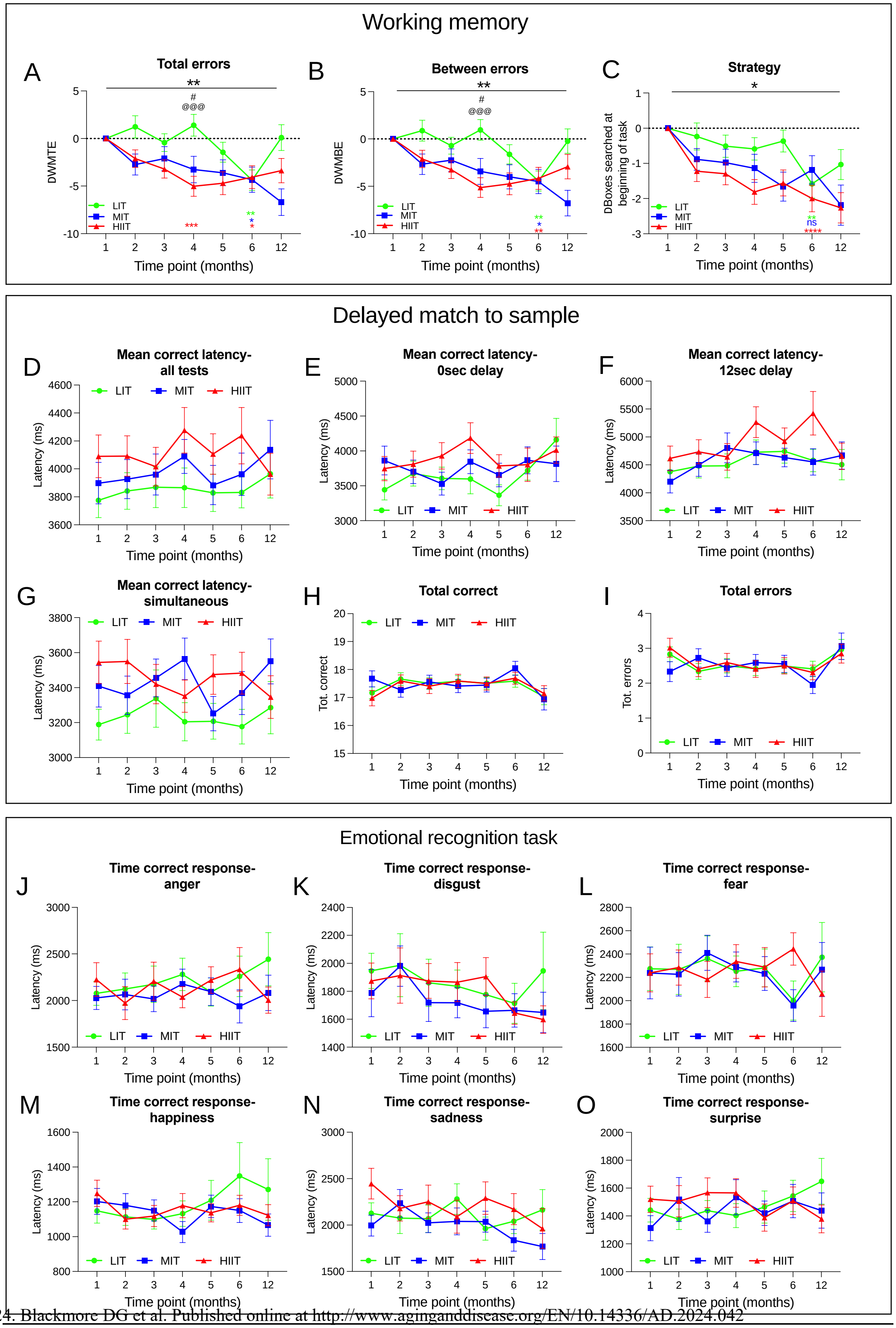

© 2024. Blackmore DG et al. Published online at http://www.aginganddisease.org/EN/10.14336/AD.2024.042

Supplementary Figure 3. Working memory, visual working memory and emotional memory were not significantly different between groups at the end of the exercise intervention. (**A**) Working memory total errors (WMTE) showed significant improvement for all groups during testing with all groups performing significantly better at completion of the exercise trial (two-way RM-ANOVA [time x exercise intensity effect F (12,821) = 2.525, *p*= 0.002]). Both (**B**) between errors (WMBE) and (**C**) strategy employed also showed a significant improvement during the testing period but showed no difference between groups at completion of the exercise intervention (two-way RM-ANOVA [time x exercise intensity effect F (12,821) = 2.43, *p*= 0.004] and two-way RM-ANOVA [time x exercise intensity effect F (12,821) = 1.79, *p*= 0.04] respectively). Visual working memory, as tested by delayed match to sample (DMS), showed no differences over time or between any groups over the length of the trial as measured by (**D**) mean latency for correct responses where all delays are included (DMSML), (**E**) mean latency for correct responses with no delay (DMSML0), (**F**) mean latency for correct responses with 12 second delay (DMSML12), (**G**) mean latency for correct responses with stimulus presented simultaneously (DMSMLS), (**H**) total number of correct responses (DMSTC) or (**I**) total number of errors. The time to correctly identify different facial emotions was not significantly altered between groups and did not change during the exercise trial. This included the mean time to correctly identify (**J**) anger, (**K**) disgust (**L**) fear, (**M**) happiness, (**N**) sadness and (**O**) surprise (mean±SE; mixed effects model with post-hoc tests). Within group statistical comparisons relative to baseline are represented by significance indicators of the same colour whereas black significance indicators represent differences between groups. "#" represents significance between LIT and MIT whereas "@" represents differences between LIT and HIIT. *p< 0.05, **p< 0.01, ***p< 0.001, ****p< 0.0001, #p< 0.05, @@@p< 0.001.

© 2024. Blackmore DG et al. Published online at http://www.aginganddisease.org/EN/10.14336/AD.2024.042

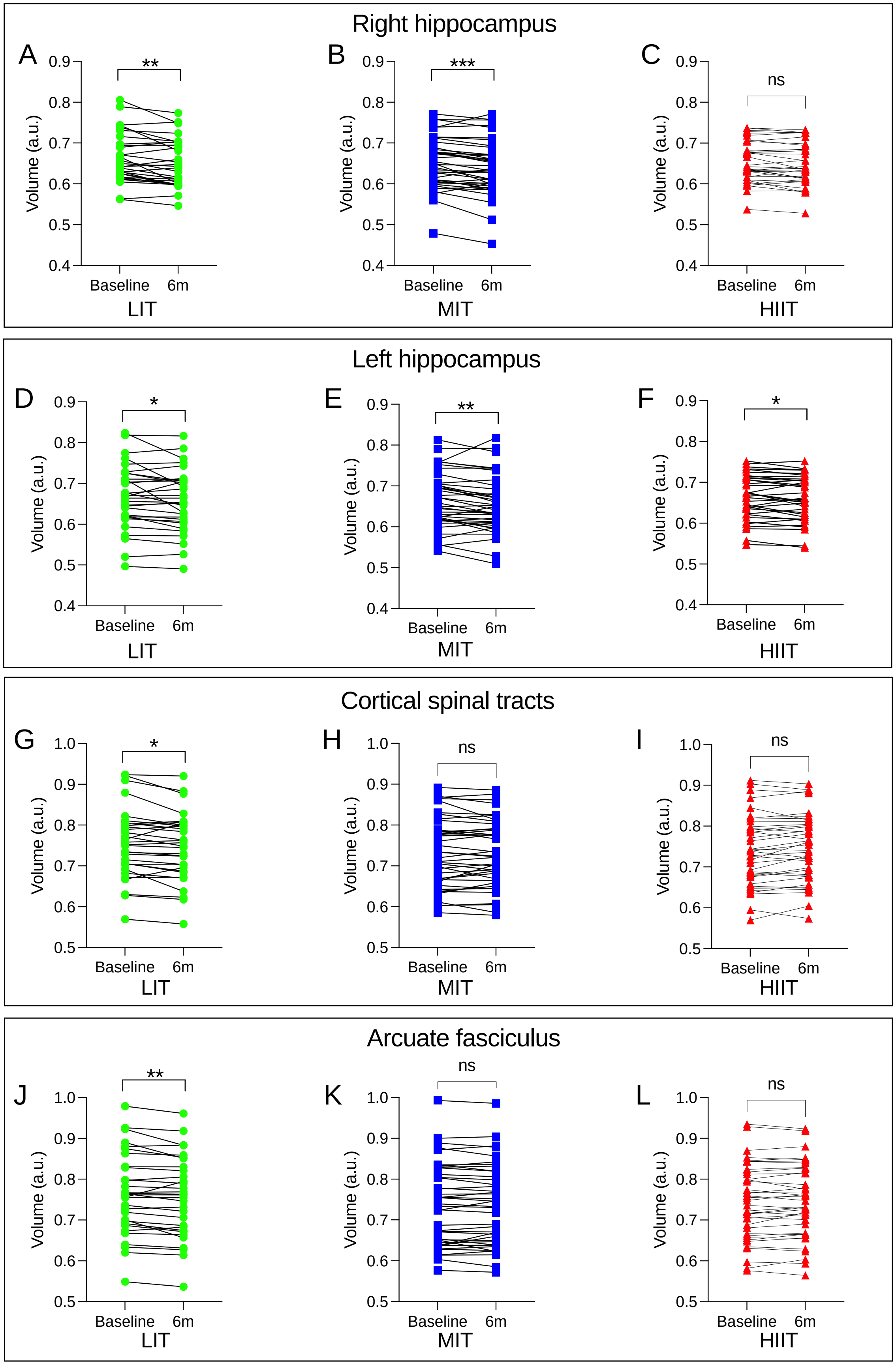

© 2024. Blackmore DG et al. Published online at http://www.aginganddisease.org/EN/10.14336/AD.2024.042

Supplementary Figure 4. HIIT reduces volumetric loss within key regions of the brain. (**A-C**) Baseline and 6-month right side hippocampal volumes were compared. Both the LIT and MIT groups showed a significant decrease in volume whereas the HIIT group remained stable (paired t-tests; LIT t= 3.6, df= 30, p= 0.0013, MIT t=3.7, df= 39, p= 0.0007, HIIT t=1.8, df= 34, p= 0.08). (**D-F**) All groups showed a significant decrease in volume for the left-hand side hippocampus (paired t-tests; LIT t= 2.1, df= 30, p= 0.04, MIT t=2.8, df= 39, p= 0.007, HIIT t=2.3, df= 34, p= 0.03). (**G-I**) The LIT group showed a significant decrease in white matter tracts associated with the cortical spinal tract (paired t-tests, t= 2.7, df= 30, p= 0.016). The MIT and HIIT groups remained stable (paired t-tests; MIT t= 0.41, df= 39, p= 0.68, HIIT t=1.78, df= 34, p= 0.09). (**J-L**) Only the LIT group showed a significant decrease in arcuate fasciculus volume while both the MIT and HIIT groups remained stable (paired t-test; LIT t= 3.0, df= 30, p= 0.005, MIT t=0.62, df= 39, p= 0.54, HIIT t=1.0, df= 34, p= 0.31). * p< 0.05, ** p< 0.01, and *** p< 0.001.

© 2024. Blackmore DG et al. Published online at http://www.aginganddisease.org/EN/10.14336/AD.2024.042

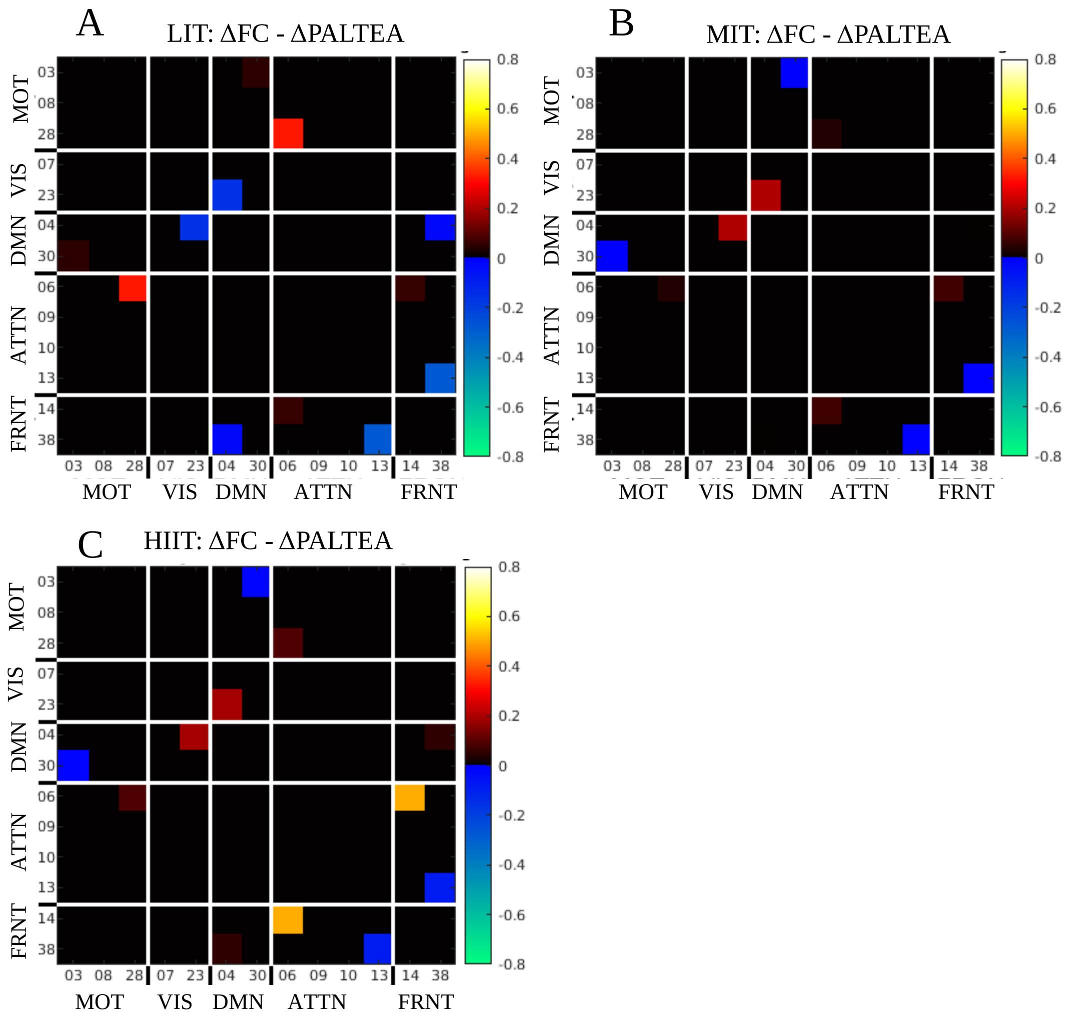

Supplementary Figure 5. **Exercise intensity-dependent changes correlating to cognitive function are observed between specific FC regions.** The change in FC from 0-month to 6-month was correlated to the change in PALTEA performance for each exercise group. Hot colours represent a positive Z score interaction, with cool colours representing a negative Z score. (**A**) The LIT group showed significant decreases in interactions between two network groups: DMN04-VIS23 and FRNT38-ATTN13. An increase was also observed between the ATTN06-MOT28 networks. (**B**) The MIT group showed a single increase in FC between DMN04-VIS23. (**C**) The HIIT group showed three positive interactions in FC between the networks FRNT14-ATTN06, DMN04-VIS23 and ATTN06-MOT28. Changes were measured as Z scores, with changes >±0.1 considered significant.

© 2024. Blackmore DG et al. Published online at http://www.aginganddisease.org/EN/10.14336/AD.2024.042

SUPPLEMENTARY DATA

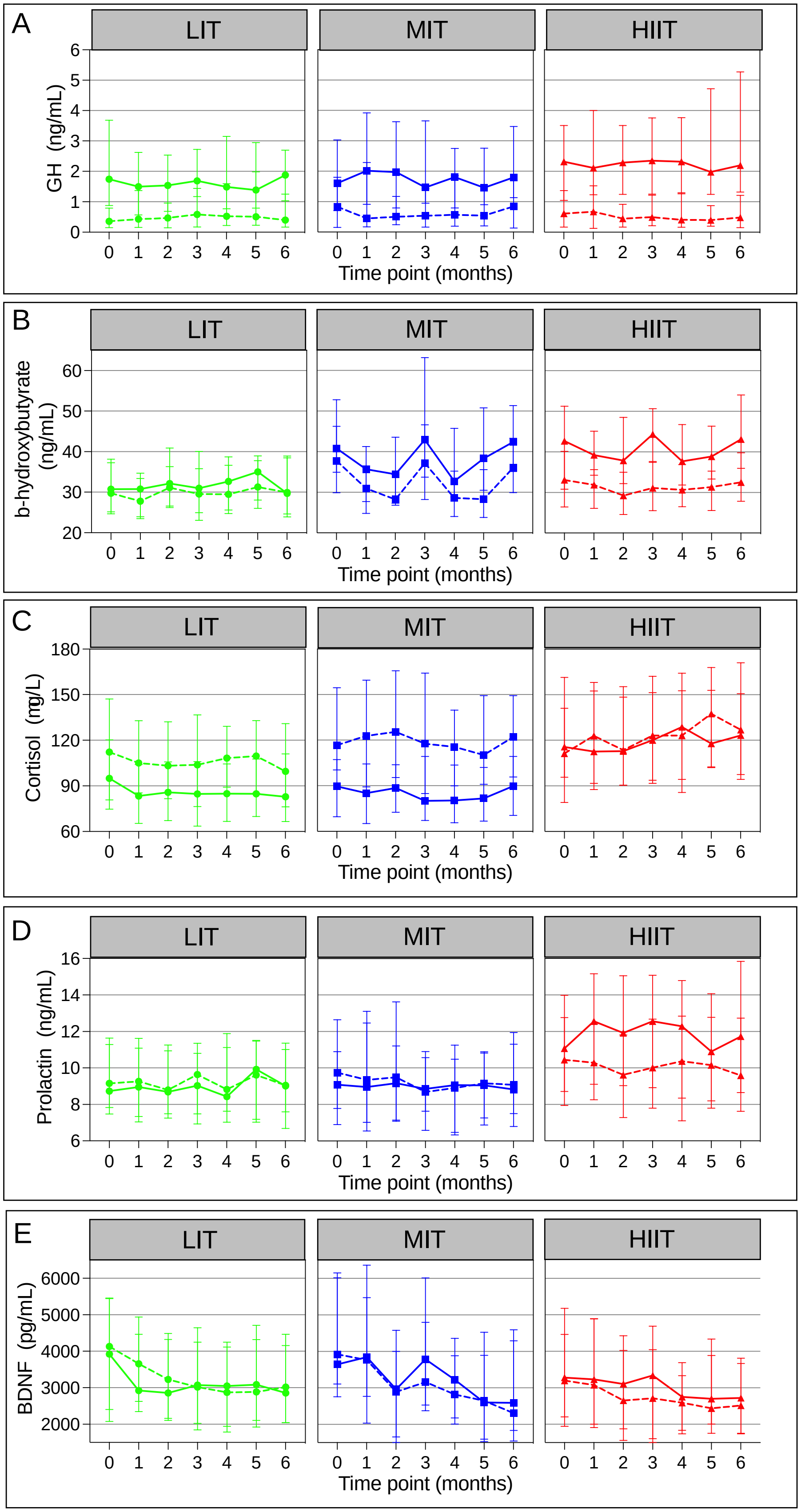

© 2024. Blackmore DG et al. Published online at http://www.aginganddisease.org/EN/10.14336/AD.2024.042

Supplementary Figure 6. Biochemical analysis of circulating biomarkers reveals differences in pre- and post-exercise values dependent upon the marker and exercise intensity. Pre- and post-exercise blood samples (dotted and solid lines respectively) were taken monthly, and several potential markers were examined. (**A**) GH levels increased following exercise for each intensity group (two-way RM-ANOVAs; LIT [exercise effect $F(1,102) = 22.24$, $p < 0.0001$], MIT [exercise effect $F(1,88) = 17.51$, $p < 0.0001$], HIIT [exercise effect $F(1,106) = 41.84$, $p < 0.0001$]). There was no significant change in either longitudinal pre-exercise levels or the amount of GH released post-exercise during the intervention for any of the exercise groups. (**B**) β-hydroxybutyrate showed higher post-exercise values for the MIT and HIIT groups (two-way RM-ANOVAs; LIT [exercise effect $F(1,98) = 0.7943$, $p = 0.375$], MIT [exercise effect $F(1,88) = 16.99$, $p < 0.0001$], HIIT [exercise effect $F(1,104) = 40.56$, $p < 0.0001$]). (**C**) Both the LIT and MIT groups had lower post-exercise cortisol levels whereas there was no change in the HIIT group (two-way RM-ANOVAs; LIT [exercise effect $F(1,102) = 19.07$, $p < 0.0001$], MIT [exercise effect $F(1,88) = 28.61$, $p < 0.0001$], HIIT [exercise effect $F(1,106) = 0.0826$, $p = 0.774$]). (**D**) Only the HIIT group showed an increase in prolactin during the exercise intervention (two-way RM-ANOVA; HIIT [exercise effect $F(1,106) = 8.81$, $p = 0.0037$]). (**E**) Over the 6-month exercise period all groups showed a general decrease in BDNF levels. The HIIT group showed higher monthly post-exercise BDNF levels; however, this did not reach significance. All graphs are represented as median±IQR.

© 2024. Blackmore DG et al. Published online at http://www.aginganddisease.org/EN/10.14336/AD.2024.042

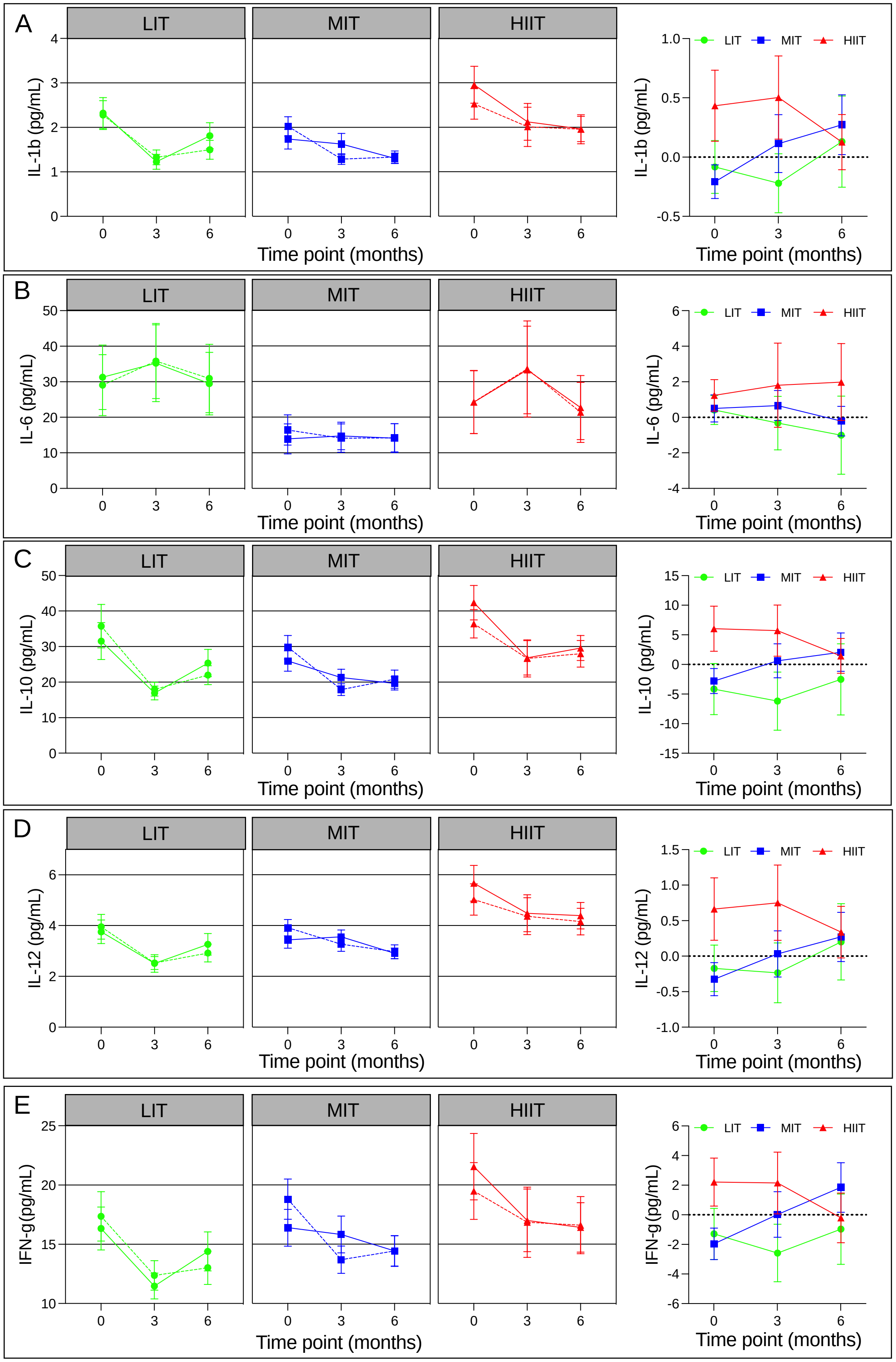

© 2024. Blackmore DG et al. Published online at http://www.aginganddisease.org/EN/10.14336/AD.2024.042

Supplementary Figure 7. There was no significant change in inflammation markers during the exercise intervention. The Pre- and post-exercise blood samples (dotted and solid lines respectively) at baseline, 3 months and 6 months were analysed for five different inflammation makers, with cumulative deltas also calculated. (**A**) Pre- exercise IL-1β levels were not significantly altered during the exercise intervention for any group. There was no significant difference in cumulative IL-1β over time for any group. (**B**) There was no significant change in IL-6 pre- and post-exercise levels or the cumulative level of IL-6 over time. (**C**) IL-10 showed no significant change during the exercise intervention. (**D**) IL-12 remained stable during the exercise intervention. (**E**) IFN- γ was not altered for pre- or post-exercise levels for any group, nor was there a significant difference between groups for cumulative levels (all data are mean±SE; two-way RM-ANOVA with post hoc tests).

© 2024. Blackmore DG et al. Published online at http://www.aginganddisease.org/EN/10.14336/AD.2024.042

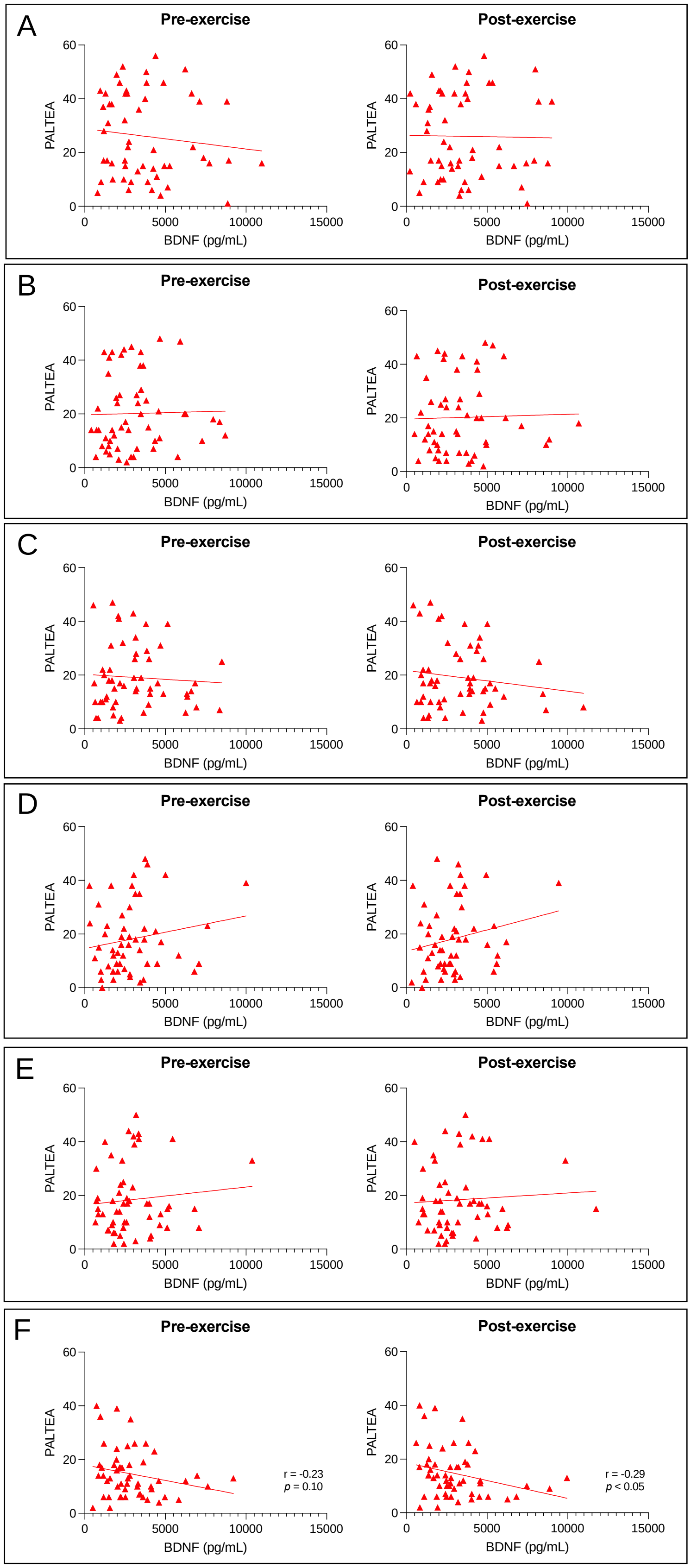

© 2024. Blackmore DG et al. Published online at http://www.aginganddisease.org/EN/10.14336/AD.2024.042

Supplementary Figure 8. There was no significant interaction between pre- and post-exercise concentration of BDNF and PALTEA performance. The concentration of BDNF was calculated pre- and post-exercise monthly during the exercise intervention. No correlation between pre- or post-exercise BDNF levels and PALTEA performance was observed at the 1-month (**A**), 2-month (**B**), 3-month (**C**), 4-month (**D**) or 5-month (**E**) time point. (**F**) At the 6-month time point a higher concentration of post-exercise BDNF correlated to better PALTEA ability. Spearman correlations were used for analysis.

© 2024. Blackmore DG et al. Published online at http://www.aginganddisease.org/EN/10.14336/AD.2024.042

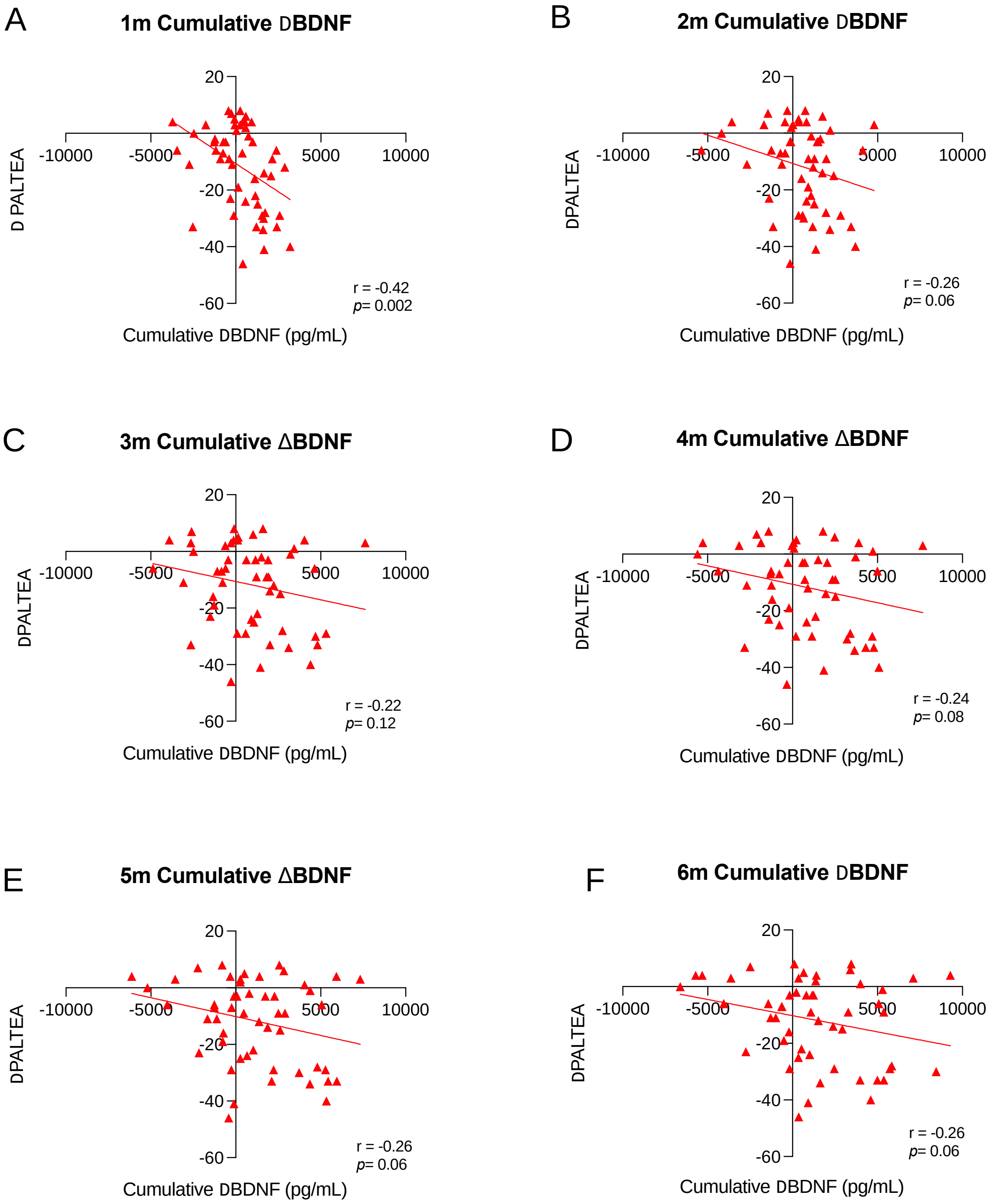

Supplementary Figure 9. The cumulative pre/post-exercise concentration (ΔBDNF) at each month showed a strong relationship to improved PALTEA performance in the HIIT group. The pre/post-exercise BDNF value (ΔBDNF) for each month was calculated for each participant. These values were then accumulated and compared to the change in PALTEA performance (ΔPALTEA). (**A**) The cumulative ΔBDNF concentration after 1-month of exercise showed a significant correlation with higher cumulative ΔBDNF correlating to better ΔPALTEA performance. (**B-F**) From 2 months of exercise and beyond there was a strong trend between higher cumulative ΔBDNF levels and improved ΔPALTEA performance. Spearman correlations were used for analysis.

© 2024. Blackmore DG et al. Published online at http://www.aginganddisease.org/EN/10.14336/AD.2024.042

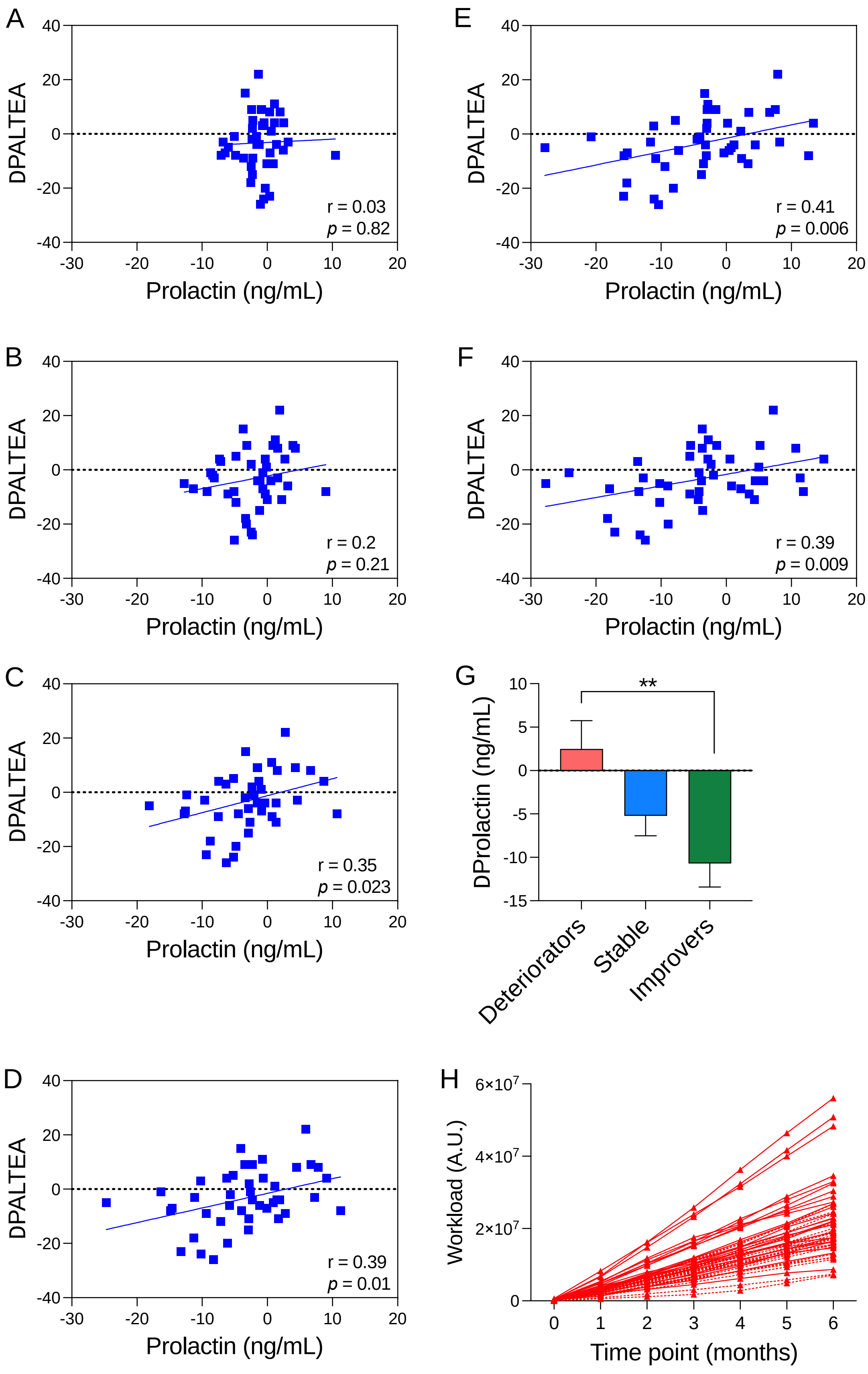

© 2024. Blackmore DG et al. Published online at http://www.aginganddisease.org/EN/10.14336/AD.2024.042

Supplementary Figure 10. Over the duration of the exercise intervention, lower prolactin levels correlate with improved PALTEA performance in MIT participants. As the exercise trial progressed, a correlation between lower levels of pre-post exercise-mediated prolactin levels and improved PALTEA (ΔPALTEA) appeared. (**A**) Initially, at baseline and after 1 month of exercise (**B**) no correlation were observed. (**C**) At 2 months and beyond, a lower prolactin level correlated with improved PALTEA performance (**D-F**). (**G**) Comparing total cumulative Δprolactin levels based on ΔPALTEA performance revealed a cognitive-dependent decrease in Δprolactin levels with highest improvers having less cumulative Δprolactin (mean±SEM; one-way ANOVA $[F (2,41) = 3.171]$, $p = 0.05$). (**H**) HIIT workload levels subdivided based on gender, with solid lines representing males and dotted lines representing females. Spearman correlations were used where appropriate. $^{**}p < 0.01$

© 2024. Blackmore DG et al. Published online at http://www.aginganddisease.org/EN/10.14336/AD.2024.042

SUPPLEMENTARY DATA

Circulating Biomarkers	Inflammation	Terminated early
Growth hormone	IL-10	Leptin
β-hydroxybutyrate	IL-6	IGFBP7
Cortisol	IL-1β	IL-8
Prolactin	IL-12p70	TNFα
BDNF	IFNγ	Insulin
IGF-1		Somatostatin

Supplementary Table 1. Analytes tested during the study. Multiple analytes were tested during the course of the study. The circulating biomarkers listed were included in further analysis. The cohort of inflammation-related biomarkers showed no change either during the intervention or between groups. Some analytes were not tested for every participant and are described as being terminated early.

Supplementary Appendices Contents:

S1 Appendix- ***Recruitment, health screening and inclusion criteria***
S2 Appendix- ***Aerobic stress tests, physiological monitoring, and workload calculations***
S3 Appendix- ***Electronic cognitive testing***
S4 Appendix- ***Magnetic resonance imaging (MRI)***
S5 Appendix- ***Blood biochemistry***
S6 Appendix- ***Physiological measures and functional fitness parameters***
S7 Appendix- ***Data storage, access and sharing***
S8 Appendix- ***Supplementary references***
S9 Appendix- **IC's for rsfMRI**

Supplementary Methods
Overview
Healthy older adults completed a comprehensive assessment of cognitive function, physiologic and functional fitness, physical activity, blood biochemistry and psychosocial measures prior to (0m), immediately following the completion of the exercise intervention (6m) and 6 months after the completion of their respective exercise intervention (12m). A subset of participants also conducted follow up physiological and cognitive testing at 6-month intervals up to 60 months. Participants were randomly allocated into either a low (LIT), moderate (MIT) or high intensity interval (HIIT) exercise training groups. Participants completed 3 supervised exercise sessions per week for 6 months for a total of 72 sessions. Multimodal MRI was conducted to obtain total subregion volumes and resting state functional connectivity during MRI scanning three times: 0m, 6m and 12m. Venous blood samples were taken on a monthly basis immediately prior to exercise and immediately following the completion of an exercise session and are referred to as pre- and post-exercise samples.

© 2024. Blackmore DG et al. Published online at http://www.aginganddisease.org/EN/10.14336/AD.2024.042

SUPPLEMENTARY DATA

S1 Appendix - Recruitment, health screening and inclusion criteria

Recruitment

In total, 194 healthy older adults aged 65-85 years were recruited via a multi-faceted advertising and recruitment process. Participants who met the inclusion and exclusion criteria and successfully completed all baseline measures, including an asymptomatic negative exercise stress test and MRI scan suitability questionnaire, were then enrolled in the study.

Screening

The screening process consisted of three stages:

1) Participants completed a 20-item online screening form to assess study eligibility, exercise readiness, medical and surgical history, and MRI eligibility. Information was sought to identify the presence of known disease, previous procedures or hospitalizations or planned procedures/ hospitalisations, medication use, significant mental illness, cerebrovascular and cardiovascular events, falls, fractures, head injuries, shrapnel or bullet wounds, and risk factors that may have excluded the participant from completing any of the planned measures. Participants submitted their completed form to the study database, and two qualified independent assessors determined study eligibility based on the answers provided.
2) Participants deemed eligible through the first stage of screening were provided with a General Practitioner (GP) Information statement and health check form and requested to attend an appointment with their GP to confirm their current medical status. The GP also provided consent for their patient to participate in the study.
3) The final stage of screening was completed in person during the initial visit. Medical history obtained during the online screening process was confirmed, and exercise readiness was assessed using the adult pre-exercise screening system.[1] Participants classified as 'high risk', or who had indicated the presence of three or more cardiovascular risk factors, were asked to obtain relevant information from their GP to enhance their safety prior to undertaking exercise.[2]

Inclusion criteria

Key inclusion criteria included: 1) be aged between 65-85 years old; 2) the ability and willingness to provide written informed consent; 3) the ability to communicate in English; 4) no history of stroke, heart or brain surgery, or brain trauma; 5) stratified as "not high risk of experiencing a cardiac event during exercise' according to pre-exercise screening; 6) no presently diagnosed mental illness or cognitive impairment, as per baseline Addenbrooke's cognitive examination-revised (ACE-R) scores; 7) not be medicated for dementia or psychiatric illnesses; 8) have a healthy body mass index (BMI) 18.5 – 30 $kg.m^{-2}$; and 9) be able and willing to commit to the duration of the exercise program (see Materials and Methods and Supp. Table 1).

Exclusion criteria

Participants were deemed ineligible if they met any of the following criteria: 1) younger than 65 years or older than 85 years of age; 2) illness or disability that precluded exercise or hindered completion of the study; 3) poorly controlled hypertension, cardiomyopathy, unstable angina, heart failure or severe arrhythmia, cancer, or chronic communicable infectious diseases; 4) current use of antipsychotics and/or antidepressants; 5) current or planned use of dehydroepiandrosterone, testosterone, transdermal oestrogens or other medications known to affect the growth hormone releasing hormone/growth hormone/insulin-like growth factor-1 axis; 6) currently diagnosed significant psychiatric illness, such as depression or schizophrenia, or dementia; 7) any contraindication for 7 Tesla MRI investigation, including recent joint replacements, stents, brain or heart surgery, bullet or shrapnel wounds and metal in the eye; 8) tobacco use, excessive alcohol intake (more than four alcoholic drinks per day), excessive caffeine intake (more than four cups of coffee per day); 9) excessive exercise involvement (more than twice the weekly recommendations for adults or >600 min moderate to vigorous exercise per week); 10) test results that indicated study participation was unsafe or; 11) participation in conflicting studies.

Randomization of participants

We randomly assigned participants to one of three arms using a centrally controlled computer-generated randomization list, generated by an independent staff member. The treatment was assigned using sealed envelopes based on order of recruitment and stratified for sex only. All investigators were blinded to the allocation order; however due to practicality, outcome assessors and exercise trainers were not blinded to the allocation treatment arm.

Withdrawal of participants

© 2024. Blackmore DG et al. Published online at http://www.aginganddisease.org/EN/10.14336/AD.2024.042

Participants were able to withdraw from the study at any time. Upon withdrawal and upon participant request, all data relating to that participant were removed from the study and not included in data analysis. The most common reasons for withdrawal were group allocation, time commitment and moving away from the area. The development of a medical condition classed as an exclusion criterion or serious adverse reaction to the intervention or test procedures was assessed on a case-by-case basis by an experienced investigator. 78% of participants (151/194) completed the 6-month exercise intervention. The reasons for withdrawal of the 43 participants are included in Figure 1.

S2 Appendix- Aerobic stress tests, physiological monitoring, and workload calculations

Cardiovascular fitness

Peak aerobic capacity (VO_{2peak}) and peak heart rate (HR) were determined via a maximal graded exercise test to volitional fatigue using automated indirect calorimetry and 12-lead ECG cardiac monitoring (COSMED). Participants first completed a familiarization test to educate them regarding the safety procedures of the treadmill, with the exercise test completed on the second visit. Participants were provided with a pre-exercise preparation checklist to minimize intra-participant variability and were asked to confirm their adherence to the pre-trial preparation by completing and signing the checklist. Participants completed a Bruce maximal graded exercise test on the treadmill,[3] whereby walking speed and grade were increased every 3 min until the participant wished to stop or test termination criteria were achieved. HR was continuously monitored via 12-Lead ECG, with rating of perceived exertion (RPE; Borg 6-20 scale Borg)[4] and blood pressure (BP) recorded at the end of every stage. Expired air was continuously analysed via indirect calorimetry for fractional expired oxygen and carbon dioxide, to calculate VO_{2peak}, and VO_2/kg to assess physiological fitness.[5] Following test termination, recovery was monitored for 5 to 10 min, with HR and BP being monitored until both returned to pre-exercising values.

Attendance

All data for training sessions were recorded in an individual participant training diary. Attendance was calculated as the number of sessions attended divided by the total number of sessions available to attend (n=72).

Low-intensity exercise (control) condition (LIT)

Each session included 5-8 stretching, balance, range of motion and relaxation tasks, with a 10 min warm-up, 30 min class, and 5 min cool-down. HR and RPE was maintained in the American College of Sports Medicine 'light exercise' category[2] to ensure a negligible cardiometabolic effect. Exercises were modified regularly to minimize participant drop-out. The sessions were run in a different location to the aerobic exercise training.

Moderate intensity exercise condition (MIT)

Each session consisted of 45 min continuous treadmill walking exercise at 60-75% of peak HR, with a 10 min warm-up whereby participants increased the intensity of their exercise to reach the target HR, a 30 min session, and a 5 min cool-down. This exercise intervention was based on the American Heart Association and American College of Sports Medicine guidelines for aerobic physical activity.[6] Participants were required to reach their target HR at the end of the warm-up and were instructed to maintain this HR for the duration of the session.

High intensity interval training condition (HIIT)

Following a 10 min warm-up, participants completed four, 4 min working periods at 85-95% of peak HR interspersed by three 3 min active recovery intervals at 60-70% peak HR, followed by a 5 min cool-down, totalling 40 min of treadmill exercise as described elsewhere.[7-10] Participants were required to reach their target HR within the first 2 min of the interval and maintain this HR until the end of the 4 min interval.

Heart rate recordings

HR for LIT and MIT participants was recorded once during pre-warm up, once during the 10-min warm-up interval and then once every 5 min during the exercise session proper. A final HR was then taken during the 5 min cool-down interval. HR for HIIT participants was taken once during pre-warm up and again once during the 10 min warm-up interval. During the four, 4 min aerobic intervals HR was taken every minute. During the x3 3 min active recovery intervals the HR was recorded once every recovery interval. A final HR was also obtained during cool-down.

Workload calculations

The MIT and HIIT groups had the speed and grade of the treadmill recorded simultaneously with HR multiple times during each exercise session. These parameters allowed for the calculation of exercise workload. For both MIT and HIIT,

© 2024. Blackmore DG et al. Published online at http://www.aginganddisease.org/EN/10.14336/AD.2024.042

participants were free to alter the treadmill speed and/or treadmill incline to maintain required HR. Therefore, the workload was calculated differently depending on whether the treadmill had an incline. The equations are provided below:

When grade (θ) > 0:

$$W = mgsin(\theta)(^{v}/_{3.6})60 + \left(0.5mcos(\theta)(^{v}/_{3.6})\right)^2,$$

where m = body mass, g = acceleration due to gravity.

When grade (θ) = 0:

i/ for males:

$$W = 0.5m(^{v}/_{3.6})^2 + mg\frac{(^{v}/_{3.6})60}{0.415h\times(4(0.56h - sin(79.32)\times 0.56h))},$$

where m = body mass, g = acceleration due to gravity and h = height

ii/ for females:

$$W = 0.5m(^{v}/_{3.6})^2 + mg\frac{(^{v}/_{3.6})60}{0.413h\times(4(0.56h - sin(79.32)\times 0.56h))},$$

where m = body mass, g = acceleration due to gravity and h = height

Missing values for grade or speed were imputed using the average between previous and following session for the same stage.

Safety: An adverse event was defined as any untoward medical occurrence in a participant undergoing an intervention. The reporting period for an adverse event was defined as the period from initiation of the study treatment to the end of the 12-month follow-up. All adverse events were recorded using authorized incident report forms and were reviewed by Occupational Health and Safety staff, project staff and the heads of organizational units. The principal investigator registered all adverse events with the ethical review committee within 24 h of the incident. Adverse events were graded by the principal investigator as mild (causing no limitation of usual activities), moderate (causing some limitation of usual activities), or severe (causing inability to carry out usual activities). No adverse events resulted in hospitalization. Pre-existing conditions present at the start of the study were recorded as an adverse event if the frequency, intensity, or character of the condition worsened during the study period. During the study there were a total of 2 adverse events with one in the moderate adverse event range (cardiac discomfort that occurred at home). The participant had reported other cardiac risk factors and was monitored for high BP. One participant fell into the severe adverse event range (injured knee requiring physiotherapy).

S3 Appendix- Electronic cognitive testing

The Cambridge Automated Neuropsychological Test Battery (CANTAB; Cambridge Cognition Ltd.) was used. These cognitive tests were self-administered using an iPad touch screen interface and took approximately 25 min to complete during their visit to the clinic. Outcomes were automatically recorded in the application for later retrieval by study staff.

Paired Associated Learning (PAL): PAL consisted of multiple trials of increasing difficulty designed to test object-location memory. Boxes were displayed around the border of the screen and were "opened" automatically in a randomized order. One or more of these boxes contained a complex pattern. The patterns were then displayed in the middle of the screen, one at a time, and the participant had to select the box in which the pattern was originally located. The test increased in difficulty, moving from 2 boxes to 8. Participants had to complete the task with no errors before the task moved onto the next, more difficult task. If the participant failed four times at any level the test concluded. PALTEA provides a standardized score for each participant and is more accurate than reporting total errors (PALTE). We therefore focused on this readout for changes in cognitive function within this study. The higher the PALTEA score, the more poorly a participant performed. Initial (0m) PAL tests were used as familiarization with the subsequent 1-month cognitive tests used as baseline to measure the change in learning ability during the exercise intervention. As we observed no difference in PALTEA performance between the three exercise groups at 1 month of cognitive testing (one-way ANOVA $F(2, 150) = 2{\cdot}385$, $p = 0{\cdot}096$), the change in PALTEA performance (Δ PALTEA) for all groups is reported relative to performance at this timepoint and was used to measure changes in cognitive performance. Importantly, as we were examining the change in total adjusted errors, a reduction in this number represented an improvement in cognitive performance.

© 2024. Blackmore DG et al. Published online at http://www.aginganddisease.org/EN/10.14336/AD.2024.042

Delayed Matching to Sample (DMS): A complex abstract pattern was displayed on the screen followed by four similar patterns. The participant had to select the exact matching pattern to the sample. The delay following sample presentation was either 0, 4 or 12 seconds. We compared the latency to correctly identify the matching abstract pattern with all latencies included, following no delay, following 12 seconds delay as well as total correct and total errors made.

Working Memory (WM): Depending upon difficulty, boxes ranging from 4-8 were distributed around the screen. Through a process of elimination, the participant had to select the boxes to find a coloured token which filled a column in the right-hand side of the screen. During each trial, the participant had to remember which boxes had contained a token and not select those boxes again. The total errors (WMTE), between trial errors (WMBE) and the strategy used to open boxes were all compared. The change in performance was calculated by subtracting the initial WM score from subsequent WM scores for each of these parameters.

Emotional Recognition Task (ERT): This task examined the ability to identify one of six basic emotions in facial expressions. Faces displaying an emotion of either Anger, Disgust, Fear, Happiness, Sadness or Surprise were displayed for 200 ms before being covered. The participant then had to select which emotion was displayed. We compared the time required to correctly identify each of these emotions.

S4 Appendix- Magnetic resonance imaging (MRI)

To compare the effect of the different exercise paradigms on hippocampal volume we conducted longitudinal 7T high resolution multimodal MRI scans on a subpopulation of participants. Multimodal MRI scans were obtained using an ultra-high field 7T MRI scanner (Siemens) prior to exercise (0-month), immediately following completion of the exercise intervention (6-month) and 6 months after the completion of the exercise intervention (12-month). The MRI scan consisted of resting-state functional (rsfMRI), and structural MRI (sMRI) and functional MRI with task (not reported here). It took approximately 55 min to complete all imaging components. The longitudinal approach allowed specific volumetric and connectivity changes to be calculated for each participant.

Structural MRI analysis: Structural scans took 23 min to complete. High resolution T1-weighted (T1w) images with MP2RAGE sequence (whole brain; 0·75 mm isotropic voxel size) were used, with multi-slice 2D T2-weighted TSE acquired (three repetitions; slice thickness 0·8 mm, 72 slices; in-plane resolution 0·4 mm x 0·4 mm). We modified an established deformation-based framework pipeline[11] to examine whole brain volumetric changes between exercise groups in a data-driven and unbiased fashion to measure volume changes on a voxel-wise or region-of-interest (ROI) basis. Briefly, the procedure was as follows: the T1w-MP2RAGE image was segmented into white matter, grey matter (GM), cerebro-spinal fluid (CSF), skull, and other tissue probabilistic tissue maps using SPM12 (https://www.fil.ion.ucl.ac.uk/spm/software/spm12/). The WM, GM, and CSF maps were used for an iterative tissue-specific nonlinear image registration and template creation (using the default SPM's Diffeomorphic Anatomical Registration Through Exponentiated Lie Algebra [DARTEL] toolbox settings). For the segment MP2RAGE to WM, GM, CSF tissue probability maps (where each voxel has a value between 0 and 1; the value corresponds to the probability that the specific voxel is GM/WM/CSF, according to the segmentation algorithm). For the WM, tissue had probabilistic maps across all participants registered to a starting WM template, averaged and then used to create a new template. This was repeated, each with finer registration. This same procedure was also conducted for the GM and CSF maps. The generated maps were then warped to the MNI152 template space[12] and modulated as "Preserve Amount" to create smooth and modulated tissue probabilistic maps in the common MNI152 space. Modulation here used the “Preserve Amount” setting which allowed for local tissue volume comparison across different participants, groups, and time points. This approach allowed for a simpler contrast and value range to be acquire across the three tissues during registration.

Resting state functional MRI: We also conducted rsfMRI scans as the effect of HIIT on network connectivity has yet to be examined in the elderly. Images were acquired using the 7T Siemens scanner equipped with a 32-channel head coil. The acquisition of rsf data was achieved using a whole-brain $T2^*$-weighted echo-planar image (EPI) sequence (48 interleaved slices, repetition time (TR) = 655 ms, echo time (TE) = 25 ms, flip angle = 60°, field of view (FOV) = 190 mm, in-plane resolution= 2 x 2 x 2.2 mm resolution, multiband accelerator factor = 4). This portion of the scan took 5 min and participants were asked to close their eyes. Decomposition analysis examined 65 independent components (ICs) of scans from participants at baseline and at 6 months. From this, 13 functional ICs were identified based on the criteria for hand classification of ICA components[13] and group ICA-based functional connectivity architecture.[14] These included the: motor (MOT), visual (VIS), default-mode (DMN), attentional (ATTN), and frontal (FRNT) networks (Supp. Fig. 4).

© 2024. Blackmore DG et al. Published online at http://www.aginganddisease.org/EN/10.14336/AD.2024.042

Quality control: For images to be included in analysis the images had to meet the criteria; no obvious motion artefacts, signal dropouts, other artefacts, or other miscellaneous imaging errors (e.g. erroneous placements of the FOV) on the T1w structural image. If these criteria were met, the image was used for processing and analysis. For the quality of the processing pipeline: during the voxel-wise statistical analysis step, the normalised and modulated tissue probability maps from all participants and timepoints were stacked together in a 4D file and author X.V.T cycled through the different frames and check for inconsistencies and misregistration.

rsfMRI data were processed and analysed as follows. The rsfMRI data were motion-corrected using FSL's MCFLIRT then bandpass filtered between 0·008 – 0·8 Hz. The motion-corrected and bandpass-filtered data were averaged to create an EPI anatomical representation of the rsfMRI data. This EPI anatomical representation image was corrected for signal inhomogeneity using ANTs' (ANTs v·2·3·4 http://stnava.github.io/ANTs/) N4BiasFieldCorrection.[15] The EPI images were then used for an iterative image registration and template creation process to register the EPI images to an EPI study-specific template[16] using ANTSs[17] antsMultivarateTemplateConstruction2.sh. The EPI study-specific template was then registered to the MNI152 template. The warping fields obtained were used to warp the motion-corrected and bandpass-filtered rsfMRI data to a common space. Head motion parameters and other "noise" typically regressed out of the individual-level rsfMRI were not done so in this study for several reasons. First, as Bright et al. noted : "Simulated nuisance regressors, unrelated to the true data noise, also removed variance with network structure, indicating that any group of regressors that randomly sample variance may remove highly structured 'signal' as well as 'noise.'"[18]. Second, artefact-removal based on group-information-guided Independent Component Analysis (GIG-ICA) was shown to be inferior, if not superior to single-subject artefact removal[19]. Therefore, artefact removal at the individual-level with, e.g. motion and global signal regressors, were skipped for this data set and we relied on group Independent Component Analysis for separation of signal and noise at the group-level. The spatially normalized rsMRI data were then analysed using group-information-guided Independent Component Analysis (GIG-ICA)[20] as implemented in the Group ICA of fMRI Toolbox (GIFT).[21] GIG-ICA were performed between baseline and 6-month data of 38 HIIT, 40 MIT, and 30 LIT participants (218 data sets in total). The number of ICs was set to 65. The group-level ICs were hand-classified 13 functional ICs based on prior hypotheses and previous studies[13, 14]; these ICs belonged to the motor (MOT), visual (VIS), default-mode (DMN), attentional (ATTN), and frontal (FRNT) networks. The resulting spatial maps and IC-to-IC functional connectivity (FNC) were used for two-way repeated measures analysis of variance (ANOVA) for the group effect (HIIT vs. MIT vs. LIT), time effect (baseline vs. 6 months) and group x time interaction effects using the GIFT's MANCOVA toolbox.[14] Paired post-hoc t-tests were also conducted to examine the difference at 6 months compared to the baseline within each exercise intensity group. The statistical analysis results of rsfMRI analysis were corrected for multiple comparison using the false discovery rate (FDR) at Q value < 0·1. The IC-IC FNC was also extracted to allow for correlation analysis with PALTEA cognitive scores.

S5 Appendix- Blood biochemistry

Monthly blood samples were taken immediately pre- and post-exercise during the exercise intervention. This allowed for a longitudinal examination of changes for multiple circulating markers and correlation of these to cognitive ability. Previous studies have generally focused on a limited number of markers and typically only collected samples at the initiation and/or completion of the exercise trial, thereby limiting the information that could be obtained.[22, 23] In this study, a total of 17 circulating biomarkers were examined in relation to exercise-mediated changes and their possible correlation to changes in cognitive function. The full list of these is included in Supp. Table 2.

Blood sample collection: Venous blood samples (a total of 40 mL) were collected from the antecubital vein using a 21-gauge needle into prepared vacuum tubes (Sarstedt) containing either K3EDTA, micronized silica, lithium heparin or fluoride oxalate. Serum tubes (micronized silica) was allowed to clot at room temperature (RT), all other tubes were stored on ice. Two aliquots of whole blood were taken from the EDTA (plasma) and 2 from the lithium heparin prior to centrifuging. 30 min after blood samples were collected, samples were centrifuged at 1100 x g for 10 min at 4°C. Following centrifugation, 10 aliquots of serum and plasma (EDTA) supernatant, each at 350µl, were aliquoted into separate, colour-coded and barcoded tubes. The supernatant from the lithium heparin samples was divided into 2 aliquots, while the supernatant from the fluoride oxilate was separated into 4 aliquots.

Blood sample storage: The total number of aliquots (30) were then separated evenly into A and B boxes and stored in separate -80°C freezers until analysis. Aliquot tubes were colour coded and labelled with bar-coded,

© 2024. Blackmore DG et al. Published online at http://www.aginganddisease.org/EN/10.14336/AD.2024.042

- 80°C rated stickers, to identify participant ID, time point and type of sample including pre- or post-exercise sample. A barcode reader and electronic notebooks were used to record and track the location of each sample.

ELISAs: Growth hormone (GH), cortisol, prolactin and insulin were analysed simultaneously from a single serum sample using a Cobas e411 electro-chemilumescence immunoassay autoanalyser (Roche Diagnostics) and manufacturer recommended Elecsys assays. Manufacturer-supplied reagents were used, and instruments calibrated as per the manufacturer's instructions. The co-variances (CVs) for GH, prolactin, insulin and cortisol were 1·0%, 1·0 %, 1·0 % and 0·9 %, respectively. The BDNF ELISAs (R & D Systems; Cat # DY248) were conducted using a 1:25 dilution for plasma samples as per the manufacturer's instructions. IGF-1 ELISAs (R & D Systems; Cat # DG100B) were conducted using a 1:500 dilution for plasma samples. The Magpix high sensitivity cytokine panel (Millipore/ Merck; Cat # HCYTOMAG-60K) for IFN-γ, IL-1β, IL-6, IL-10 and IL-12 was conducted using plasma samples at a final dilution of 1:4. The ELISA for ß-hydroxybutyrate (BHB, Sigma-Aldrich; Cat # MAK041-1KT) was conducted using a 1:25 dilution using plasma samples as per the manufacturer's instructions.

Analyte analysis: The pre- and post-exercise concentration of each analyte was measured via ELISA as described above and longitudinally compared for each exercise group. For each participant we also calculated the difference in concentration between the pre- and post-exercise values for each analyte. We referred to this as the delta value (Δ). The monthly Δ values were then added together to obtain the cumulative delta value for each analyte of interest.

Appendix 6- Physiological measures and functional fitness parameters

General parameters: A large number of general parameters were collected during in-person visits, including height, weight, age and sex. Waist and hip circumference were recorded, as were resting systolic and diastolic BP and HR. Waist to hip ratio and body mass index were calculated from these readings and remeasured at the 3-month, 6-month and 12-month time points.

Global cognitive function- ACE-R: The Addenbrook's Cognitive Examination-Revised (ACE-R; with an inbuilt Mini Mental State Exam (MMSE)) is a routinely administered paper-based investigator-administered examination with set instructions.[24] This test was administered at baseline, 6-month and 12-month time points by trained individuals and was completed in approximately 15 min.

Functional fitness: Measures of functional fitness included hand grip strength (spring-loaded grip dynamometer; TTM, Tokyo, Japan),[25] and tandem, semi-tandem and single leg balance tasks,[26] following standardized testing protocols. These tests were administered at baseline, 6-month and 12-month time points.

Accelerometry analysis: Habitual physical activity was measured for 7 consecutive days using tri-axial accelerometry (Actigraph®) at baseline, 3-month, 6-month, 12-month and 42-month time points. Established cut points for counts per minute were used to measure time spent in sedentary, light, moderate and vigorous physical activity (minutes per day), and all results were validated against a 7-day physical activity diary and a wear-time diary.

Subjective physical activity measurement: Weekly physical leisure-time activity behaviours of participants were quantified using the Godin leisure-time exercise questionnaire. The Godin leisure-time physical activity questionnaire requires participants to recall during a typical 7-day week the frequency and duration of exercise completed at three separate intensities: mild, moderate, and strenuous intensity. These values were used to calculate a weekly leisure-time activity index based on summing the frequency of sessions completed multiplied by a weighting factor for each level of intensity.

Appendix 7-Data storage, access and sharing

In accordance with the Australian Privacy Principles 2014, the Australian Code of the Responsible Conduct of Research and the National Health Medical Research Council (NHMRC) National Guidelines for the Ethical Conduct of Human Research (2007), data were de-identified by referencing through a six-character participant code. DICOM images from MRI data were anonymized by only using the participant code. Digital data including DICOM images and scanned paperwork in pdf format were stored on an access-controlled dedicated shared network drive with high integrity permanent backup capability at The University of Queensland with access limited to authorized research and IT staff.

SUPPLEMENTARY DATA

Raw data files were generated via both automated and manual means in a range of formats. To provide a uniform interface for data management, an instance of XNAT (https://xnat.org), an open-source extensible DICOM repository,[27] was installed on a virtual machine within the internal Queensland Brain Institute (QBI) network, ensuring that access to the URL was only available via authorized access to the QBI network. In addition, authenticated login access was managed via XNAT itself. XNAT data were managed via a project-subject-experiment hierarchy. A customized series of plugins was developed to manage specified data fields for each experimental data set (https://github.com/QBI-Software/XNAT_modules/tree/master/opex-plugin). Data were then extracted from the raw data files (and in some cases, from several sources for the one experiment type) and uploaded directly to XNAT by a custom-built software application (OPEX Uploader) built in Python with a frontend In wxPython (https://www.wxpython.org/). The uploader was used for both spreadsheet and DICOM uploads, as well as providing CSV download. Patient DICOM images were stored in an XNAT database with custom modules for raw data from tests and a custom built upload software (https://github.com/QBI-Software/OPEXUploader). A custom reporting capability was also developed as a Python-based dashboard in Dash by Plotly (https://plot.ly/products/dash/) running over WSGI on an Apache server on the same XNAT VM. The source code and documentation for use are available on Github at https://github.com/QBI-Software/OPEXUploader licensed under the GNU General Public License v3·0. Data stored on the group share drive and in the XNAT database were available for access by authorized users only. Requests for collaborative shared access to data are required to go through appropriate authorization procedures before being granted. XNAT provided full auditing of user access to the XNAT database through its access logs. The host VM and shared data drive were fully backed up on a frequent schedule with replication at a geographically remote location in compliance with The University of Queensland's Research Management Policy (http://ppl.app.uq.edu.au/content/4.20.06-research-data-management).

Appendix 8- Supplementary References

1. Norton K, and Norton L (2011). Pre-exercise screening. Guide to the Australian adult pre-exercise screening system exercise and sports science Australia.
2. Thompson PD, Arena R, Riebe D, Pescatello LS, and American College of Sports M (2013). ACSM's new preparticipation health screening recommendations from ACSM's guidelines for exercise testing and prescription, ninth edition. Curr Sports Med Rep, 12: 215-7.
3. Bruce RA, Lovejoy FW, Jr., and et al. (1949). Normal respiratory and circulatory pathways of adaptation in exercise. J Clin Invest, 28: 1423-30.
4. Borg GA (1973). Perceived exertion: a note on "history" and methods. Med Sci Sports, 5: 90-3.
5. Meyer T, Davison RC, and Kindermann W (2005). Ambulatory gas exchange measurements- current status and future options. Int Sports Med, 26 Suppl 1: S19-27.
6. Garber CE, Blissmer B, Deschenes MR, Franklin BA, Lamonte MJ, Lee IM, et al. (2011). American college of sports medicine position stand. Quantity and quality of exercise for developing and maintaining cardiorespiratory, musculoskeletal, and neuromotor fitness in apparently healthy adults: guidance for prescribing exercise. Med Sci Sports Exerc, 43: 1334-59.
7. Moholdt T, Aamot IL, Granoien I, Gjerde L, Myklebust G, Walderhaug L, et al. (2012). Aerobic interval training increases peak oxygen uptake more than usual care exercise training in myocardial infarction patients: a randomized controlled study. Clin Rehabil, 26: 33-44.
8. Moholdt T, Madssen E, Rognmo O, and Aamot IL (2014). The higher the better? Interval training intensity in coronary heart disease. J Sci Med Sport, 17: 506-10.
9. Weston KS, Wisloff U, and Coombes JS (2014). High-intensity interval training in patients with lifestyle-induced cardiometabolic disease: a systematic review and meta-analysis. Br J Sports Med, 48: 1227-34.
10. Ramos JS, Dalleck LC, Tjonna AE, Beetham KS, and Coombes JS (2015). The impact of high-intensity interval training versus moderate-intensity continuous training on vascular function: a systematic review and meta-analysis. Sports Med, 45: 679-92.
11. Ashburner J (2007). A fast diffeomorphic image registration algorithm. Neuroimage, 38: 95-113.
12. Fonov V, Evans AC, Botteron K, Almli CR, McKinstry RC, Collins DL, et al. (2011). Unbiased average age-appropriate atlases for pediatric studies. Neuroimage, 54: 313-27.
13. Griffanti L, Douaud G, Bijsterbosch J, Evangelisti S, Alfaro-Almagro F, Glasser MF, et al. (2017). Hand classification of fMRI ICA noise components. Neuroimage, 154: 188-205.
14. Allen EA, Erhardt EB, Damaraju E, Gruner W, Segall JM, Silva RF, et al. (2011). A baseline for the multivariate comparison of resting-state networks. Front Syst Neurosci, 5: 2.
15. Tustison NJ, Avants BB, Cook PA, Zheng Y, Egan A, Yushkevich PA, et al. (2010). N4ITK: improved N3 bias correction. IEEE Trans Med Imaging, 29: 1310-20.

© 2024. Blackmore DG et al. Published online at http://www.aginganddisease.org/EN/10.14336/AD.2024.042

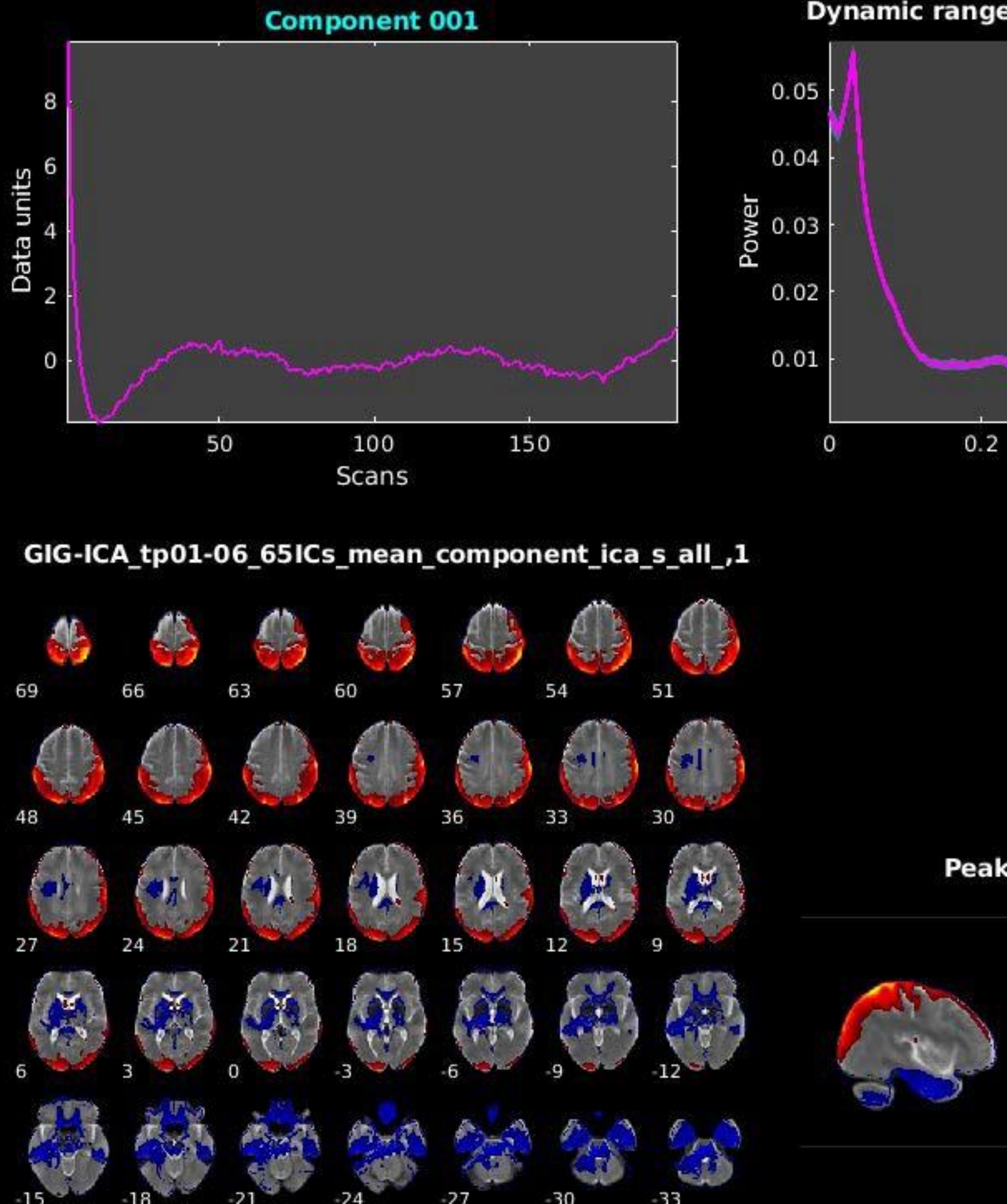

Component 001
Data units
Scans
Dynamic range
Power
GIG-ICA_tp01-06_65ICs_mean_component_ica_s_all_,1
Peak

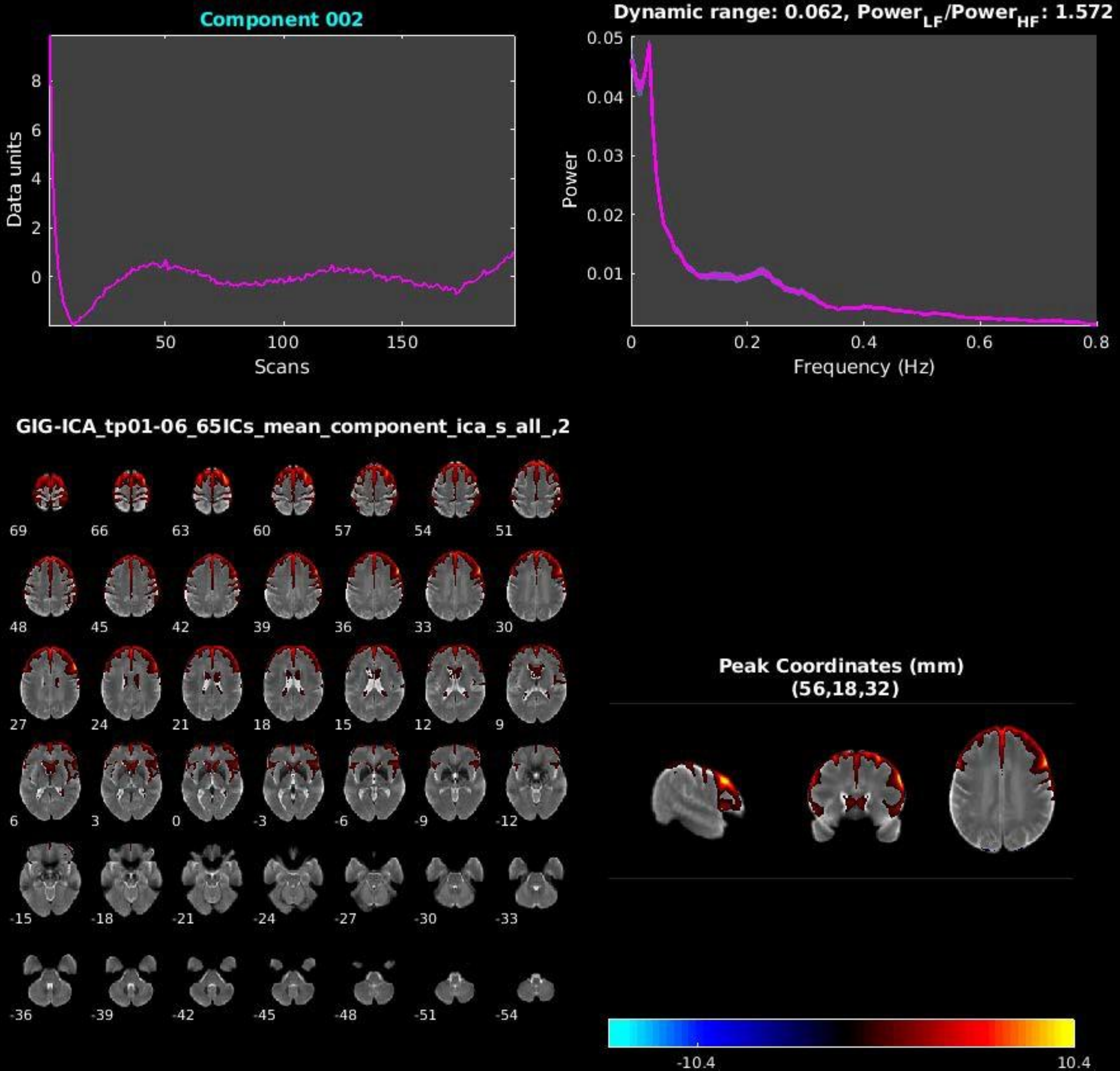
Component 002
Data units
Scans
0 2 4 6 8
50 100 150
Dynamic range: 0.062, $Power_{LF}/Power_{HF}$: 1.572
Power
Frequency (Hz)
0.01 0.02 0.03 0.04 0.05
0 0.2 0.4 0.6 0.8
GIG-ICA_tp01-06_65ICs_mean_component_ica_s_all_,2
69 66 63 60 57 54 51
48 45 42 39 36 33 30
27 24 21 18 15 12 9
6 3 0 -3 -6 -9 -12
-15 -18 -21 -24 -27 -30 -33
-36 -39 -42 -45 -48 -51 -54
Peak Coordinates (mm)
(56,18,32)
-10.4
10.4

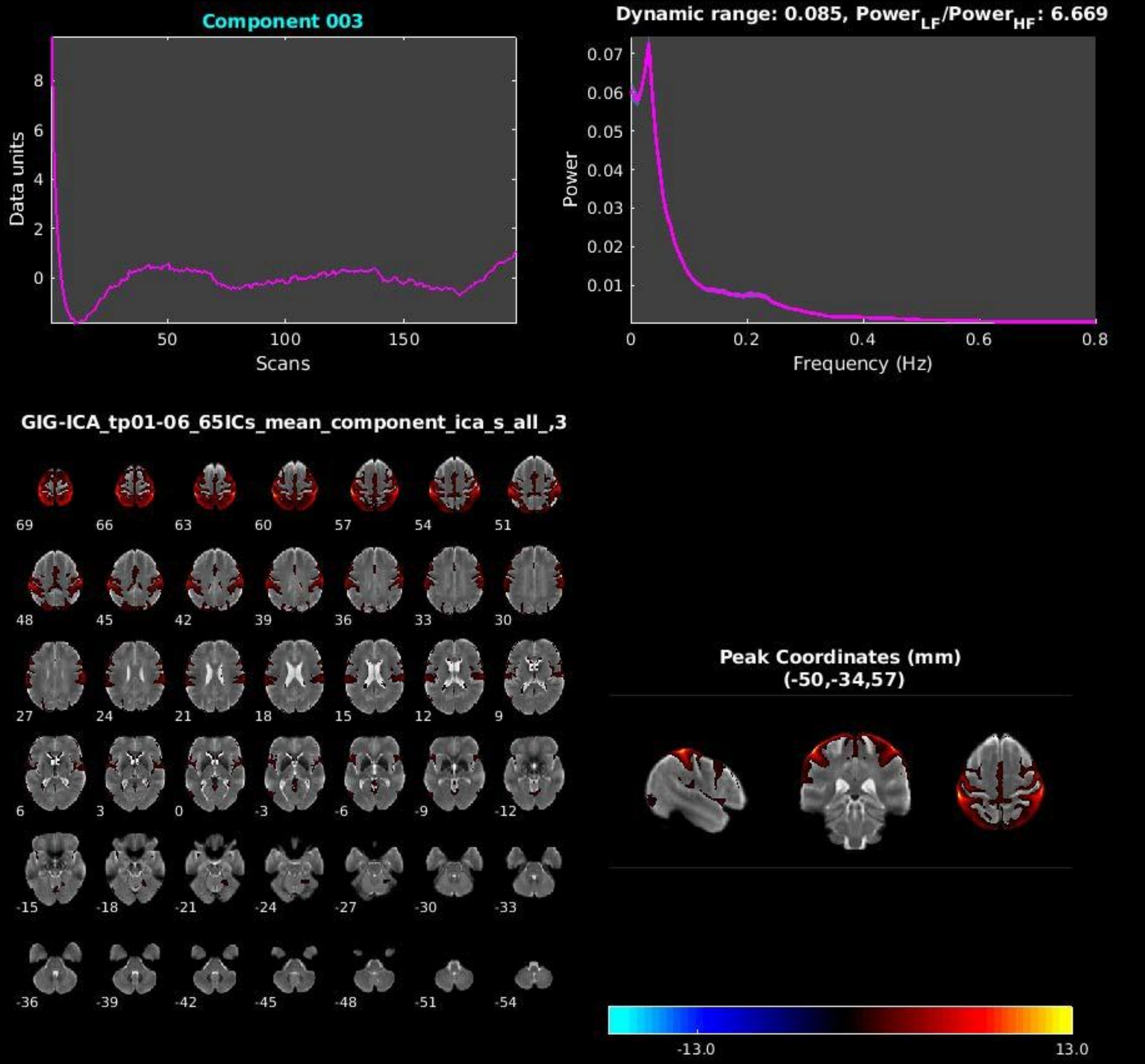

Component 003
Data units
Scans
Dynamic range: 0.085, Power$_{LF}$/Power$_{HF}$: 6.669
Power
Frequency (Hz)
GIG-ICA_tp01-06_65ICs_mean_component_ica_s_all_,3
69 66 63 60 57 54 51
48 45 42 39 36 33 30
27 24 21 18 15 12 9
6 3 0 -3 -6 -9 -12
-15 -18 -21 -24 -27 -30 -33
-36 -39 -42 -45 -48 -51 -54
Peak Coordinates (mm)
(-50,-34,57)
-13.0
13.0

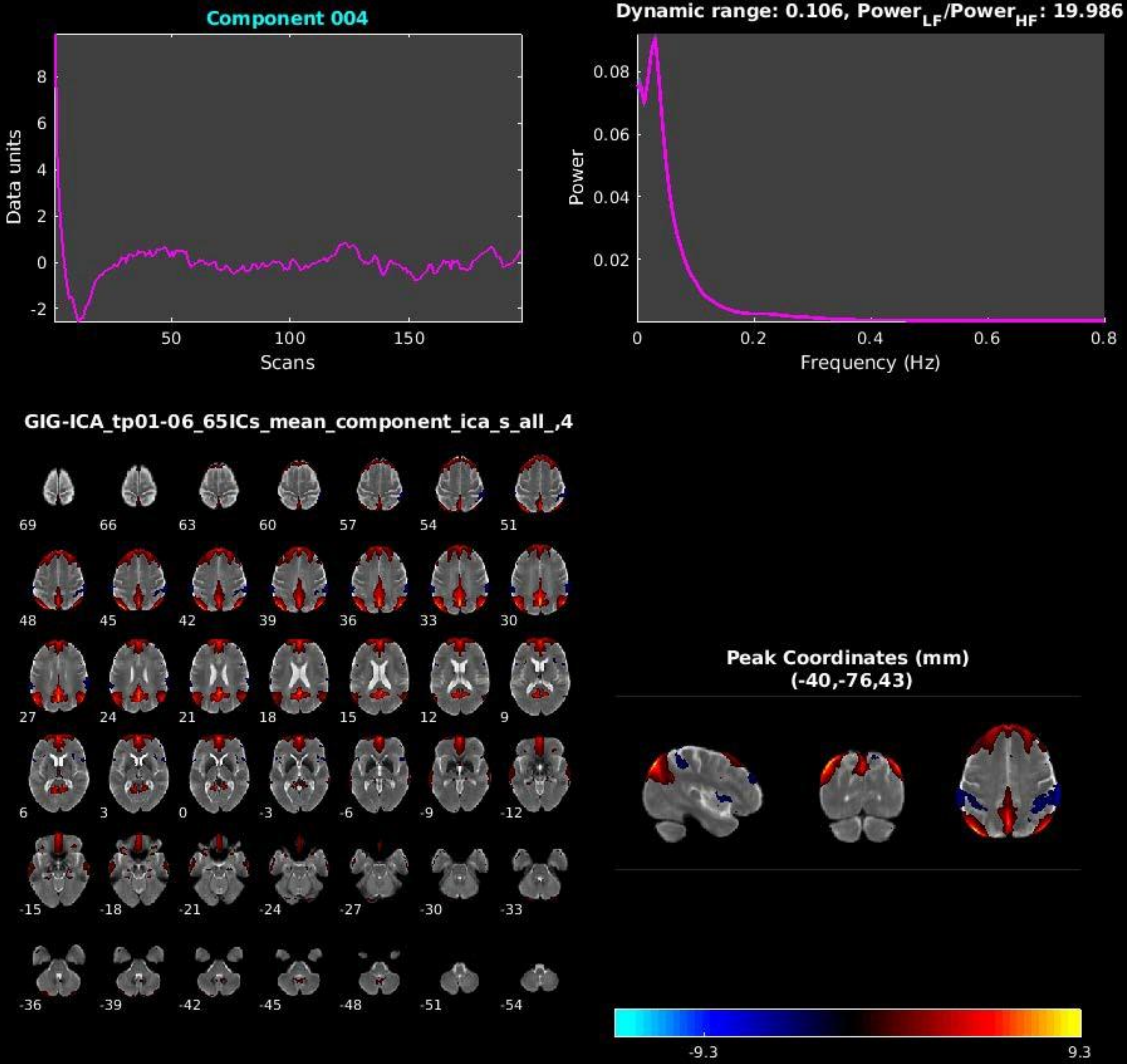
Component 004
Data units
Scans
Dynamic range: 0.106, Power_LF/Power_HF: 19.986
Power
Frequency (Hz)
GIG-ICA_tp01-06_65ICs_mean_component_ica_s_all_,4
Peak Coordinates (mm)
(-40,-76,43)
-9.3
9.3

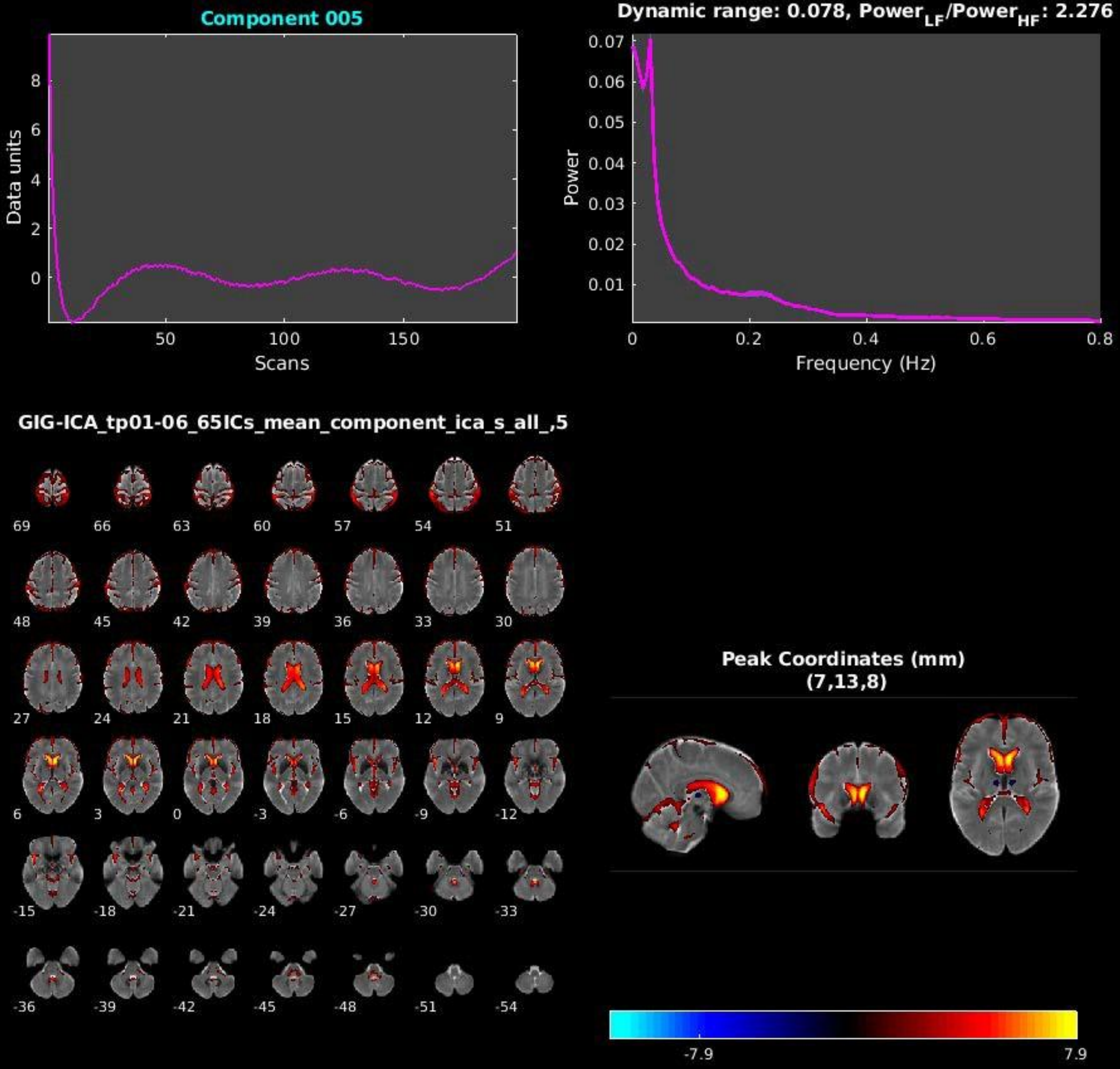

Component 005
Data units
Scans
Dynamic range: 0.078, $Power_{LF}/Power_{HF}$: 2.276
Power
Frequency (Hz)
GIG-ICA_tp01-06_65ICs_mean_component_ica_s_all_,5
Peak Coordinates (mm)
(7,13,8)
-7.9
7.9

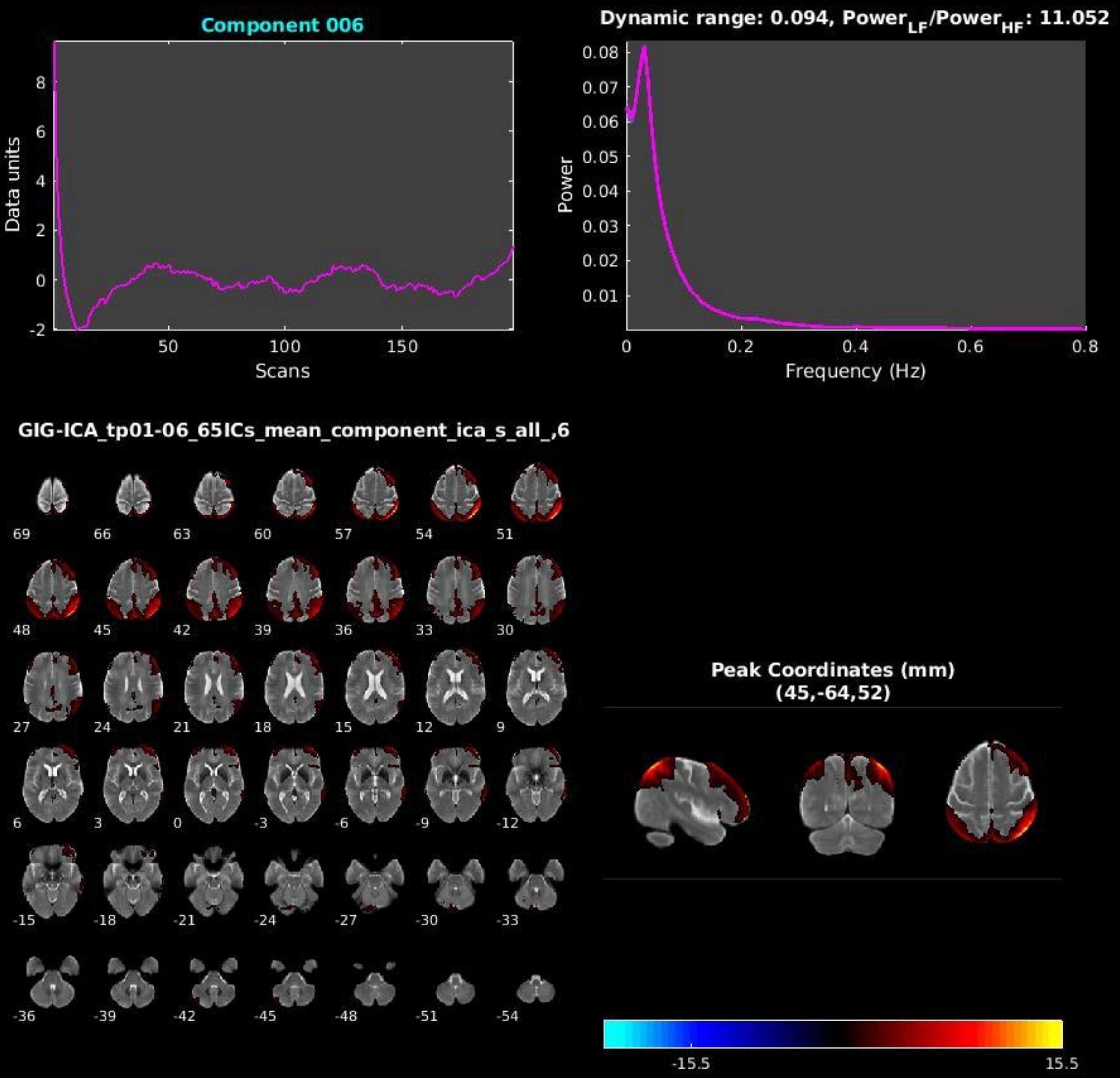

Component 006
Data units
Scans
8
6
4
2
0
-2
50
100
150
Dynamic range: 0.094, Power$_{LF}$/Power$_{HF}$: 11.052
Power
Frequency (Hz)
0.08
0.07
0.06
0.05
0.04
0.03
0.02
0.01
0
0.2
0.4
0.6
0.8
GIG-ICA_tp01-06_65ICs_mean_component_ica_s_all_,6
69 66 63 60 57 54 51
48 45 42 39 36 33 30
27 24 21 18 15 12 9
6 3 0 -3 -6 -9 -12
-15 -18 -21 -24 -27 -30 -33
-36 -39 -42 -45 -48 -51 -54
Peak Coordinates (mm)
(45,-64,52)
-15.5
15.5

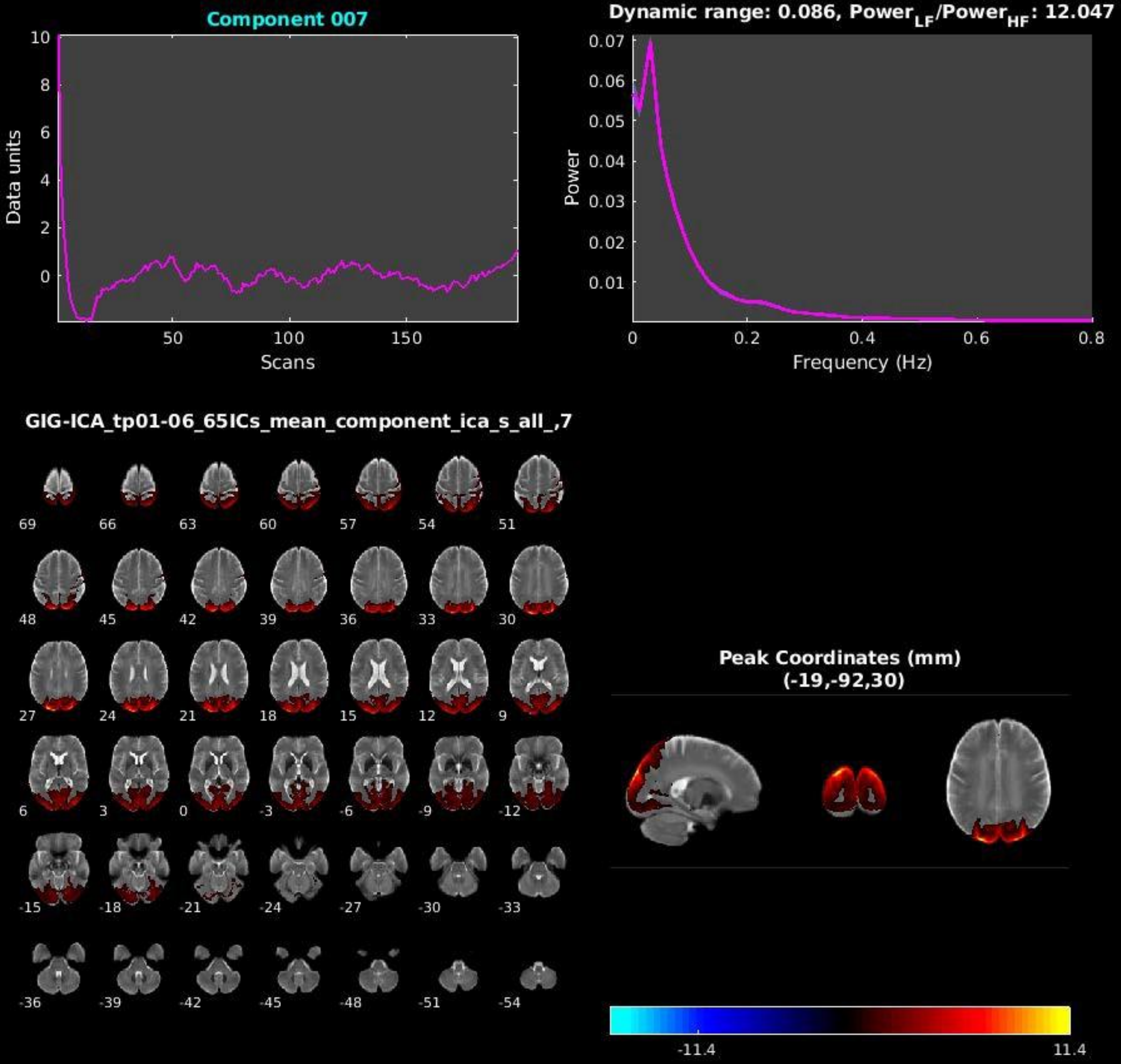

Component 007
Data units
Scans
10
8
6
4
2
0
50
100
150
Dynamic range: 0.086, Power$_{LF}$/Power$_{HF}$: 12.047
Power
Frequency (Hz)
0.07
0.06
0.05
0.04
0.03
0.02
0.01
0
0.2
0.4
0.6
0.8
GIG-ICA_tp01-06_65ICs_mean_component_ica_s_all_,7
69
66
63
60
57
54
51
48
45
42
39
36
33
30
27
24
21
18
15
12
9
6
3
0
-3
-6
-9
-12
-15
-18
-21
-24
-27
-30
-33
-36
-39
-42
-45
-48
-51
-54
Peak Coordinates (mm)
(-19,-92,30)
-11.4
11.4

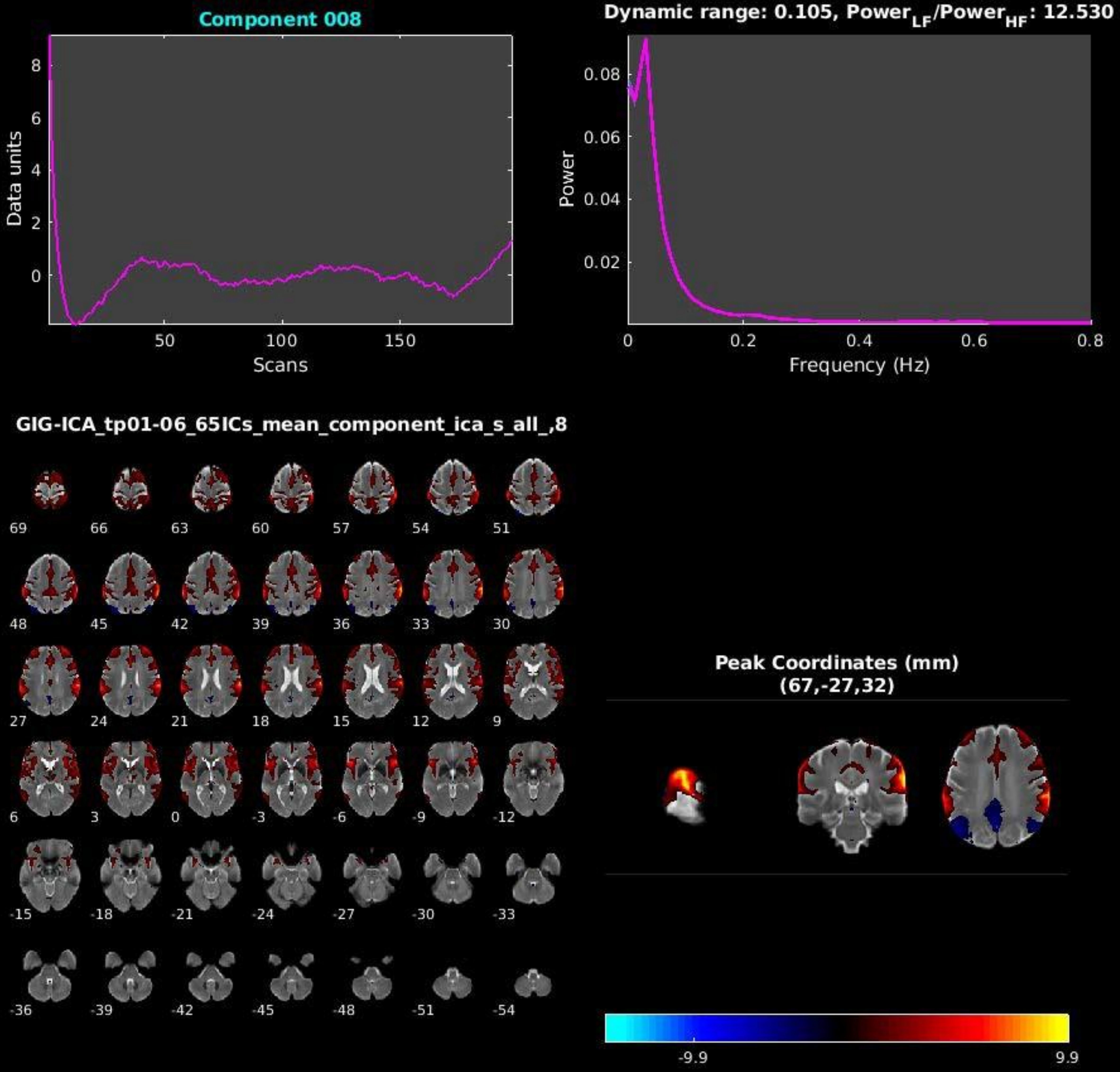
Component 008
Data units
Scans
Dynamic range: 0.105, Power_LF/Power_HF: 12.530
Power
Frequency (Hz)
GIG-ICA_tp01-06_65ICs_mean_component_ica_s_all_,8
Peak Coordinates (mm)
(67,-27,32)
-9.9
9.9

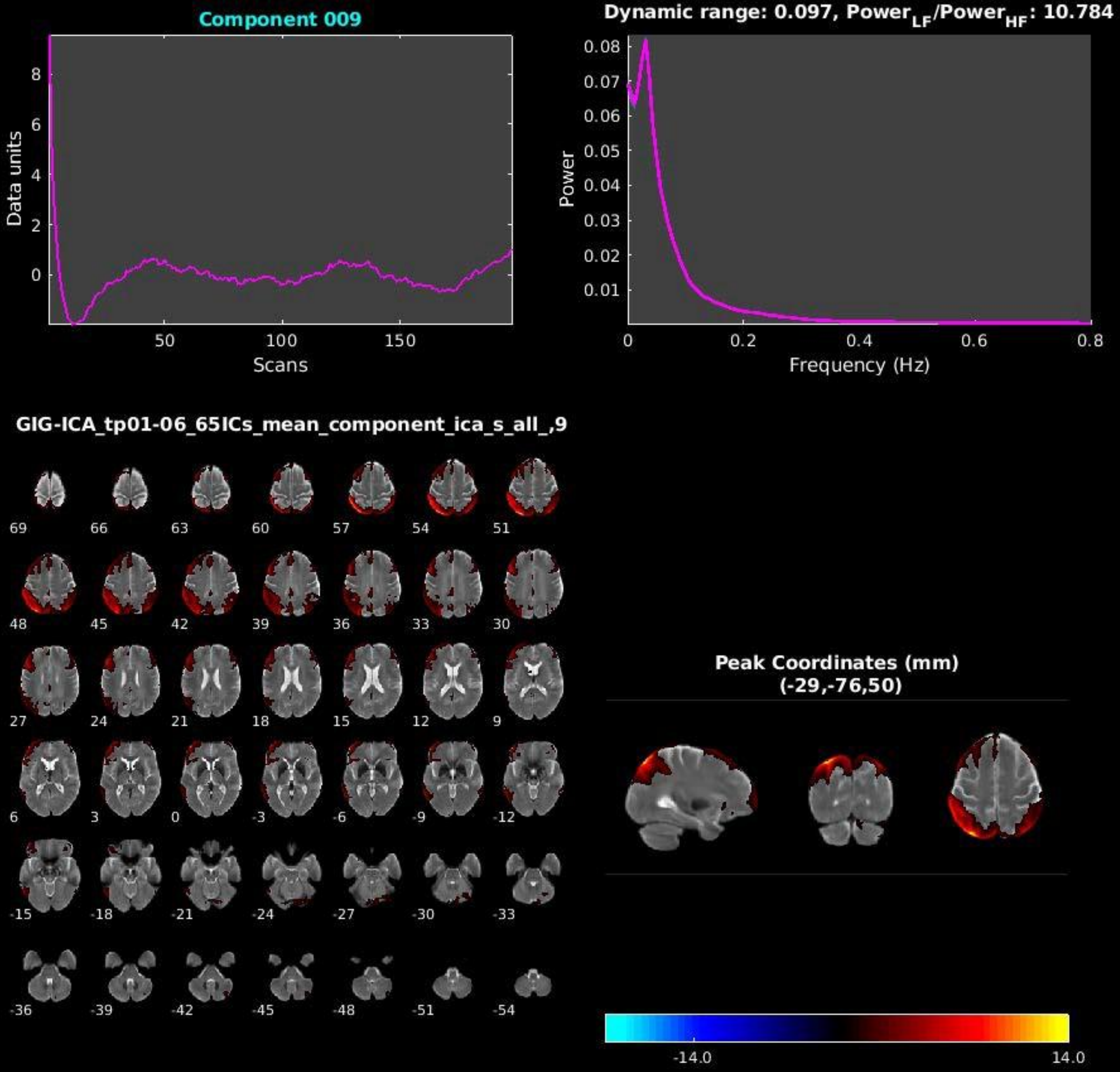

Component 009
Data units
Scans
Dynamic range: 0.097, Power_LF/Power_HF: 10.784
Power
Frequency (Hz)
GIG-ICA_tp01-06_65ICs_mean_component_ica_s_all_,9
Peak Coordinates (mm)
(-29,-76,50)
-14.0
14.0

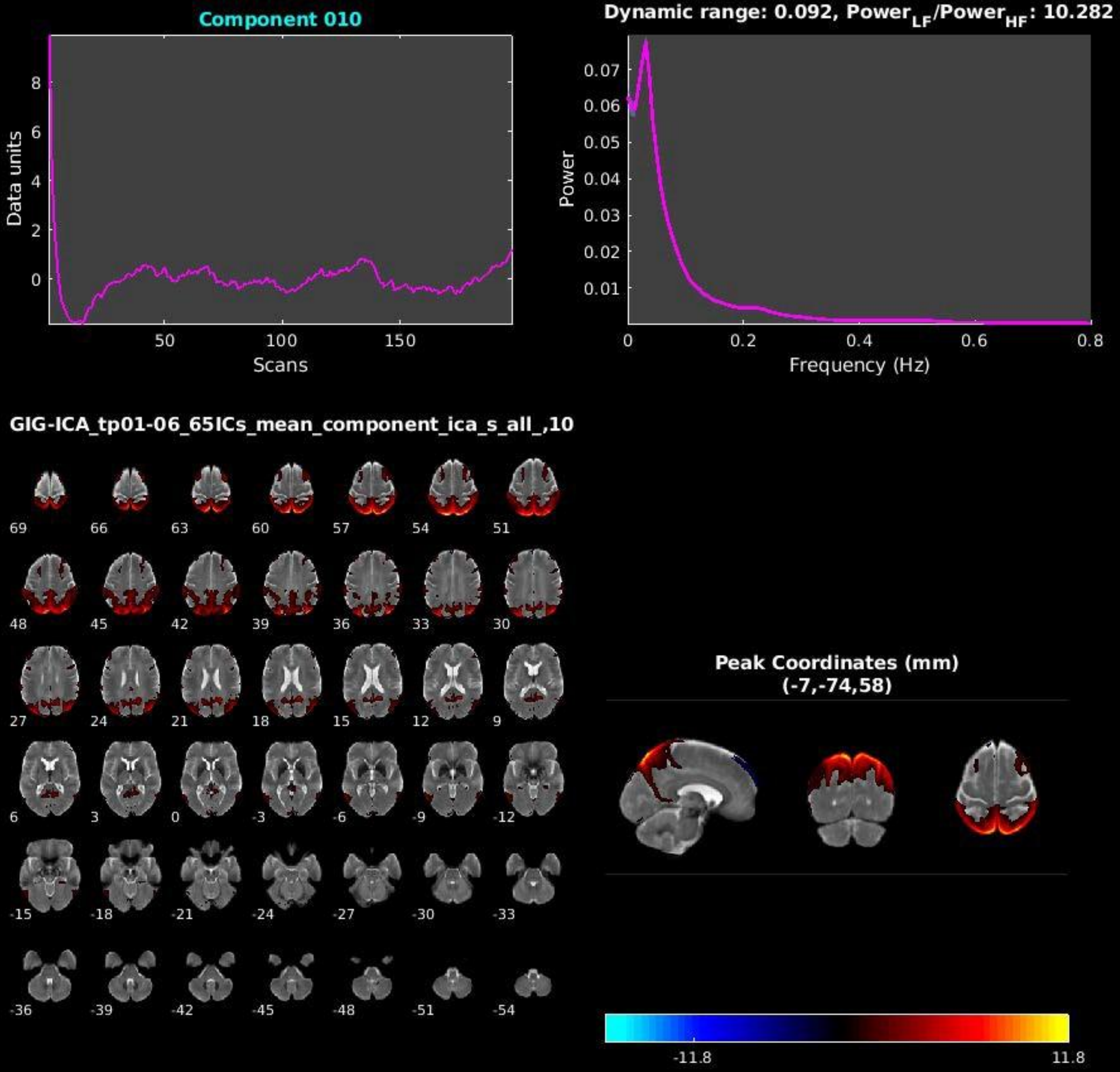

Component 010
Data units
Scans
Dynamic range: 0.092, Power$_{LF}$/Power$_{HF}$: 10.282
Power
Frequency (Hz)
GIG-ICA_tp01-06_65ICs_mean_component_ica_s_all_,10
Peak Coordinates (mm)
(-7,-74,58)
-11.8
11.8

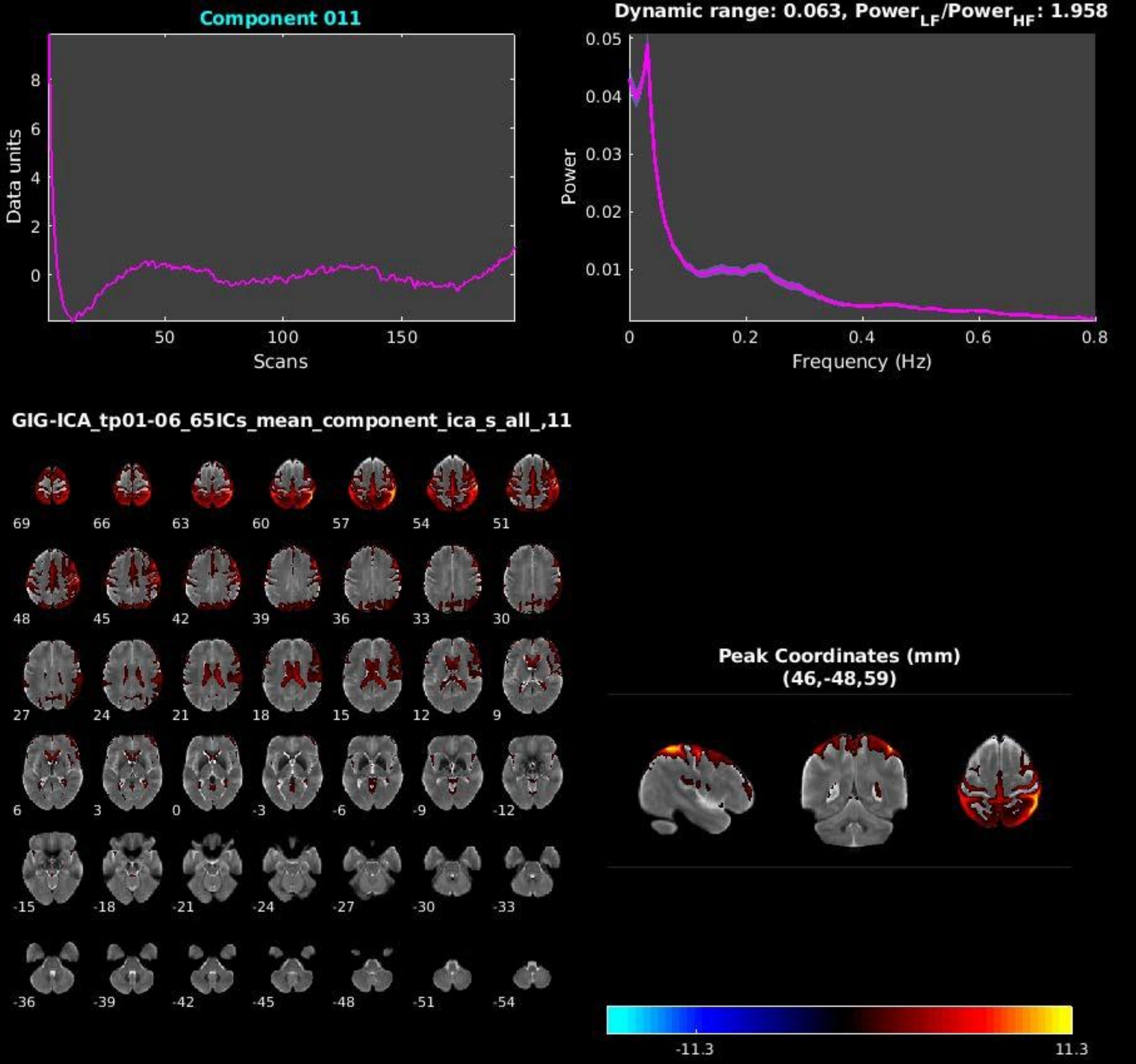

Component 011
Data units
Scans
Dynamic range: 0.063, Power_LF/Power_HF: 1.958
Power
Frequency (Hz)
GIG-ICA_tp01-06_65ICs_mean_component_ica_s_all_,11
Peak Coordinates (mm)
(46,-48,59)
-11.3
11.3

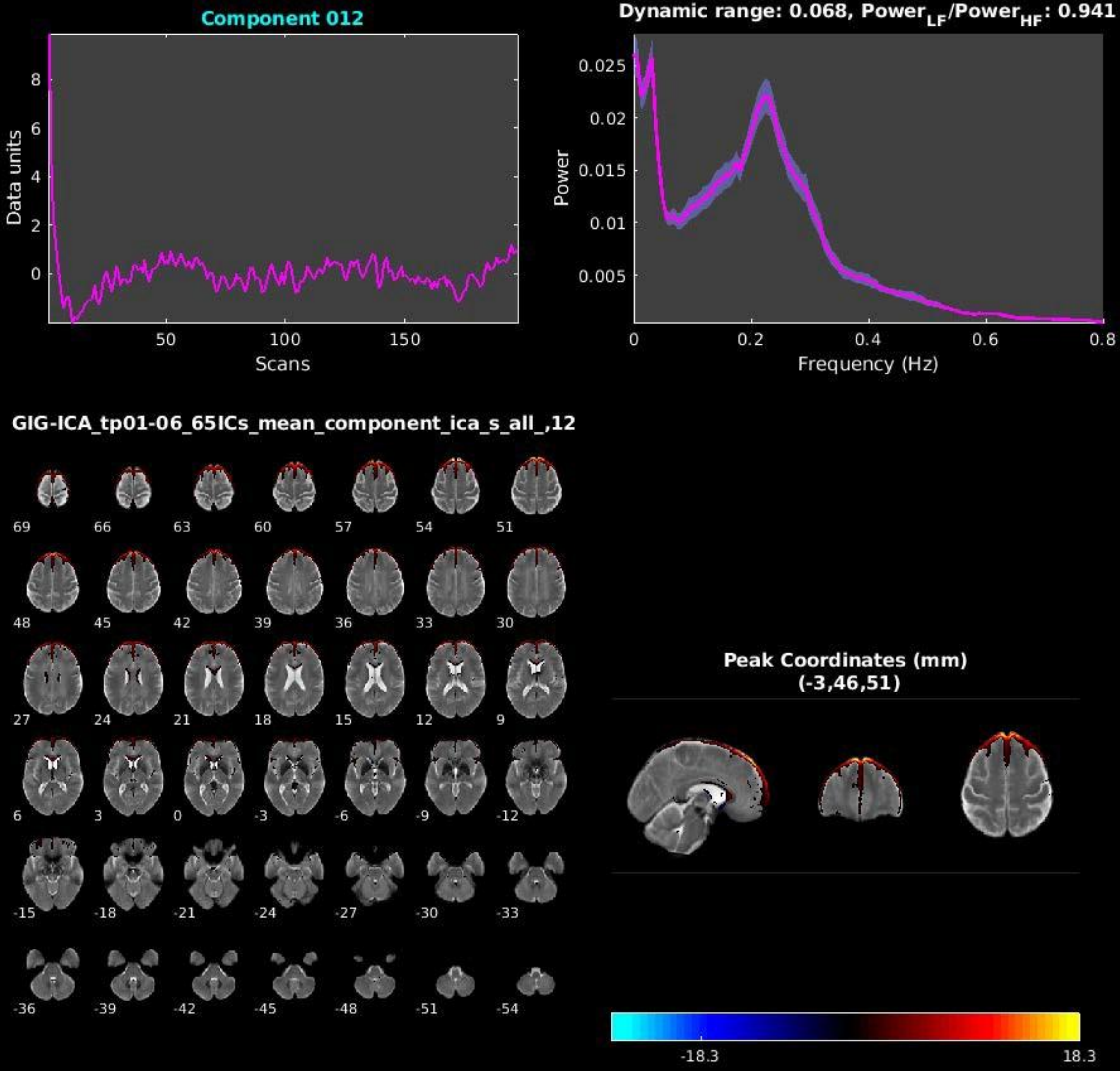

Component 012
Data units
Scans
Dynamic range: 0.068, Power_LF/Power_HF: 0.941
Power
Frequency (Hz)
GIG-ICA_tp01-06_65ICs_mean_component_ica_s_all_,12
Peak Coordinates (mm)
(-3,46,51)
-18.3
18.3

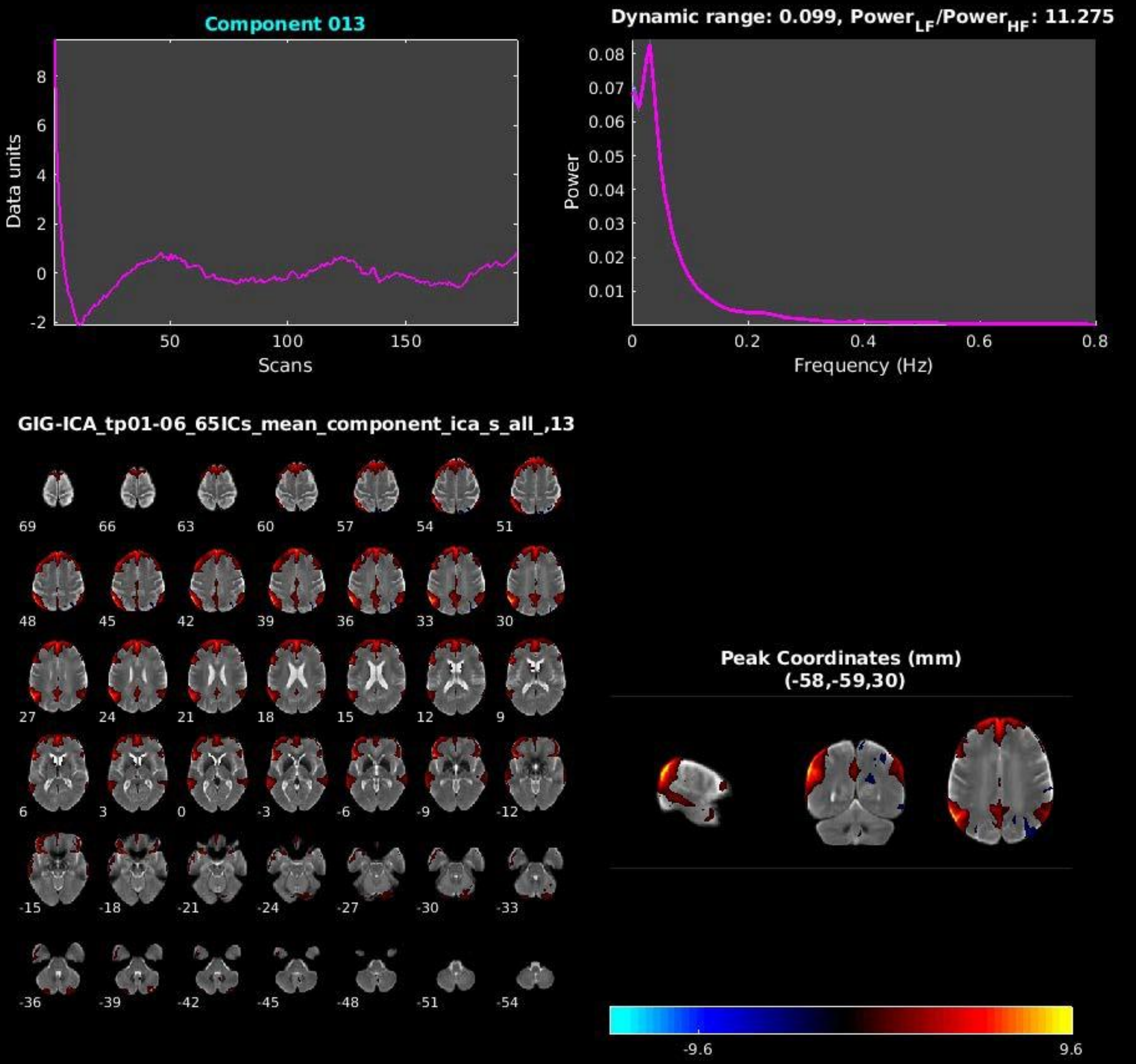
Component 013
Data units
Scans
8
6
4
2
0
-2
50
100
150
Dynamic range: 0.099, Power$_{LF}$/Power$_{HF}$: 11.275
Power
Frequency (Hz)
0.08
0.07
0.06
0.05
0.04
0.03
0.02
0.01
0
0.2
0.4
0.6
0.8
GIG-ICA_tp01-06_65ICs_mean_component_ica_s_all_,13
69 66 63 60 57 54 51
48 45 42 39 36 33 30
27 24 21 18 15 12 9
6 3 0 -3 -6 -9 -12
-15 -18 -21 -24 -27 -30 -33
-36 -39 -42 -45 -48 -51 -54
Peak Coordinates (mm)
(-58,-59,30)
-9.6
9.6

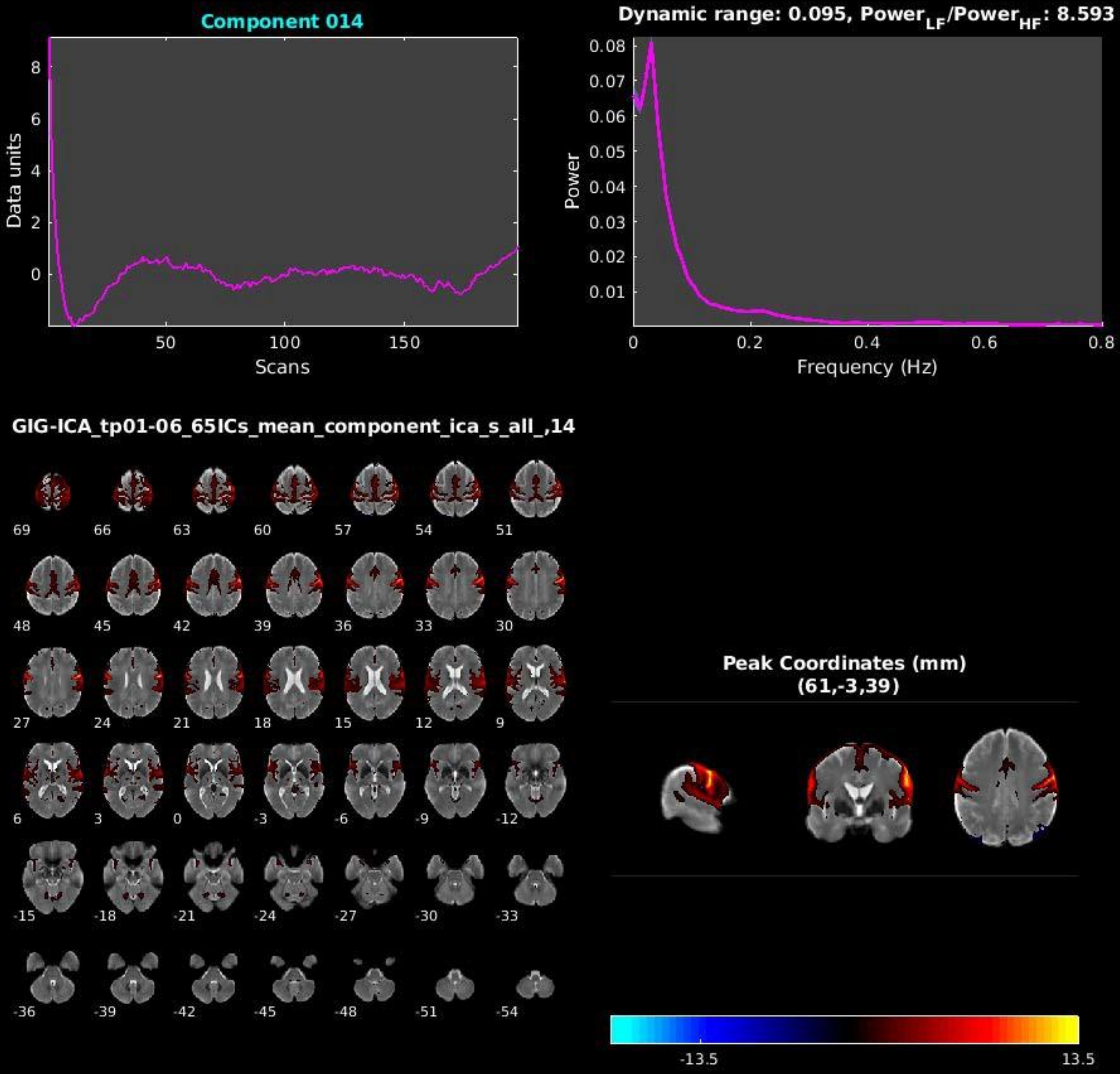
Component 014
Data units
Scans
Dynamic range: 0.095, $Power_{LF}/Power_{HF}$: 8.593
Power
Frequency (Hz)
GIG-ICA_tp01-06_65ICs_mean_component_ica_s_all_,14
Peak Coordinates (mm)
(61,-3,39)
-13.5
13.5

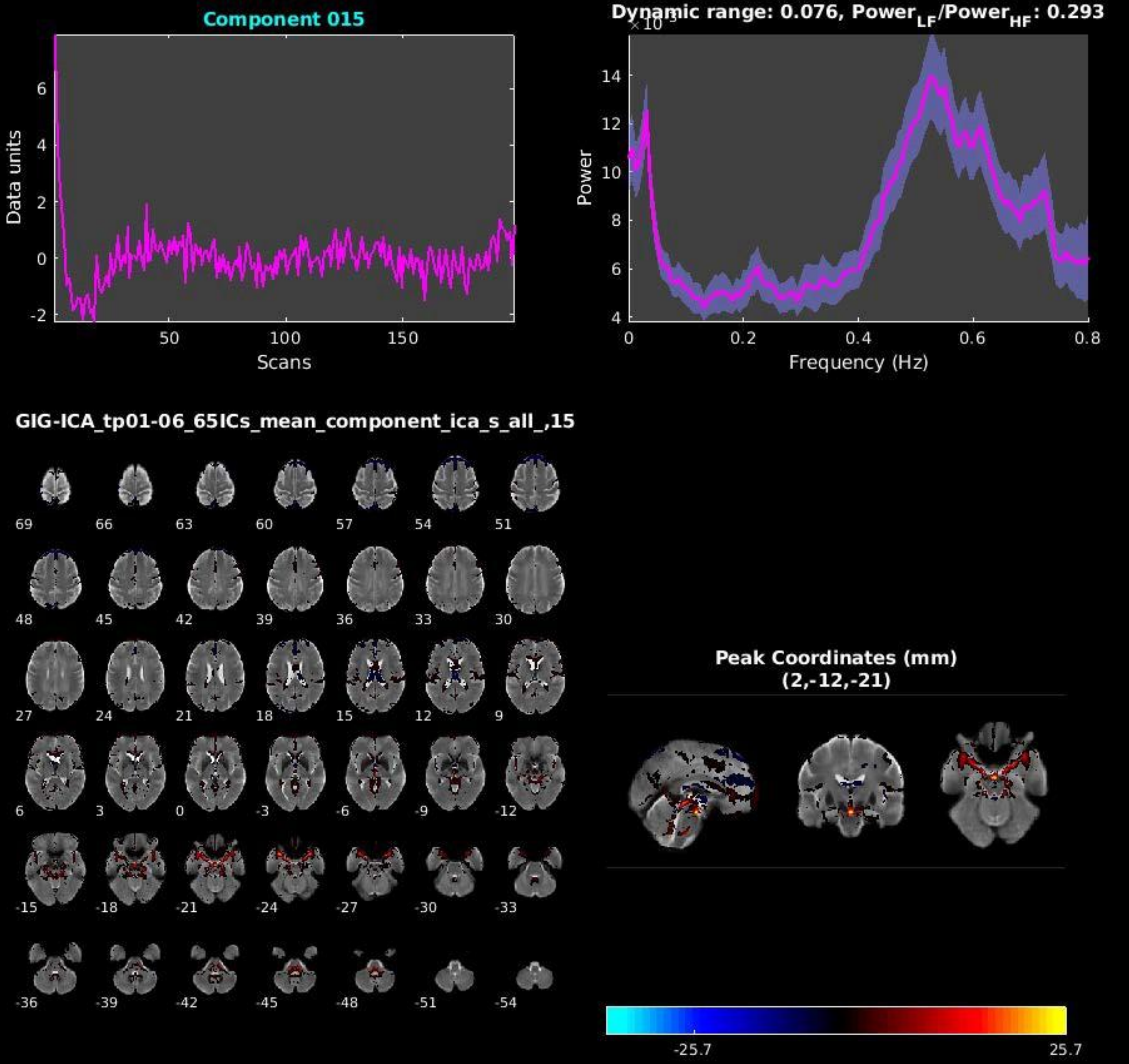

Component 015
Data units
Scans
Dynamic range: 0.076, Power_LF/Power_HF: 0.293
×10^-3
Power
Frequency (Hz)
GIG-ICA_tp01-06_65ICs_mean_component_ica_s_all_,15
Peak Coordinates (mm)
(2,-12,-21)
-25.7
25.7

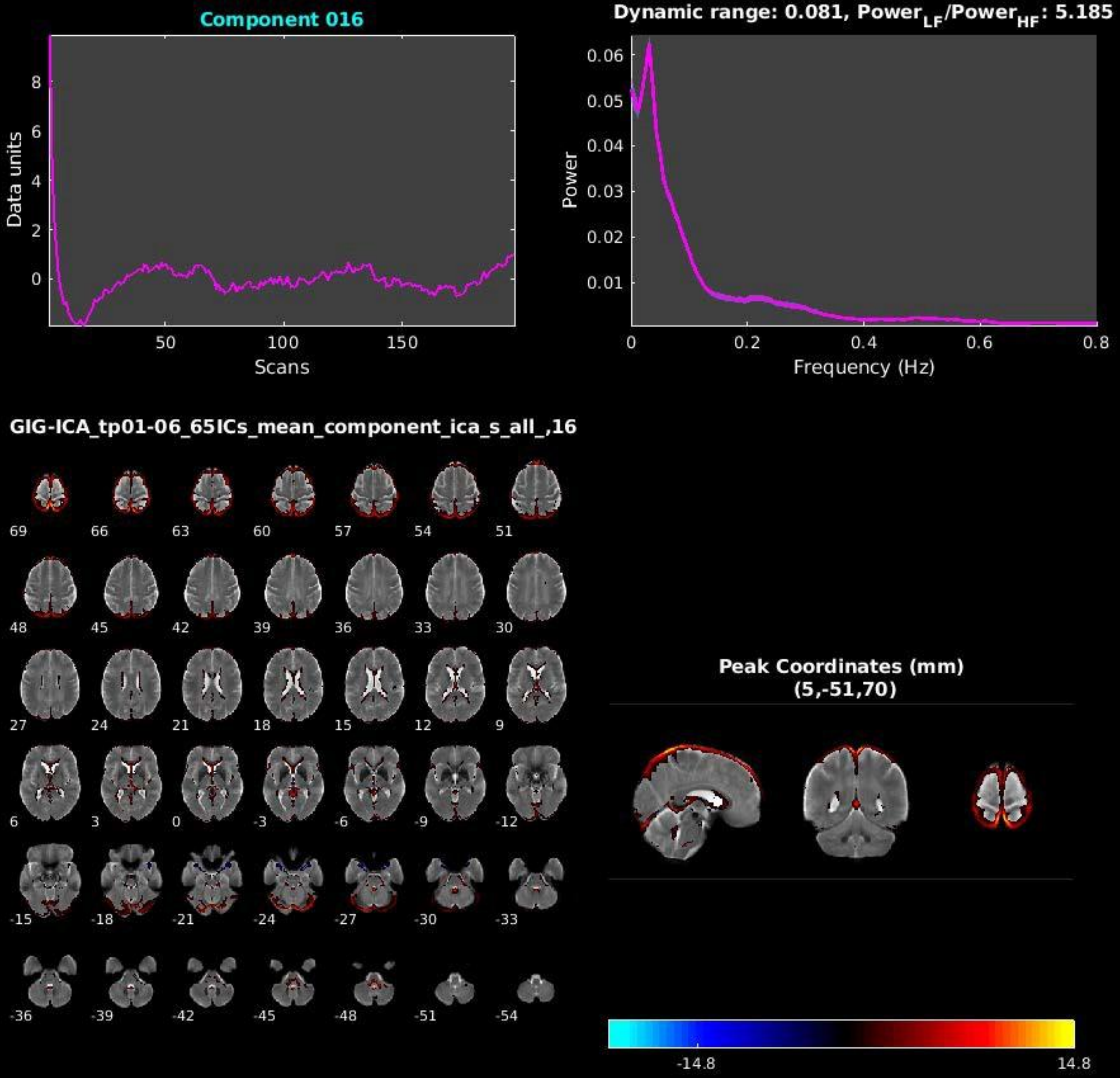
Component 016
Data units
Scans
0
2
4
6
8
50
100
150
Dynamic range: 0.081, Power$_{LF}$/Power$_{HF}$: 5.185
Power
Frequency (Hz)
0.01
0.02
0.03
0.04
0.05
0.06
0
0.2
0.4
0.6
0.8
GIG-ICA_tp01-06_65ICs_mean_component_ica_s_all_,16
69
66
63
60
57
54
51
48
45
42
39
36
33
30
27
24
21
18
15
12
9
6
3
0
-3
-6
-9
-12
-15
-18
-21
-24
-27
-30
-33
-36
-39
-42
-45
-48
-51
-54
Peak Coordinates (mm)
(5,-51,70)
-14.8
14.8

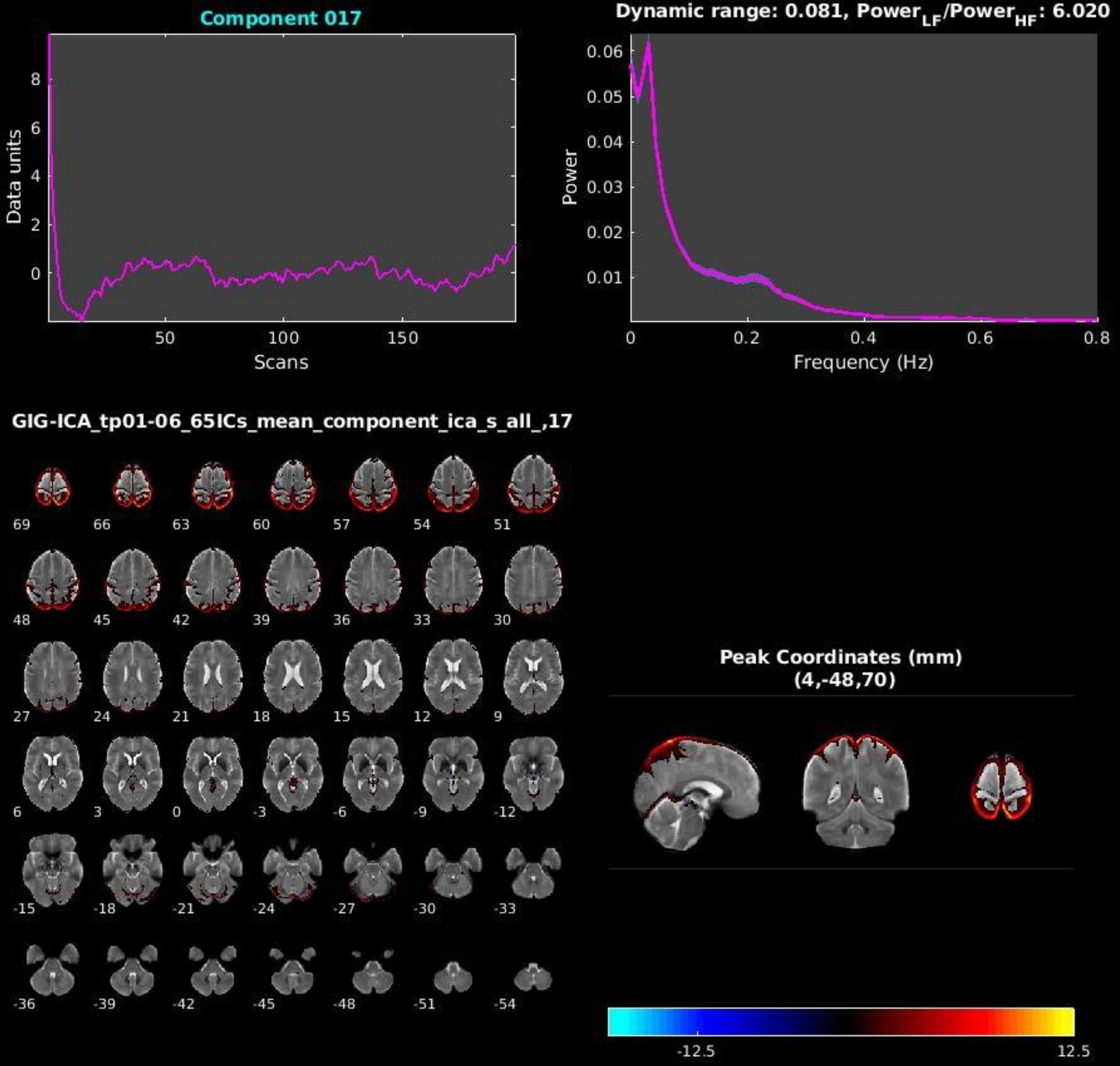

Component 017
Data units
Scans
50
100
150
Dynamic range: 0.081, Power_LF/Power_HF: 6.020
Power
Frequency (Hz)
0.06
0.05
0.04
0.03
0.02
0.01
0
0.2
0.4
0.6
0.8
GIG-ICA_tp01-06_65ICs_mean_component_ica_s_all_,17
69 66 63 60 57 54 51
48 45 42 39 36 33 30
27 24 21 18 15 12 9
6 3 0 -3 -6 -9 -12
-15 -18 -21 -24 -27 -30 -33
-36 -39 -42 -45 -48 -51 -54
Peak Coordinates (mm)
(4,-48,70)
-12.5
12.5

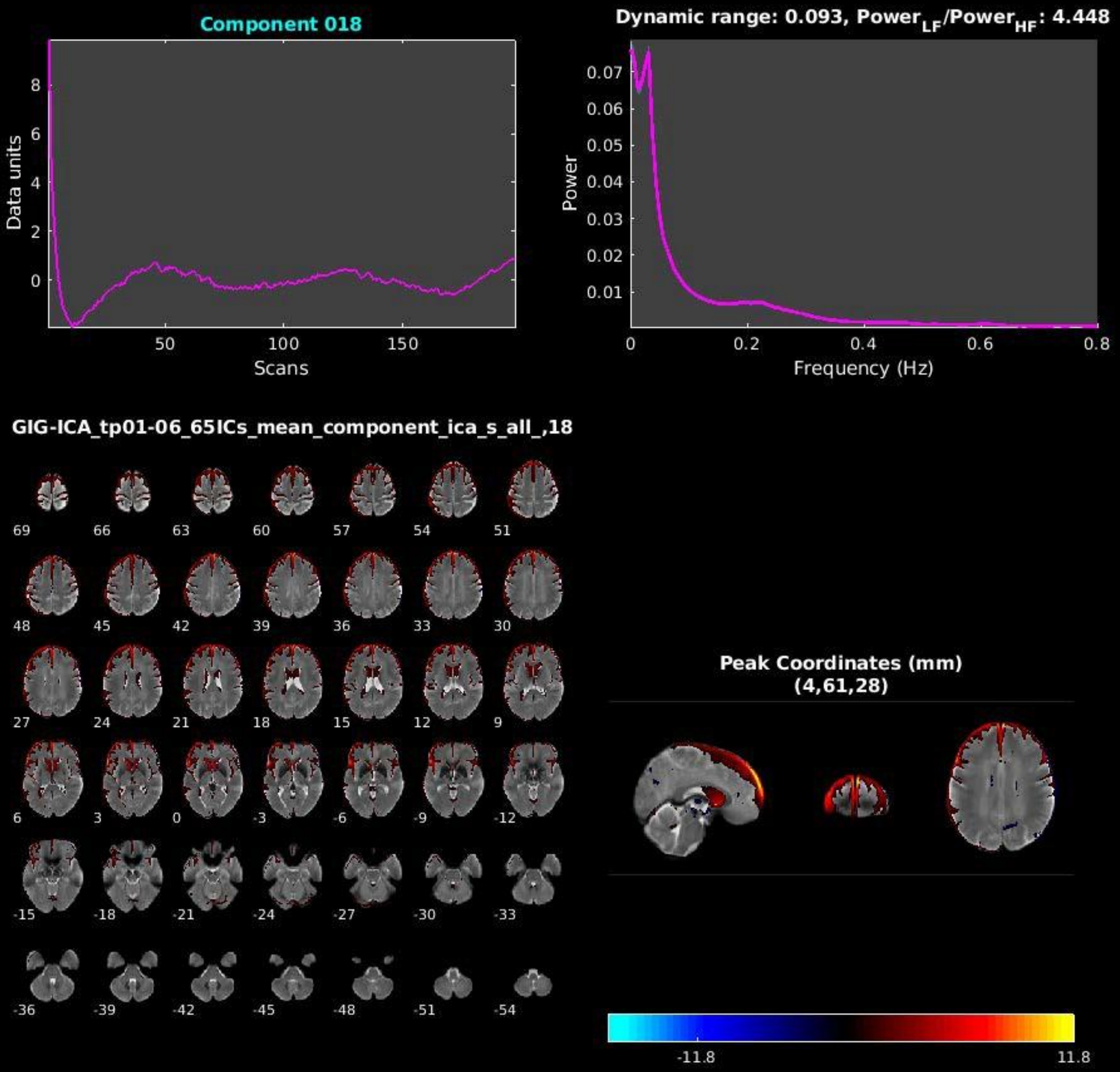

Component 018
Data units
Scans
Dynamic range: 0.093, Power$_{LF}$/Power$_{HF}$: 4.448
Power
Frequency (Hz)
GIG-ICA_tp01-06_65ICs_mean_component_ica_s_all_,18
Peak Coordinates (mm)
(4,61,28)
-11.8
11.8

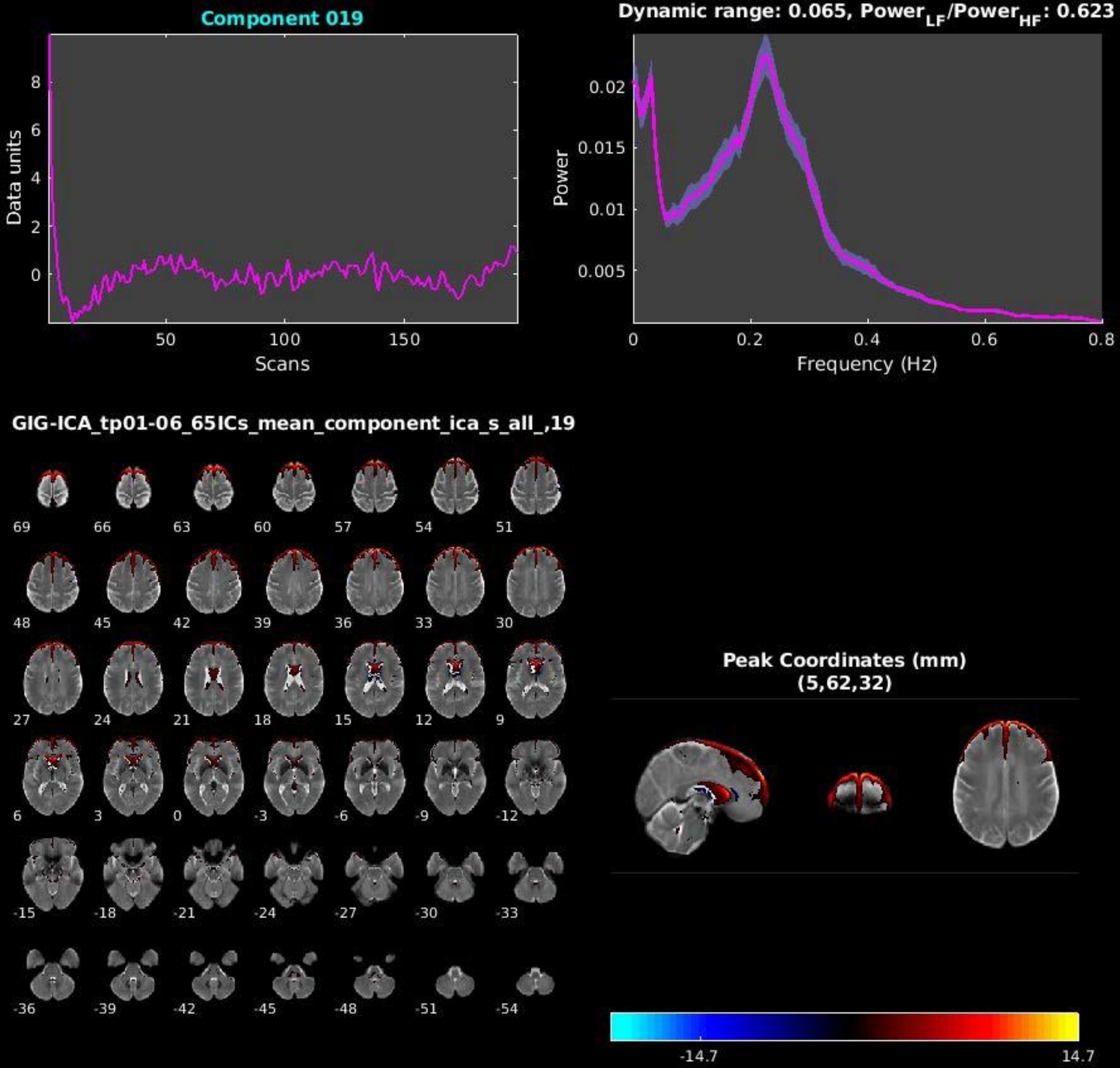

Component 019
Data units
Scans
Dynamic range: 0.065, Power$_{LF}$/Power$_{HF}$: 0.623
Power
Frequency (Hz)
GIG-ICA_tp01-06_65ICs_mean_component_ica_s_all_,19
Peak Coordinates (mm)
(5,62,32)
-14.7
14.7

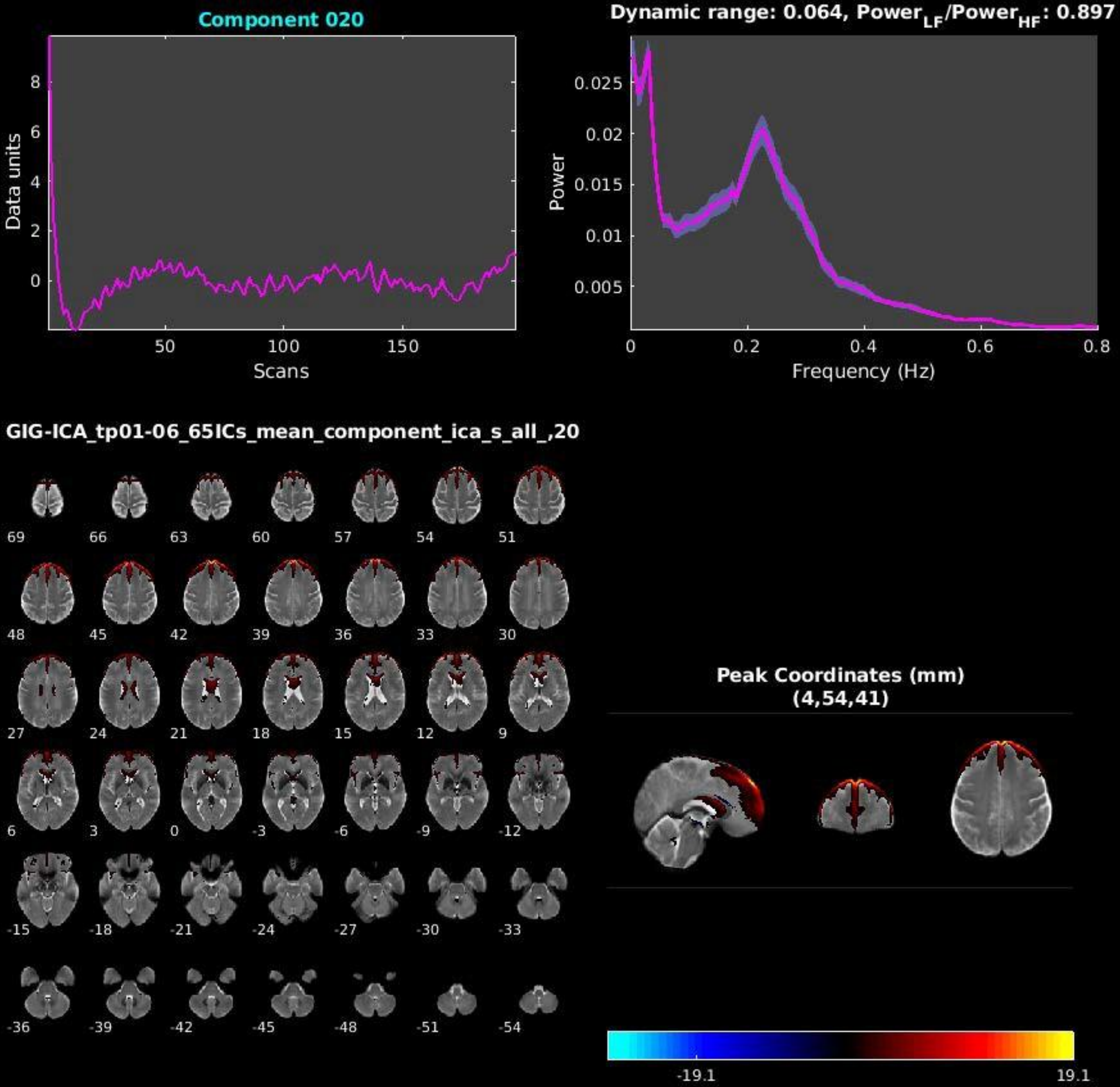

Component 020
Data units
Scans
50
100
150
8
6
4
2
0
Dynamic range: 0.064, Power$_{LF}$/Power$_{HF}$: 0.897
Power
Frequency (Hz)
0.025
0.02
0.015
0.01
0.005
0
0.2
0.4
0.6
0.8
GIG-ICA_tp01-06_65ICs_mean_component_ica_s_all_,20
69
66
63
60
57
54
51
48
45
42
39
36
33
30
27
24
21
18
15
12
9
6
3
0
-3
-6
-9
-12
-15
-18
-21
-24
-27
-30
-33
-36
-39
-42
-45
-48
-51
-54
Peak Coordinates (mm)
(4,54,41)
-19.1
19.1

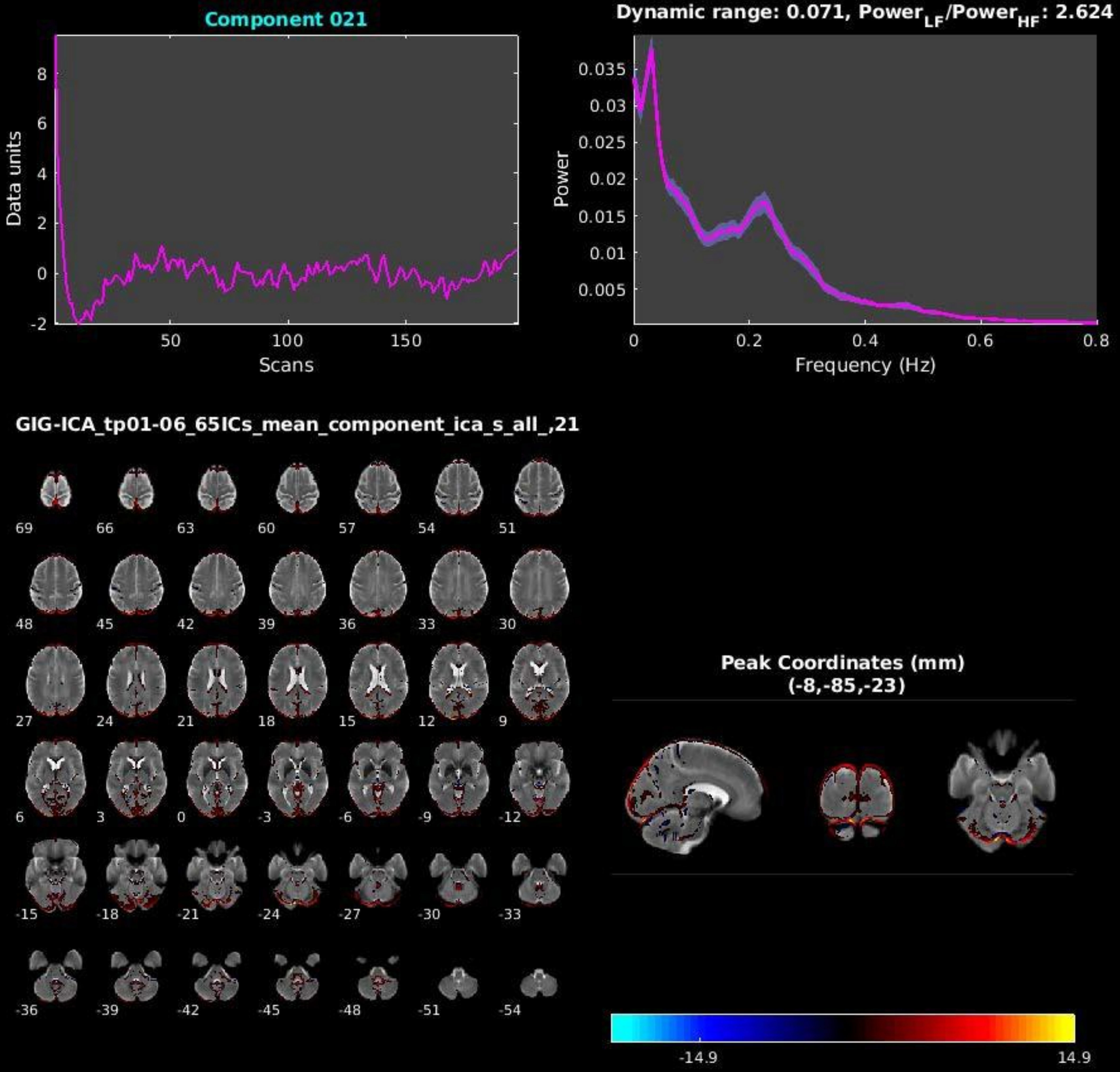
Component 021
Data units
Scans
8
6
4
2
0
-2
50
100
150
Dynamic range: 0.071, Power_LF/Power_HF: 2.624
Power
Frequency (Hz)
0.035
0.03
0.025
0.02
0.015
0.01
0.005
0
0.2
0.4
0.6
0.8
GIG-ICA_tp01-06_65ICs_mean_component_ica_s_all_,21
69 66 63 60 57 54 51
48 45 42 39 36 33 30
27 24 21 18 15 12 9
6 3 0 -3 -6 -9 -12
-15 -18 -21 -24 -27 -30 -33
-36 -39 -42 -45 -48 -51 -54
Peak Coordinates (mm)
(-8,-85,-23)
-14.9
14.9

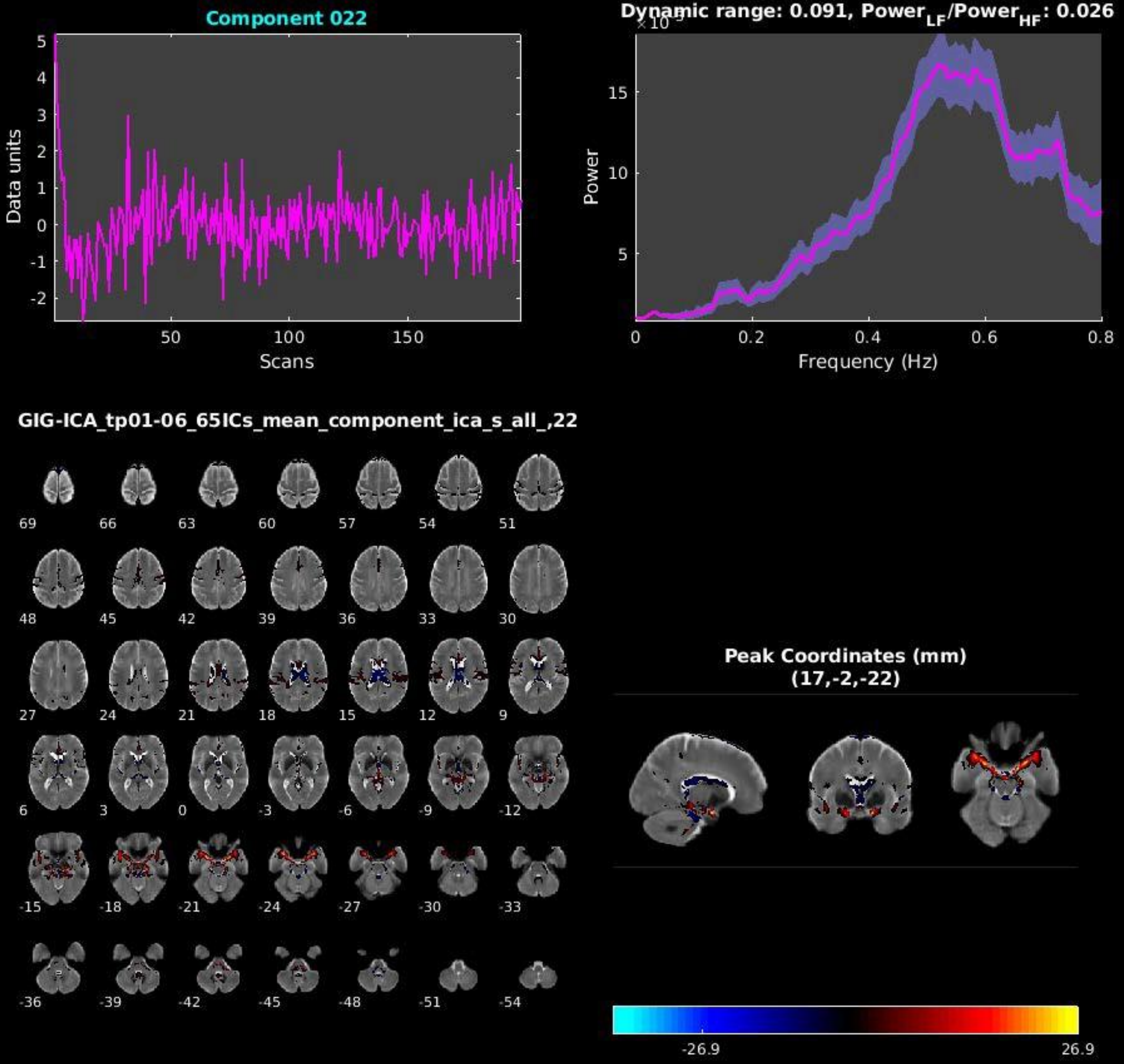

Component 022
Data units
Scans
Dynamic range: 0.091, Power$_{LF}$/Power$_{HF}$: 0.026
Power
Frequency (Hz)
GIG-ICA_tp01-06_65ICs_mean_component_ica_s_all_,22
Peak Coordinates (mm)
(17,-2,-22)
-26.9
26.9

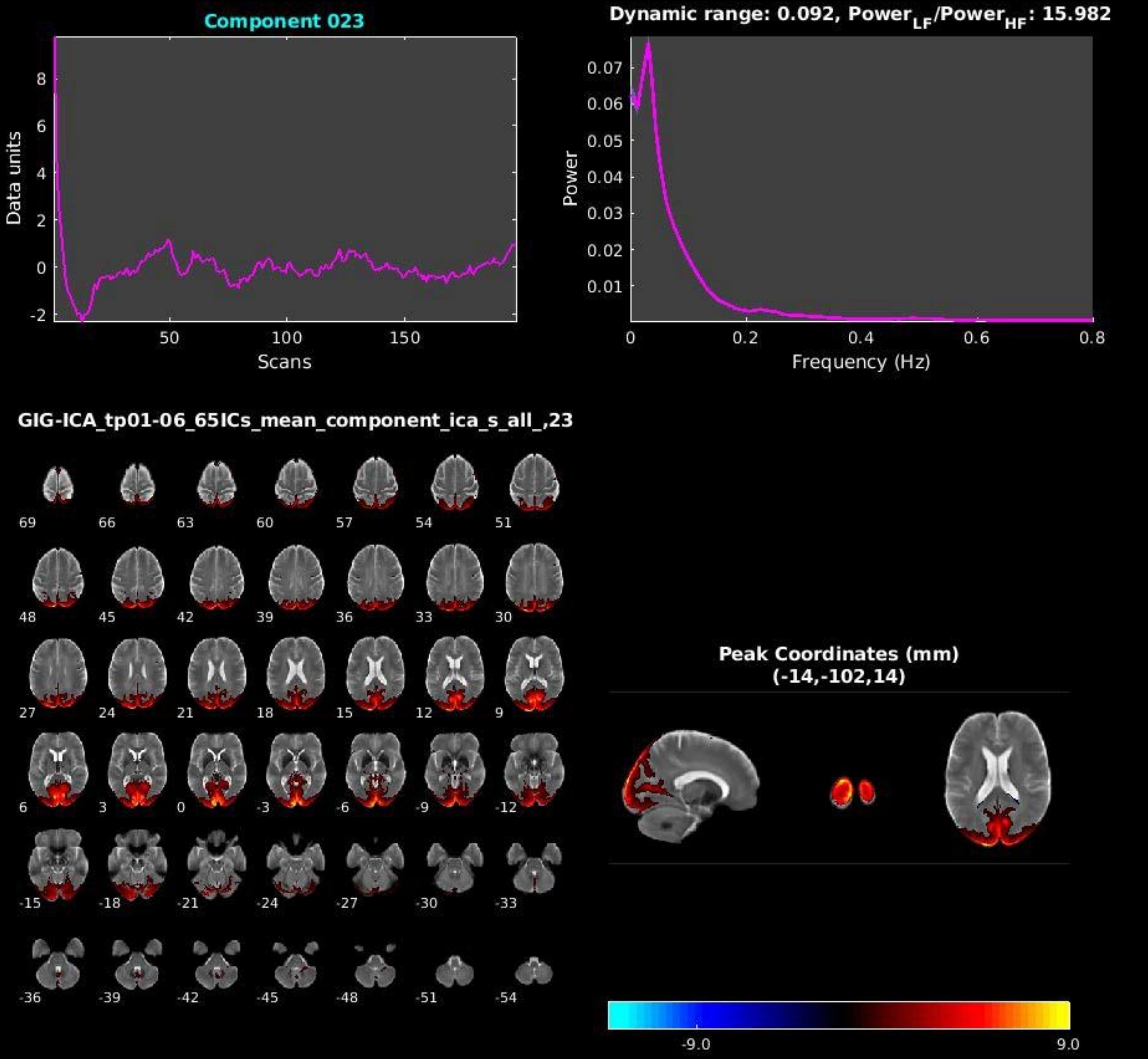
Component 023
Data units
Scans
Dynamic range: 0.092, Power$_{LF}$/Power$_{HF}$: 15.982
Power
Frequency (Hz)
GIG-ICA_tp01-06_65ICs_mean_component_ica_s_all_,23
Peak Coordinates (mm)
(-14,-102,14)
-9.0
9.0

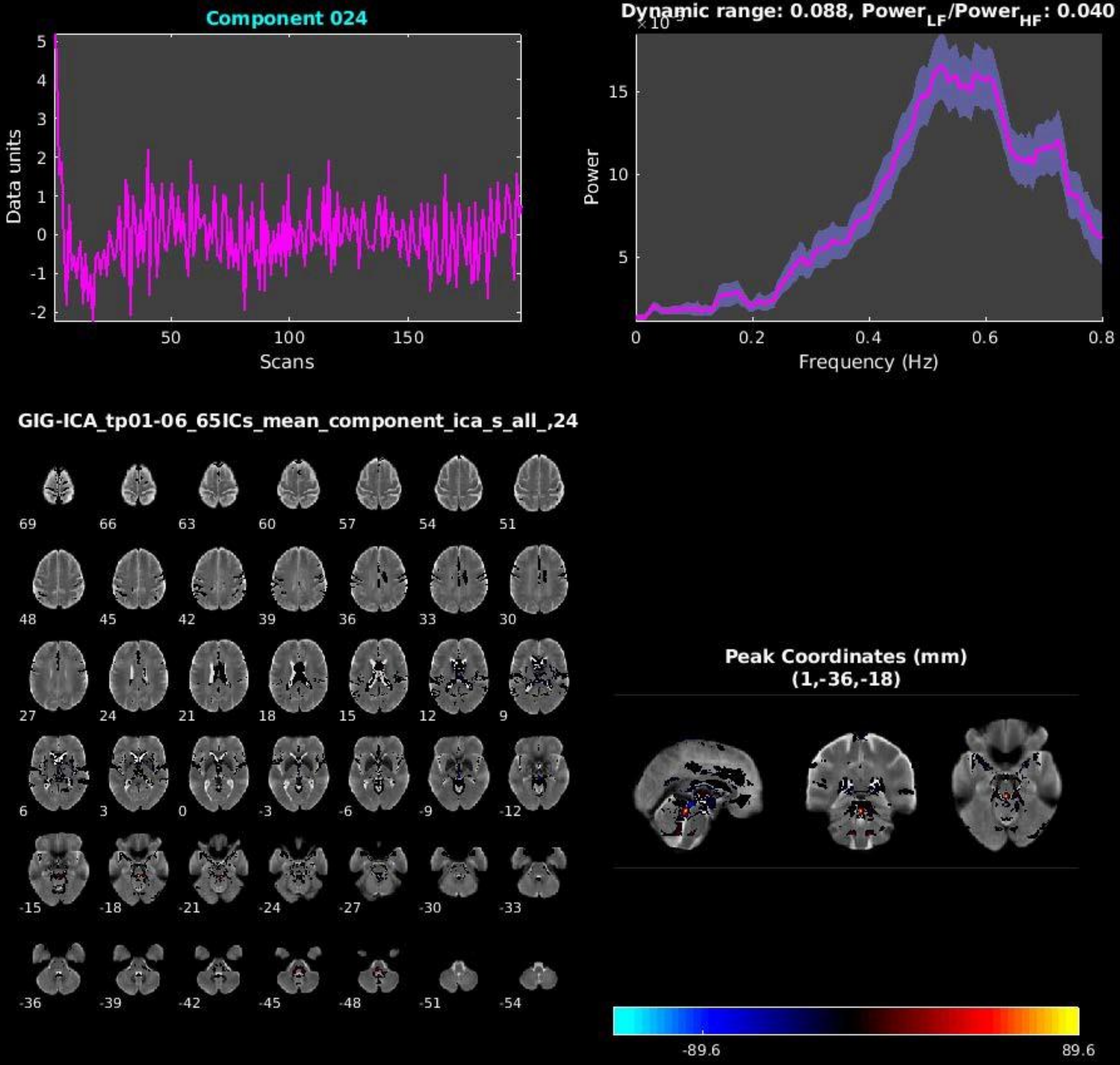
Component 024
Data units
Scans
5
4
3
2
1
0
-1
-2
50
100
150
Dynamic range: 0.088, Power_LF/Power_HF: 0.040
Power
Frequency (Hz)
15
10
5
0
0.2
0.4
0.6
0.8
GIG-ICA_tp01-06_65ICs_mean_component_ica_s_all_,24
69
66
63
60
57
54
51
48
45
42
39
36
33
30
27
24
21
18
15
12
9
6
3
0
-3
-6
-9
-12
-15
-18
-21
-24
-27
-30
-33
-36
-39
-42
-45
-48
-51
-54
Peak Coordinates (mm)
(1,-36,-18)
-89.6
89.6

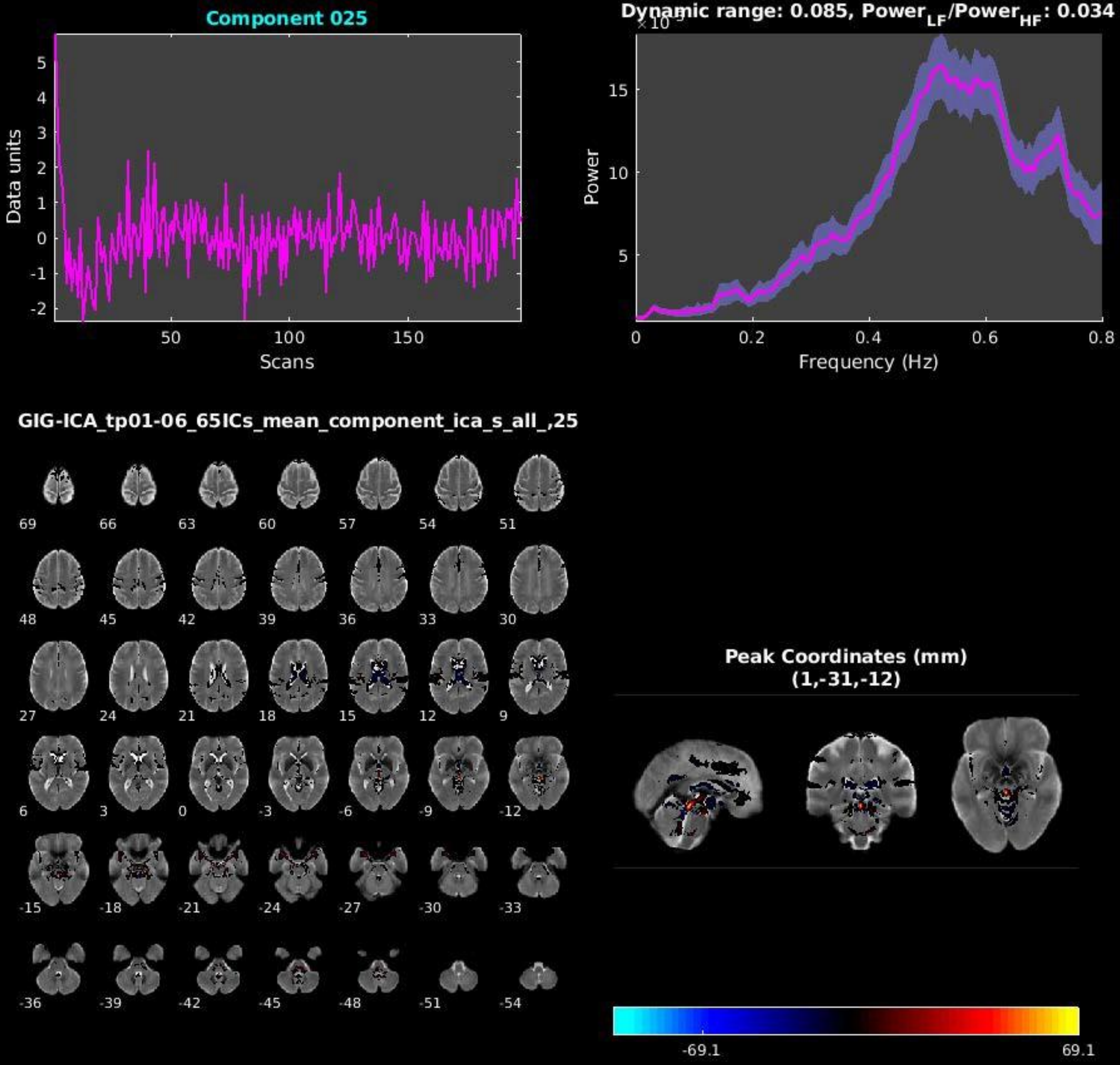
Component 025
Data units
Scans
Dynamic range: 0.085, Power$_{LF}$/Power$_{HF}$: 0.034
Power
Frequency (Hz)
GIG-ICA_tp01-06_65ICs_mean_component_ica_s_all_,25
Peak Coordinates (mm)
(1,-31,-12)
-69.1
69.1

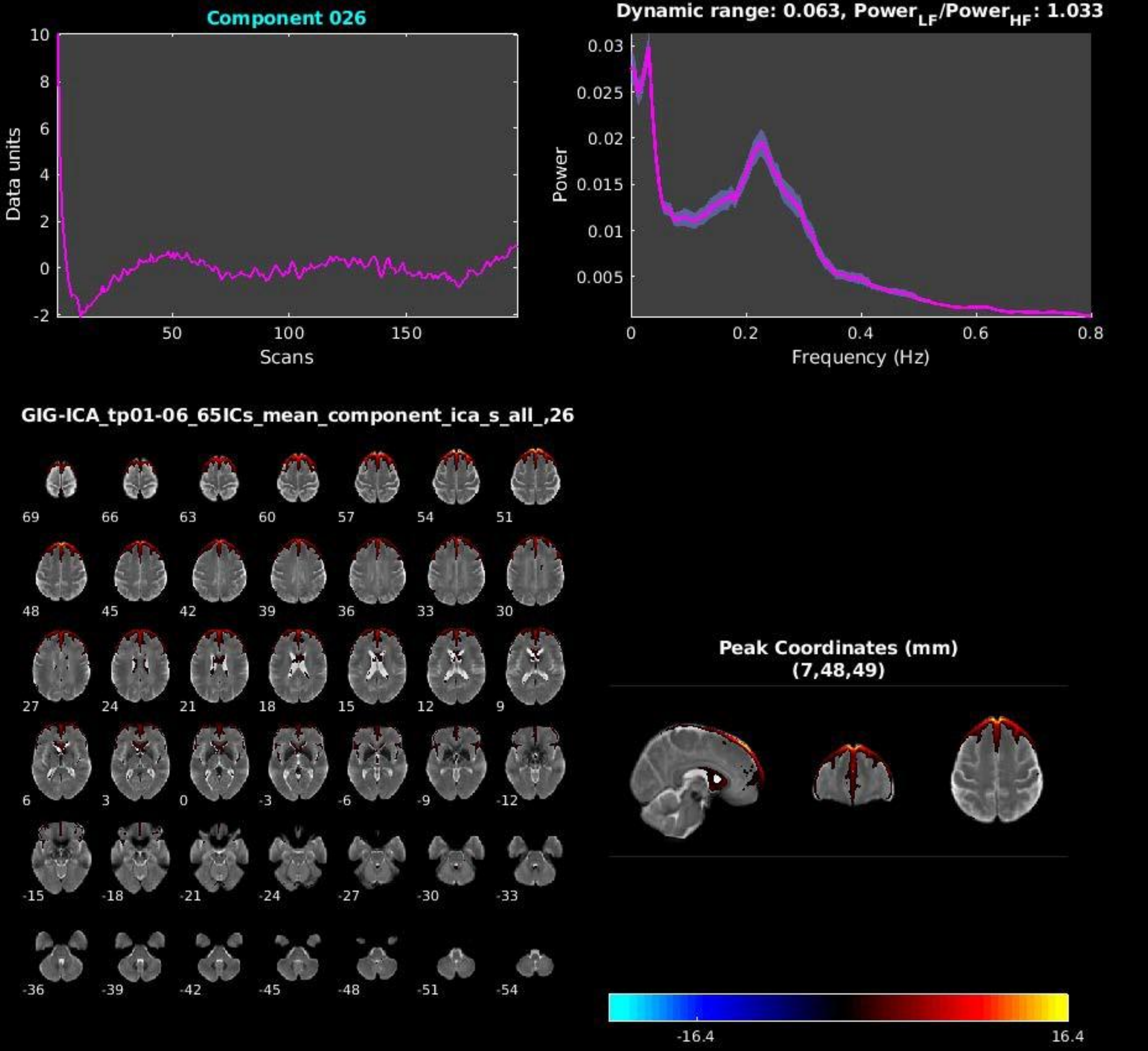
Component 026
Data units
Scans
Dynamic range: 0.063, Power$_{LF}$/Power$_{HF}$: 1.033
Power
Frequency (Hz)
GIG-ICA_tp01-06_65ICs_mean_component_ica_s_all_,26
Peak Coordinates (mm)
(7,48,49)
-16.4
16.4

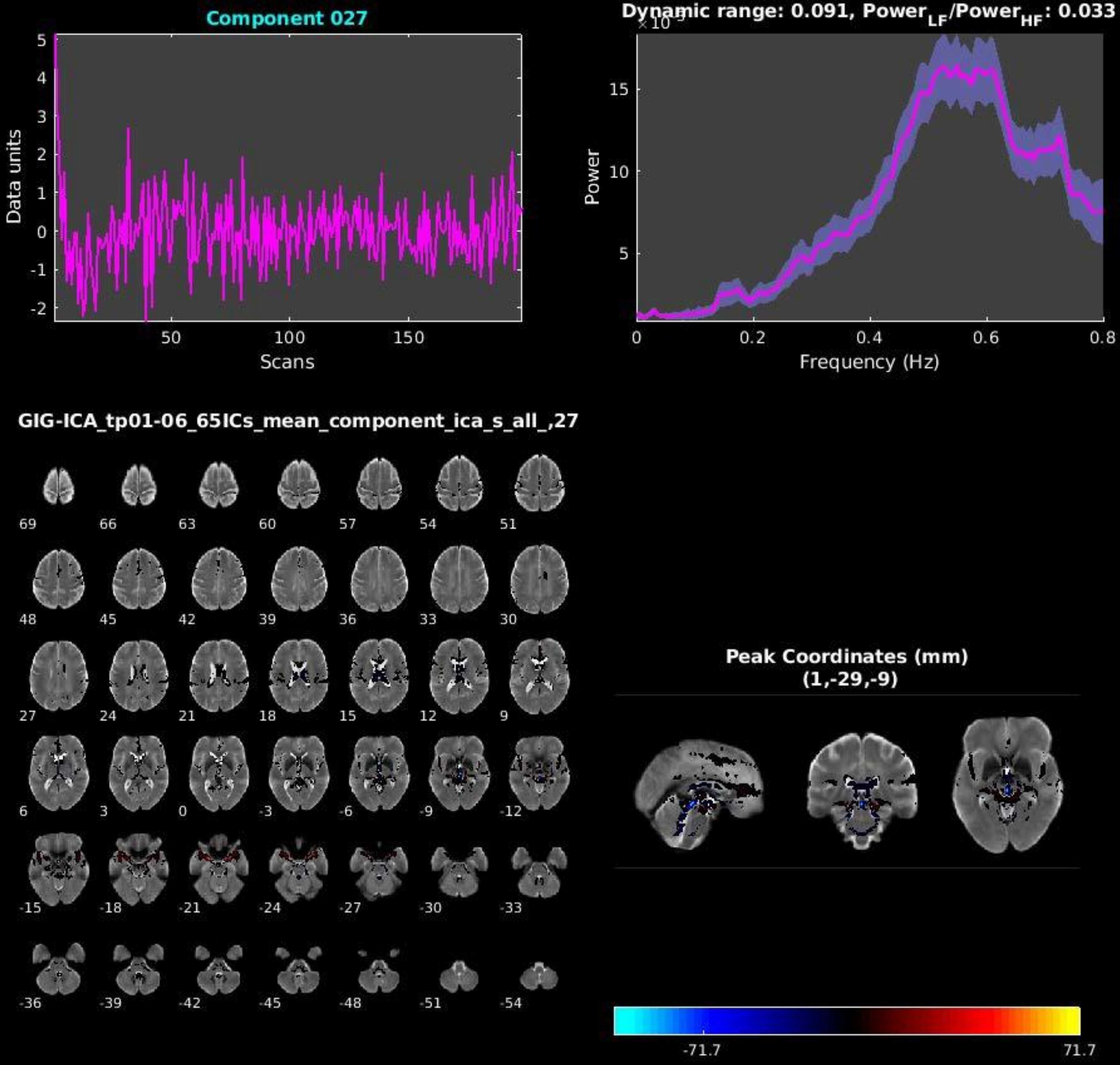
Component 027
Data units
Scans
Dynamic range: 0.091, Power_LF/Power_HF: 0.033
Power
Frequency (Hz)
GIG-ICA_tp01-06_65ICs_mean_component_ica_s_all_,27
Peak Coordinates (mm)
(1,-29,-9)
-71.7
71.7

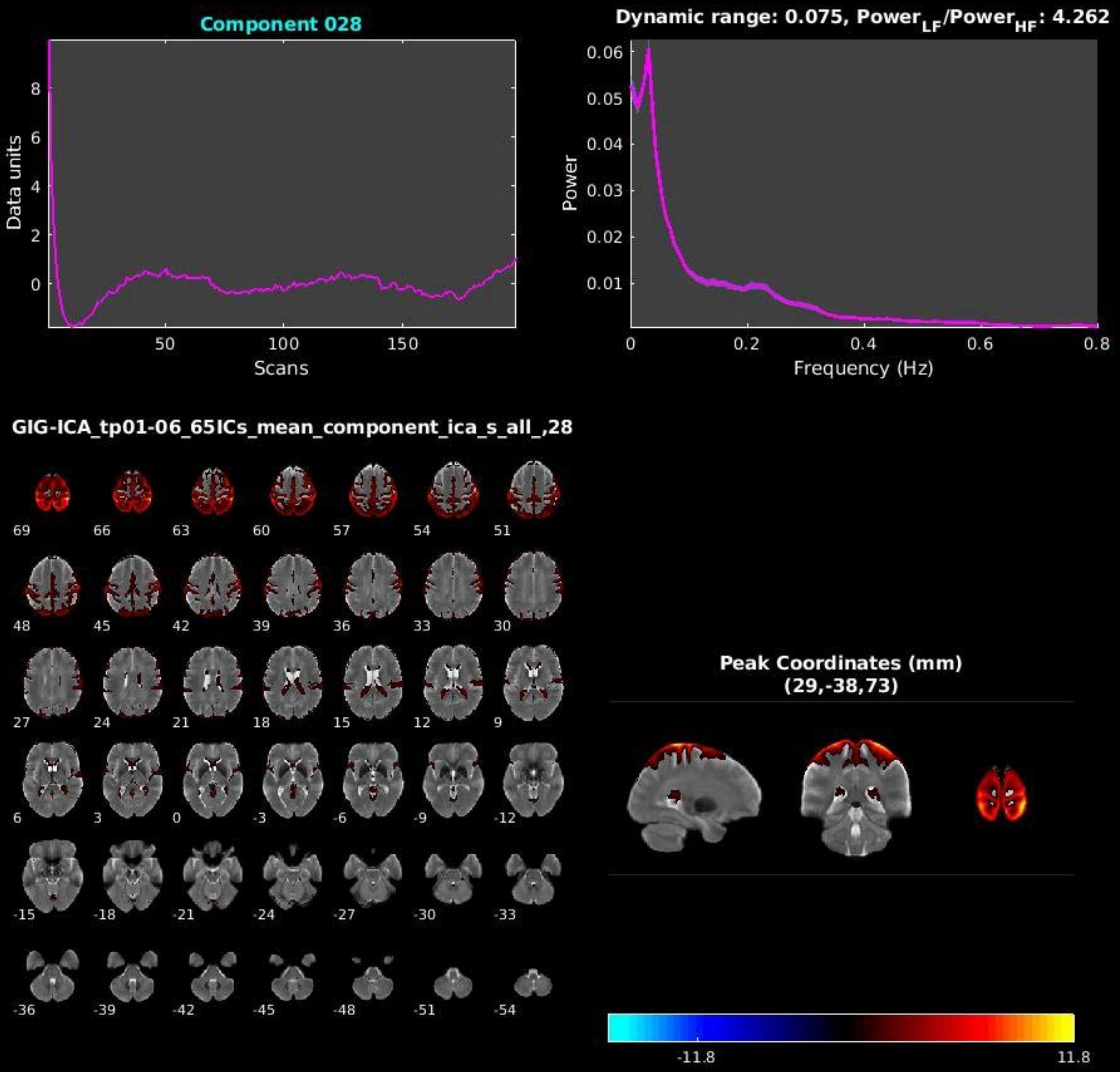
Component 028
Data units
Scans
Dynamic range: 0.075, Power_LF/Power_HF: 4.262
Power
Frequency (Hz)
GIG-ICA_tp01-06_65ICs_mean_component_ica_s_all_,28
69 66 63 60 57 54 51
48 45 42 39 36 33 30
27 24 21 18 15 12 9
6 3 0 -3 -6 -9 -12
-15 -18 -21 -24 -27 -30 -33
-36 -39 -42 -45 -48 -51 -54
Peak Coordinates (mm)
(29,-38,73)
-11.8
11.8

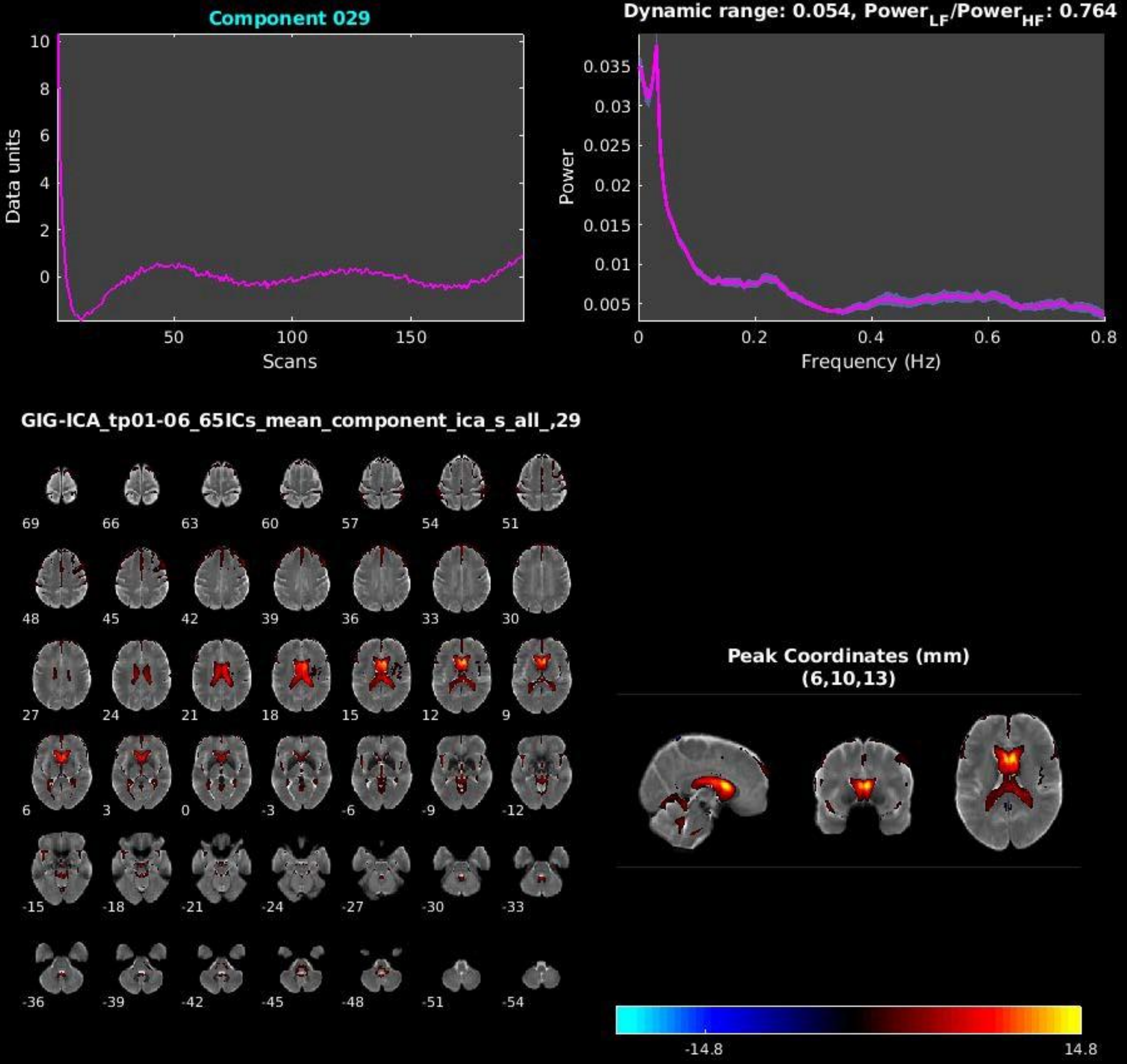
Component 029
Data units
Scans
Dynamic range: 0.054, Power$_{LF}$/Power$_{HF}$: 0.764
Power
Frequency (Hz)
GIG-ICA_tp01-06_65ICs_mean_component_ica_s_all_,29
69 66 63 60 57 54 51
48 45 42 39 36 33 30
27 24 21 18 15 12 9
6 3 0 -3 -6 -9 -12
-15 -18 -21 -24 -27 -30 -33
-36 -39 -42 -45 -48 -51 -54
Peak Coordinates (mm)
(6,10,13)
-14.8
14.8

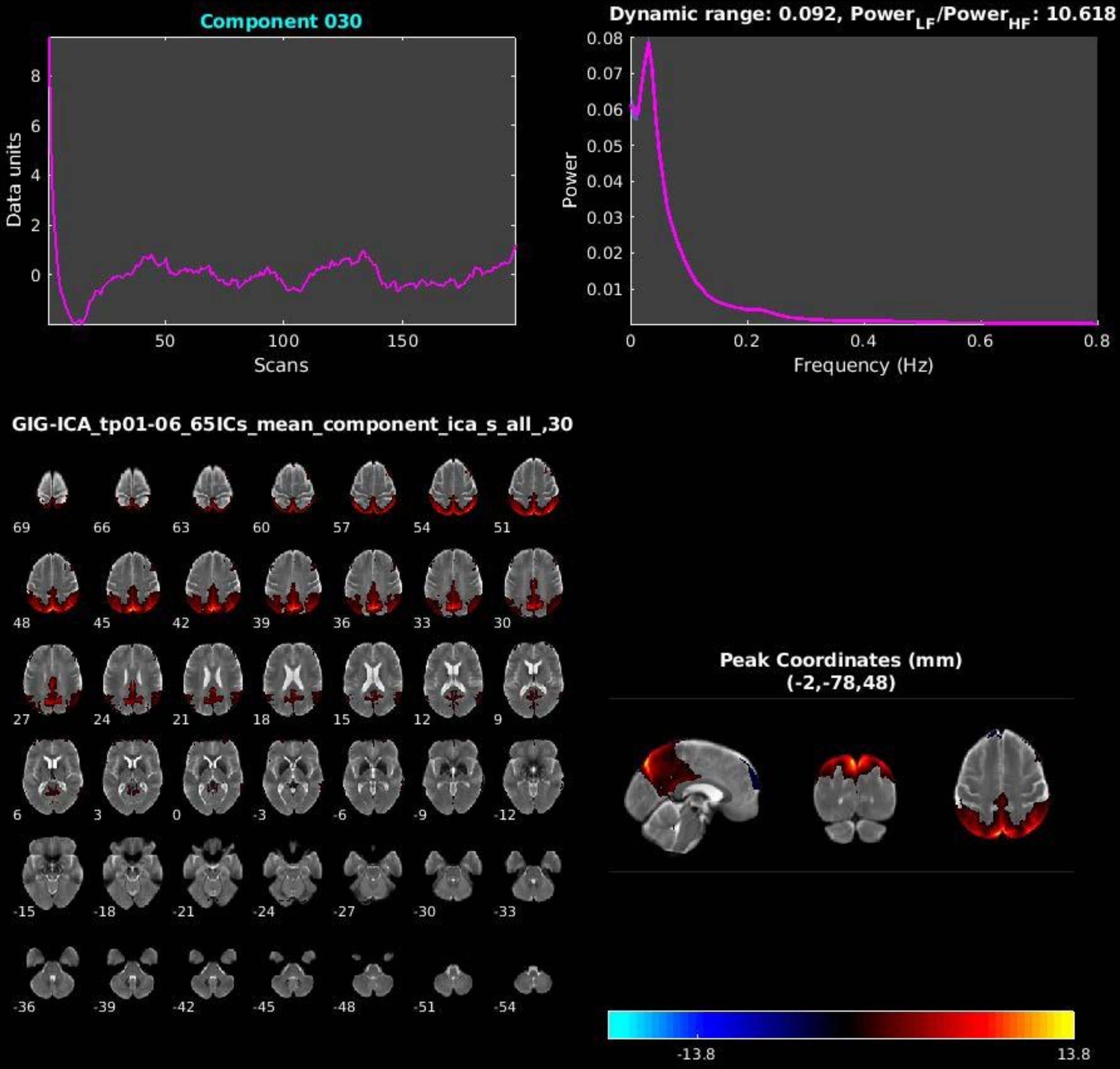

Component 030
Data units
Scans
Dynamic range: 0.092, $Power_{LF}/Power_{HF}$: 10.618
Power
Frequency (Hz)
GIG-ICA_tp01-06_65ICs_mean_component_ica_s_all_,30
Peak Coordinates (mm)
(-2,-78,48)
-13.8
13.8

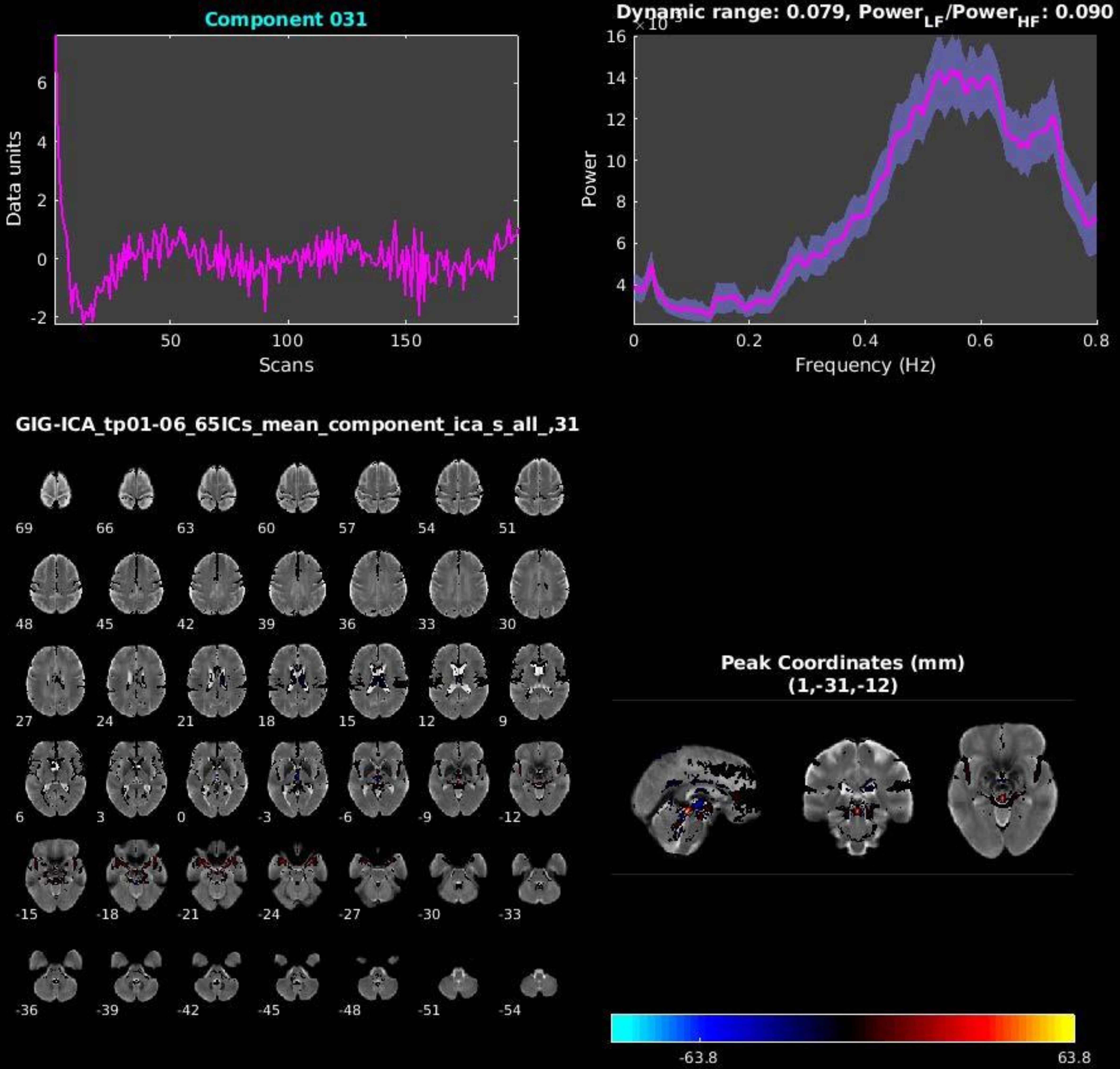

Component 031
Data units
Scans
Dynamic range: 0.079, Power$_{LF}$/Power$_{HF}$: 0.090
×10⁻³
Power
Frequency (Hz)
GIG-ICA_tp01-06_65ICs_mean_component_ica_s_all_,31
69 66 63 60 57 54 51
48 45 42 39 36 33 30
27 24 21 18 15 12 9
6 3 0 -3 -6 -9 -12
-15 -18 -21 -24 -27 -30 -33
-36 -39 -42 -45 -48 -51 -54
Peak Coordinates (mm)
(1,-31,-12)
-63.8
63.8

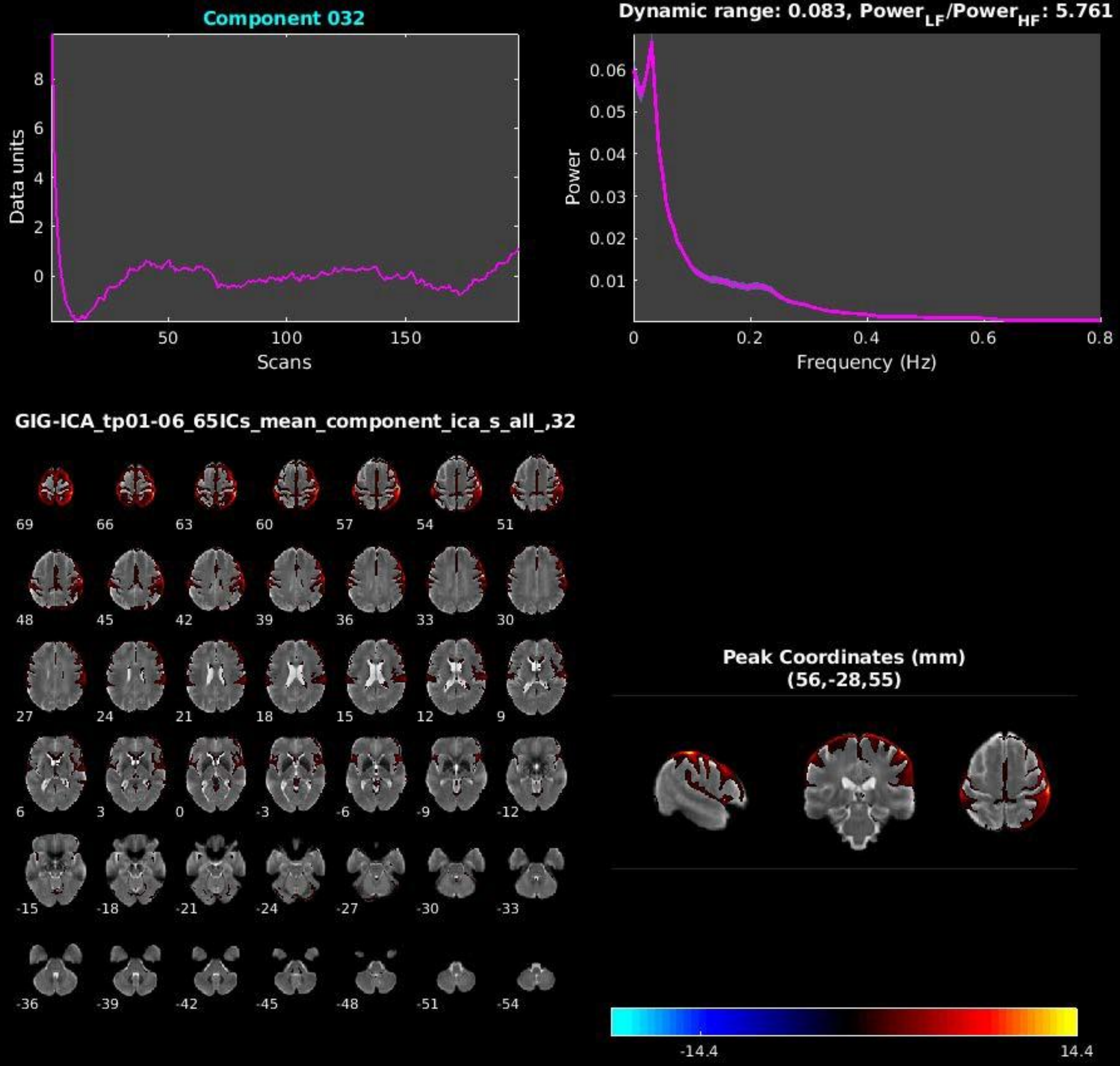
Component 032
Data units
Scans
0
2
4
6
8
50
100
150
Dynamic range: 0.083, Power$_{LF}$/Power$_{HF}$: 5.761
Power
Frequency (Hz)
0.01
0.02
0.03
0.04
0.05
0.06
0
0.2
0.4
0.6
0.8
GIG-ICA_tp01-06_65ICs_mean_component_ica_s_all_,32
69
66
63
60
57
54
51
48
45
42
39
36
33
30
27
24
21
18
15
12
9
6
3
0
-3
-6
-9
-12
-15
-18
-21
-24
-27
-30
-33
-36
-39
-42
-45
-48
-51
-54
Peak Coordinates (mm)
(56,-28,55)
-14.4
14.4

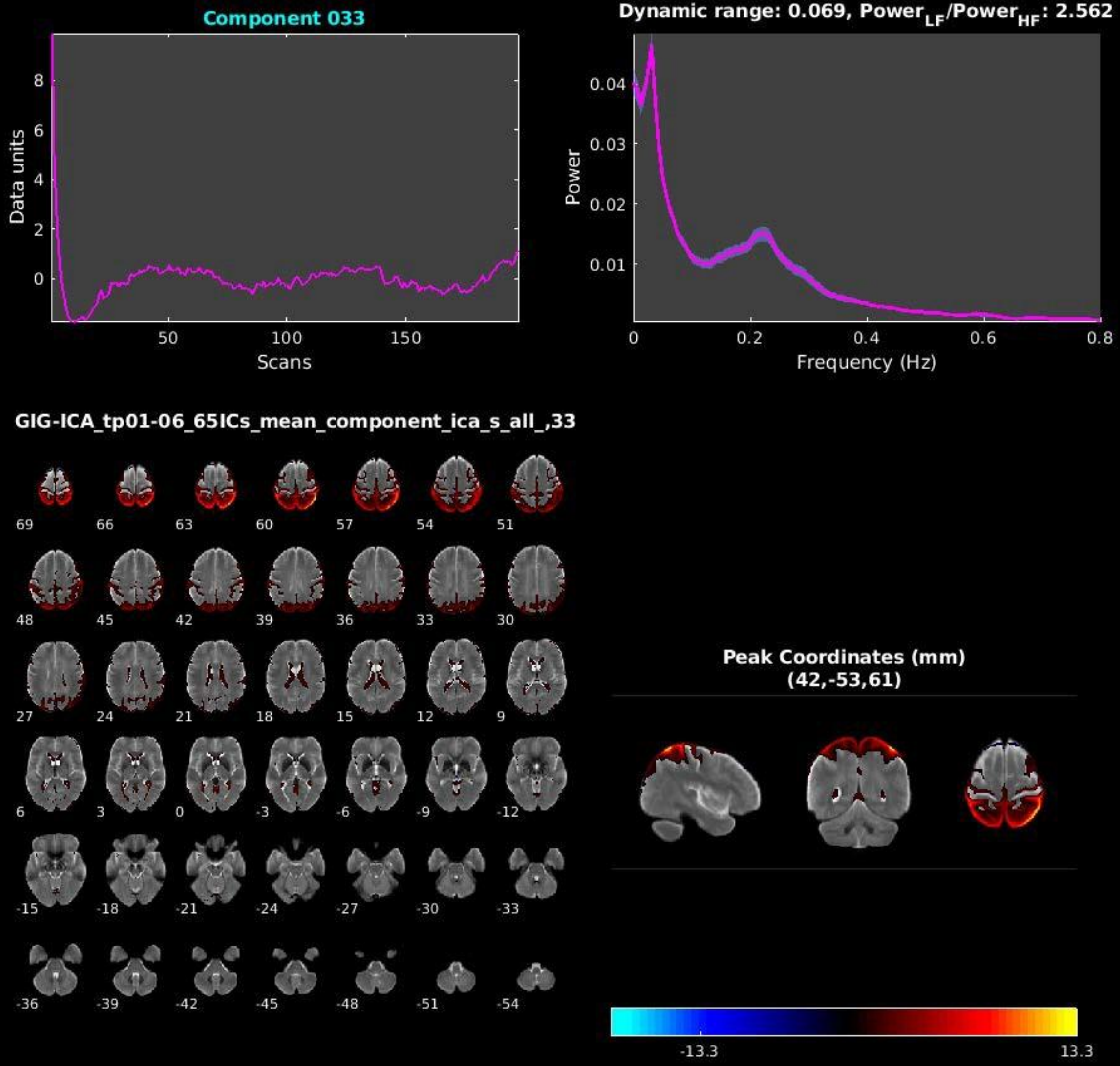

Component 033
Data units
Scans
Dynamic range: 0.069, Power_LF/Power_HF: 2.562
Power
Frequency (Hz)
GIG-ICA_tp01-06_65ICs_mean_component_ica_s_all_,33
Peak Coordinates (mm)
(42,-53,61)
-13.3
13.3

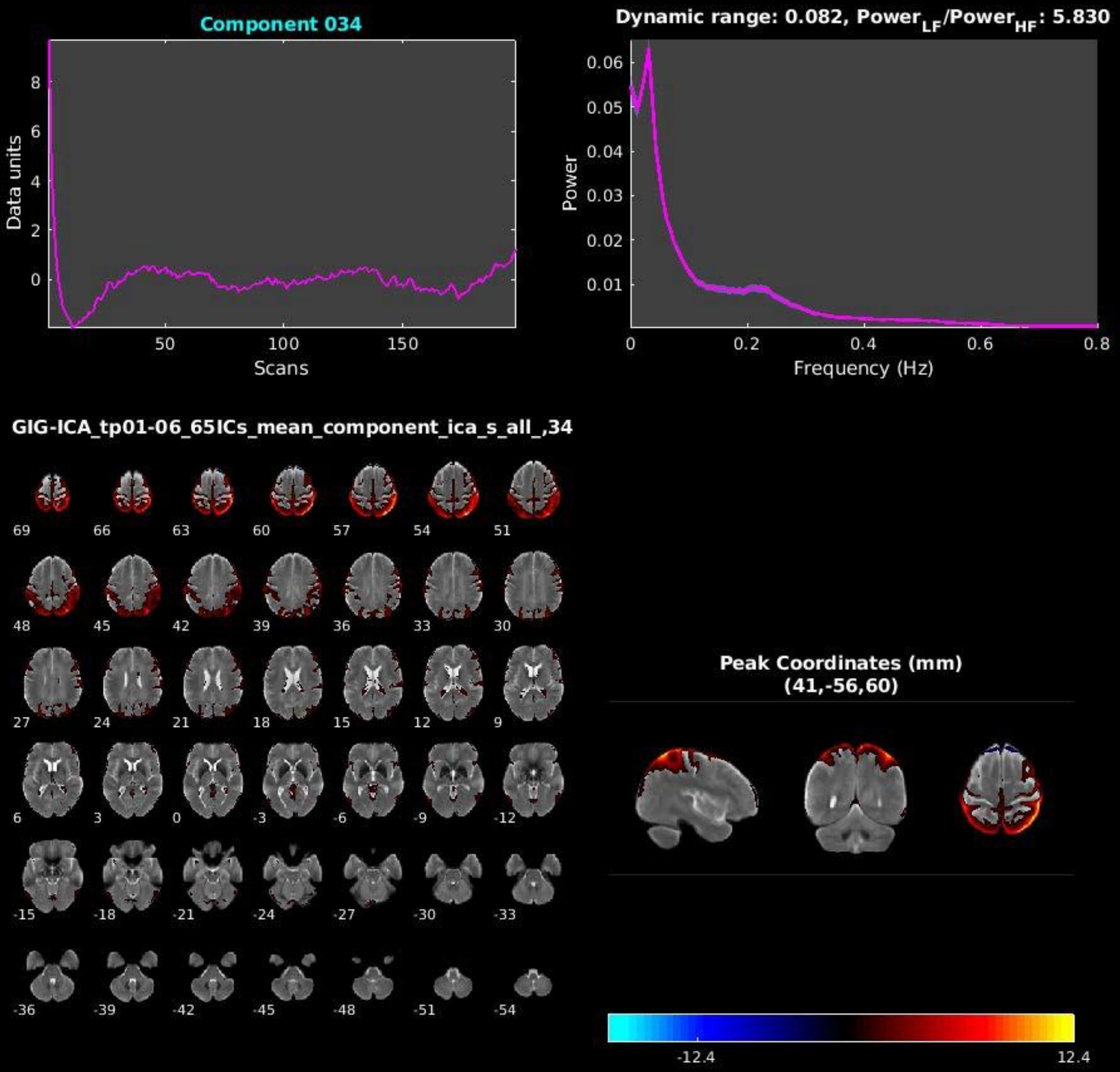

Component 034
Data units
Scans
Dynamic range: 0.082, Power_LF/Power_HF: 5.830
Power
Frequency (Hz)
GIG-ICA_tp01-06_65ICs_mean_component_ica_s_all_,34
69 66 63 60 57 54 51
48 45 42 39 36 33 30
27 24 21 18 15 12 9
6 3 0 -3 -6 -9 -12
-15 -18 -21 -24 -27 -30 -33
-36 -39 -42 -45 -48 -51 -54
Peak Coordinates (mm)
(41,-56,60)
-12.4
12.4

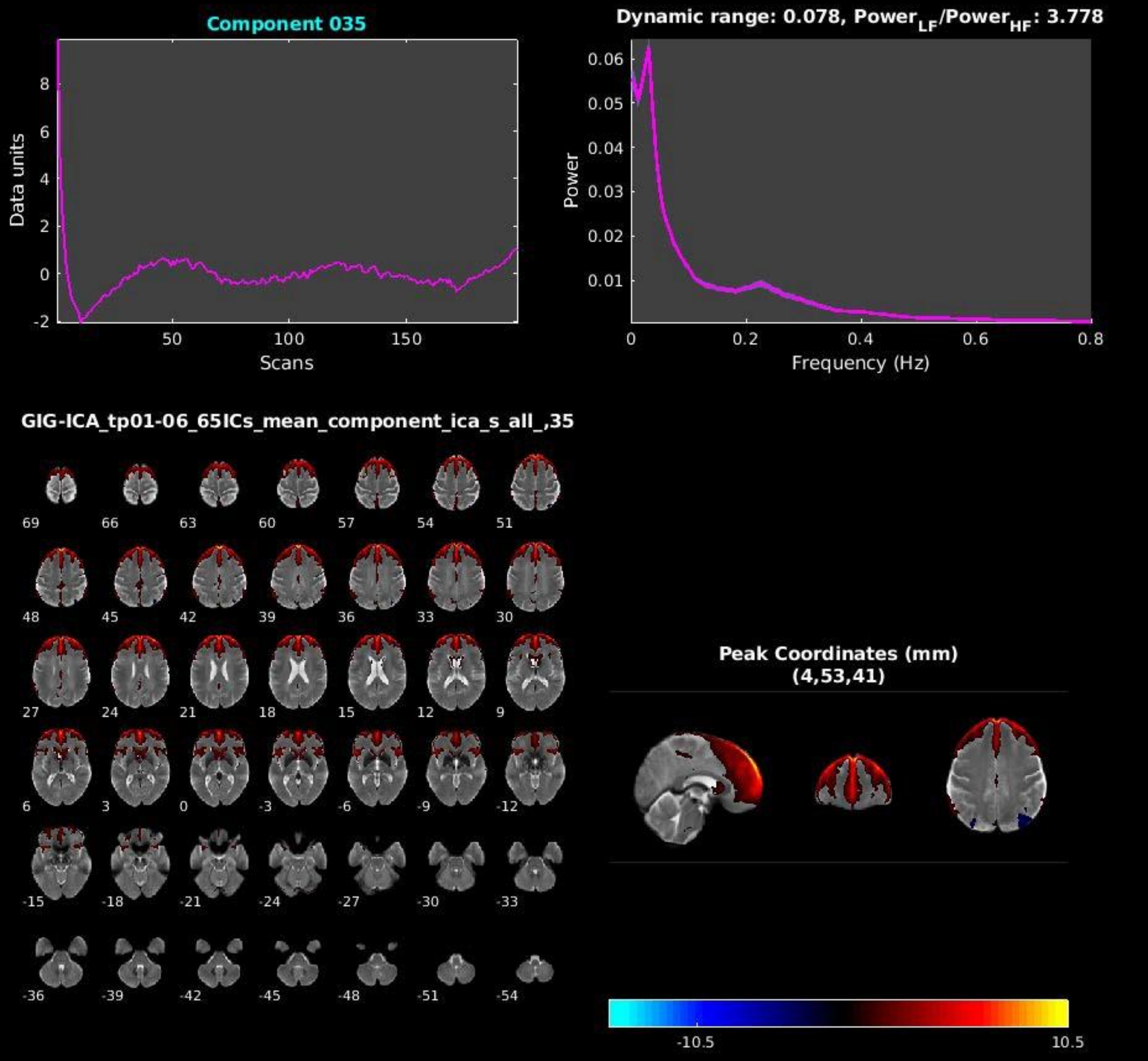
Component 035
Data units
Scans
Dynamic range: 0.078, Power_LF/Power_HF: 3.778
Power
Frequency (Hz)
GIG-ICA_tp01-06_65ICs_mean_component_ica_s_all_,35
Peak Coordinates (mm)
(4,53,41)
-10.5
10.5

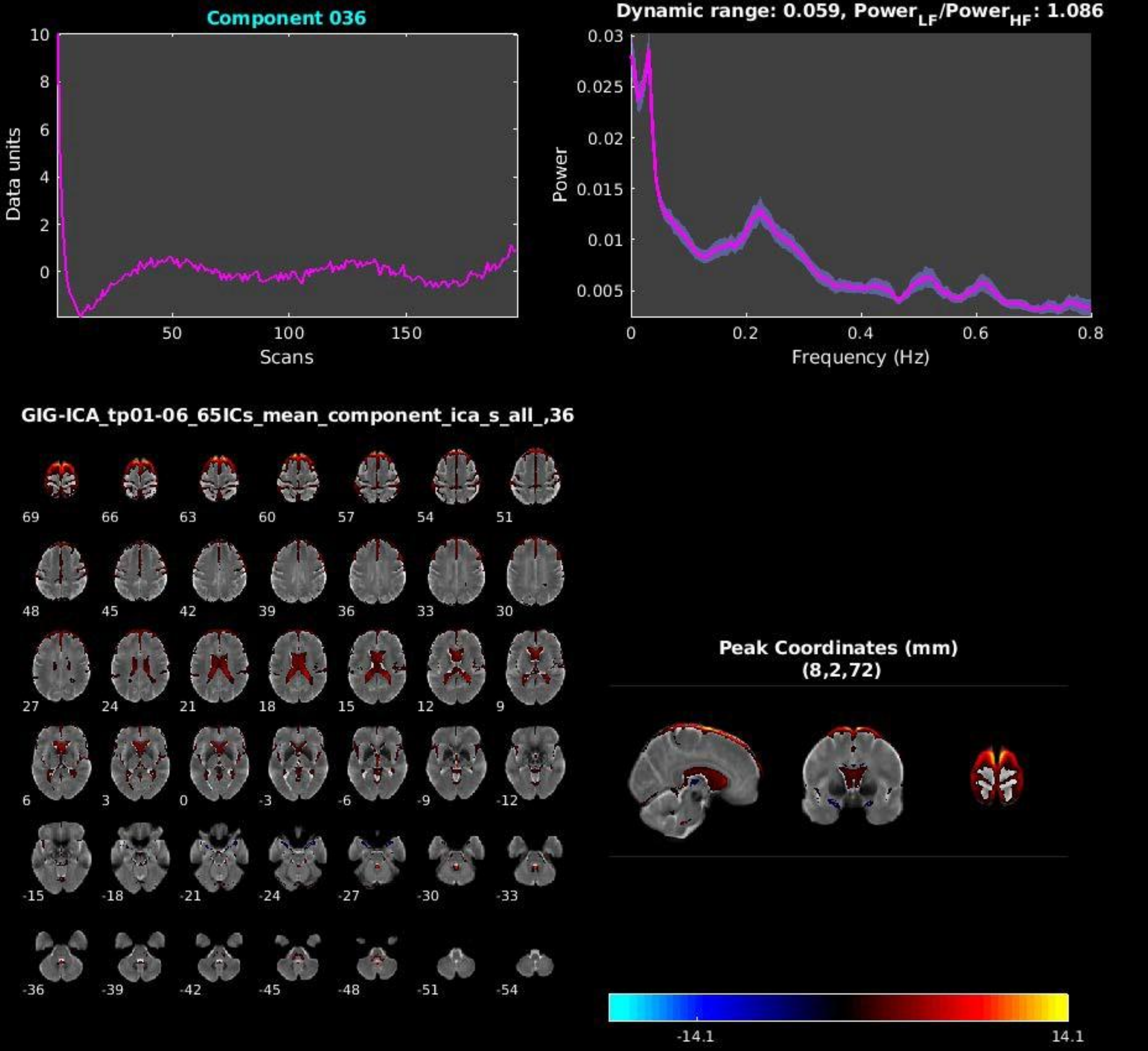

Component 036
Data units
Scans
Dynamic range: 0.059, Power$_{LF}$/Power$_{HF}$: 1.086
Power
Frequency (Hz)
GIG-ICA_tp01-06_65ICs_mean_component_ica_s_all_,36
Peak Coordinates (mm)
(8,2,72)
-14.1
14.1

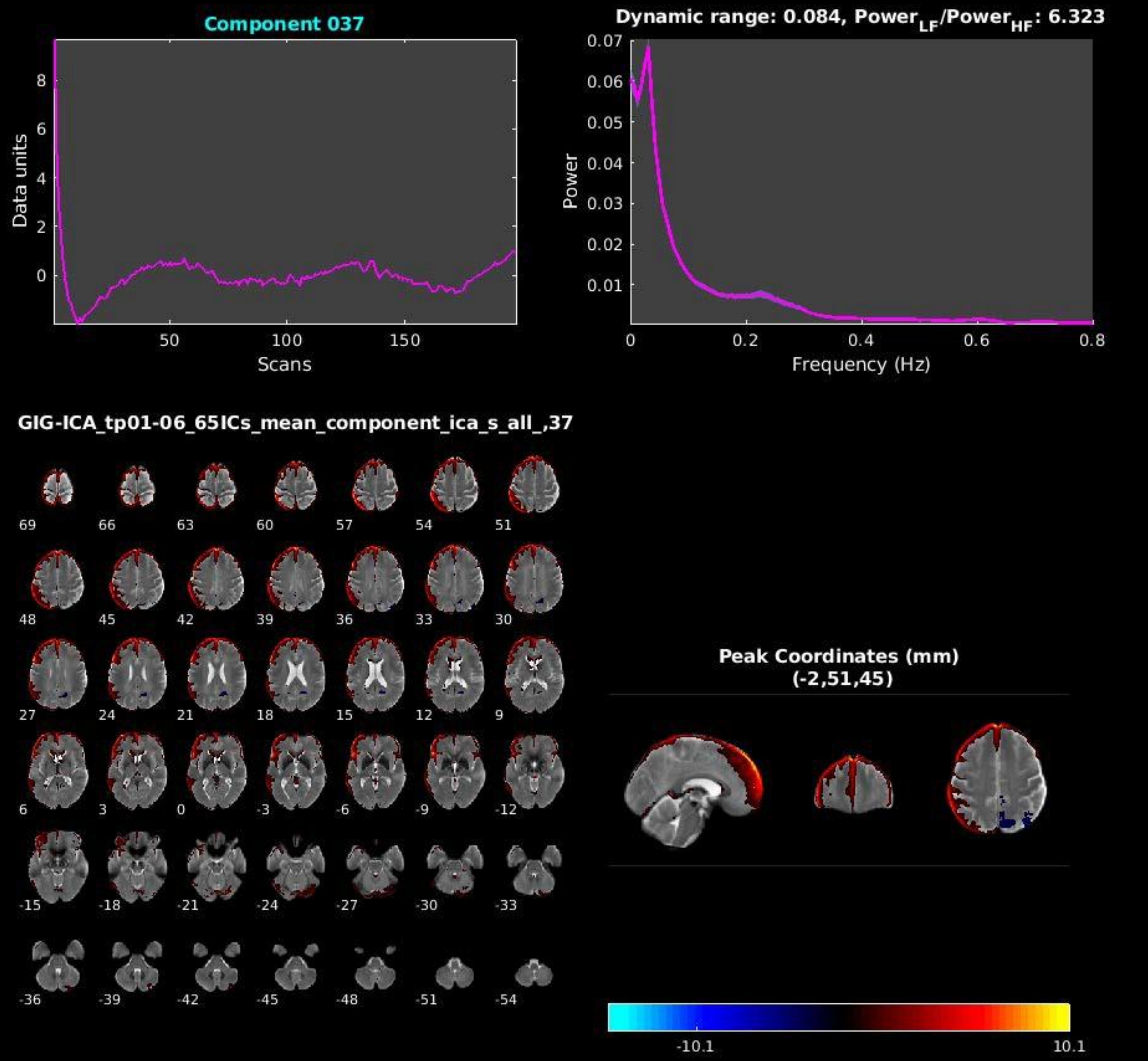

Component 037
Data units
Scans
Dynamic range: 0.084, Power_LF/Power_HF: 6.323
Power
Frequency (Hz)
GIG-ICA_tp01-06_65ICs_mean_component_ica_s_all_,37
Peak Coordinates (mm)
(-2,51,45)
-10.1
10.1

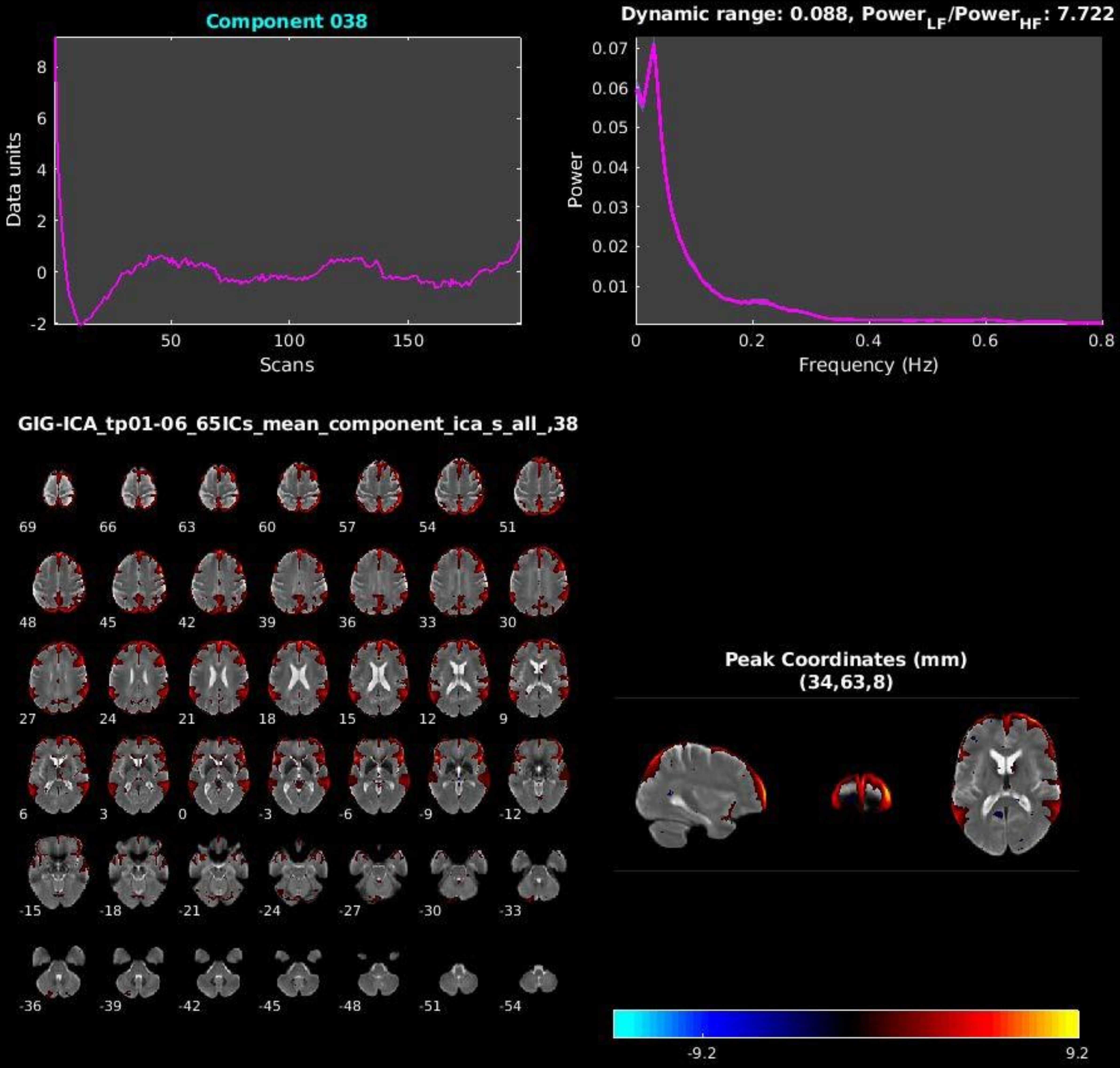
Component 038
Data units
Scans
Dynamic range: 0.088, Power$_{LF}$/Power$_{HF}$: 7.722
Power
Frequency (Hz)
GIG-ICA_tp01-06_65ICs_mean_component_ica_s_all_,38
Peak Coordinates (mm)
(34,63,8)
-9.2
9.2

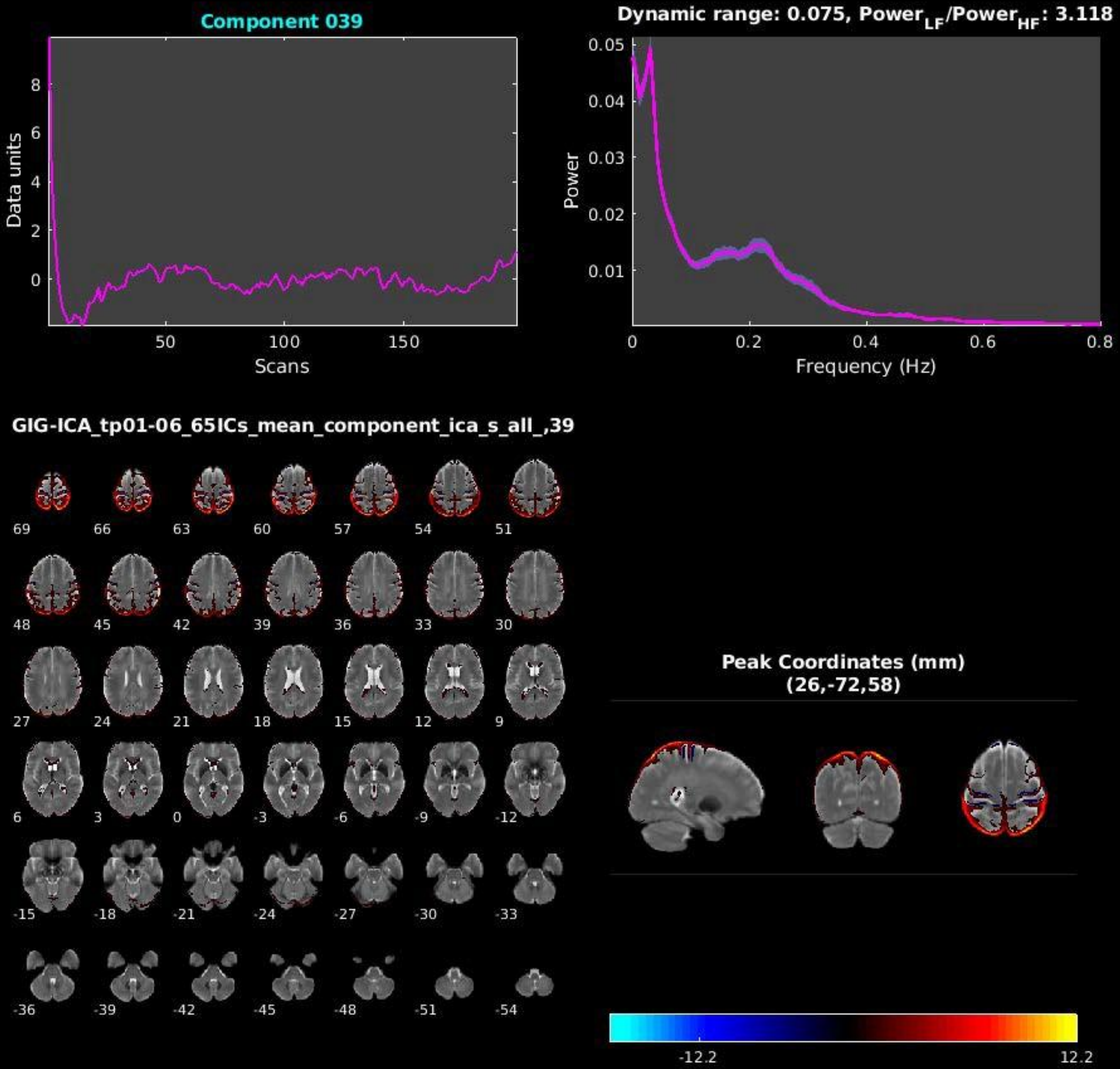
Component 039
Data units
Scans
Dynamic range: 0.075, Power$_{LF}$/Power$_{HF}$: 3.118
Power
Frequency (Hz)
GIG-ICA_tp01-06_65ICs_mean_component_ica_s_all_,39
Peak Coordinates (mm)
(26,-72,58)
-12.2
12.2

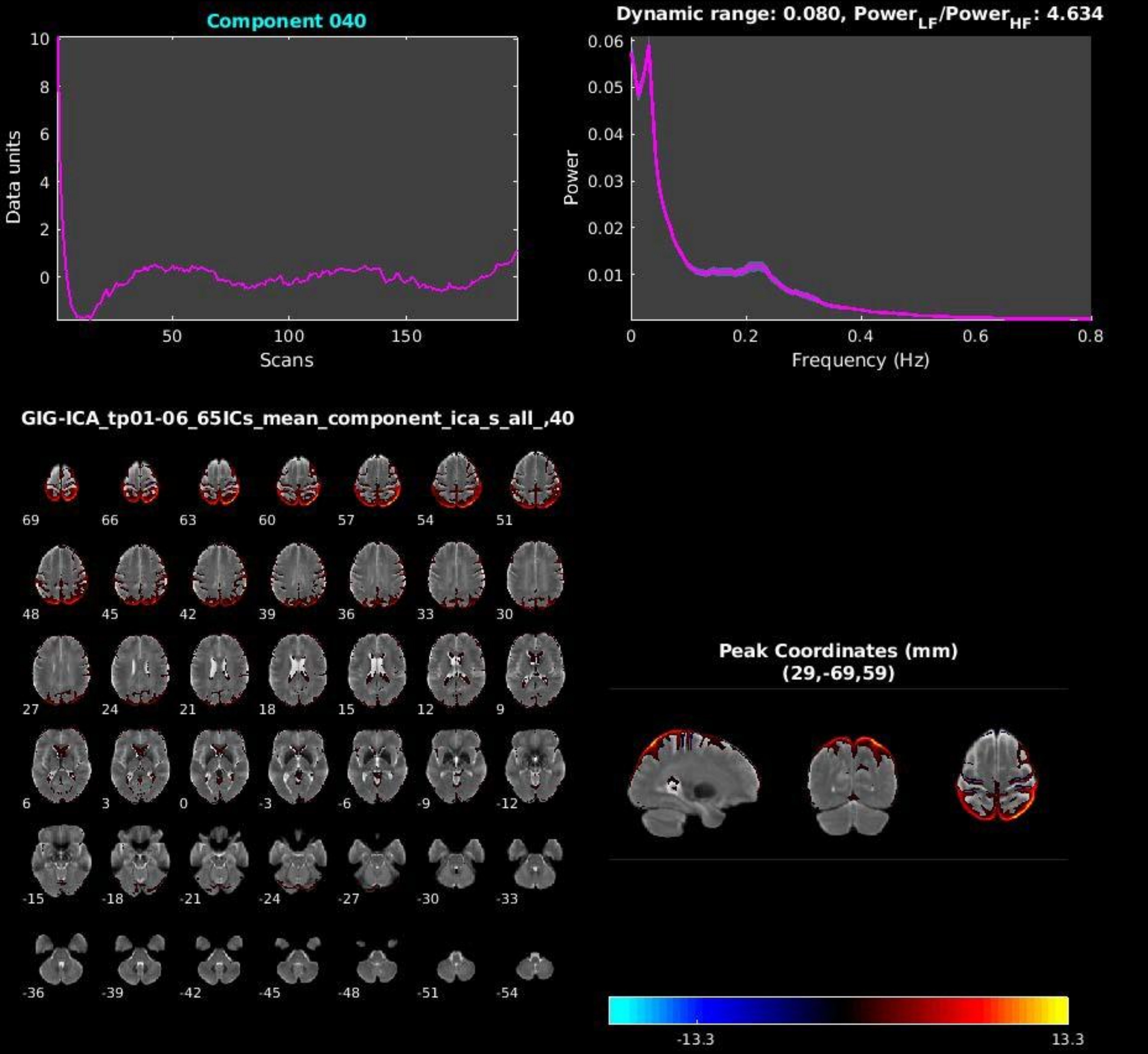
Component 040
Data units
Scans
Dynamic range: 0.080, Power_LF/Power_HF: 4.634
Power
Frequency (Hz)
GIG-ICA_tp01-06_65ICs_mean_component_ica_s_all_,40
Peak Coordinates (mm)
(29,-69,59)
-13.3
13.3

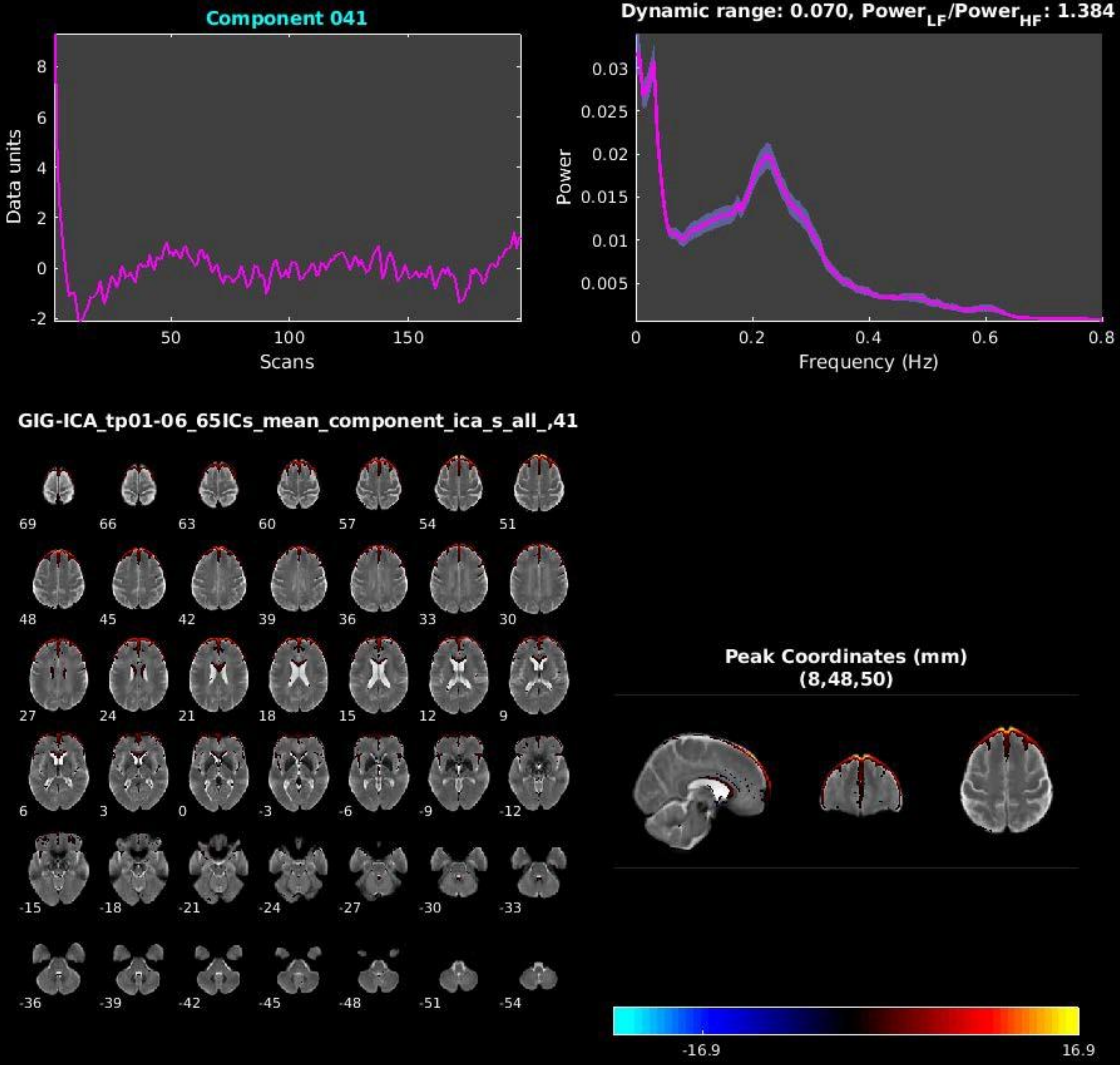

Component 041
Data units
Scans
8
6
4
2
0
-2
50
100
150
Dynamic range: 0.070, Power$_{LF}$/Power$_{HF}$: 1.384
Power
Frequency (Hz)
0.03
0.025
0.02
0.015
0.01
0.005
0
0.2
0.4
0.6
0.8
GIG-ICA_tp01-06_65ICs_mean_component_ica_s_all_,41
69
66
63
60
57
54
51
48
45
42
39
36
33
30
27
24
21
18
15
12
9
6
3
0
-3
-6
-9
-12
-15
-18
-21
-24
-27
-30
-33
-36
-39
-42
-45
-48
-51
-54
Peak Coordinates (mm)
(8,48,50)
-16.9
16.9

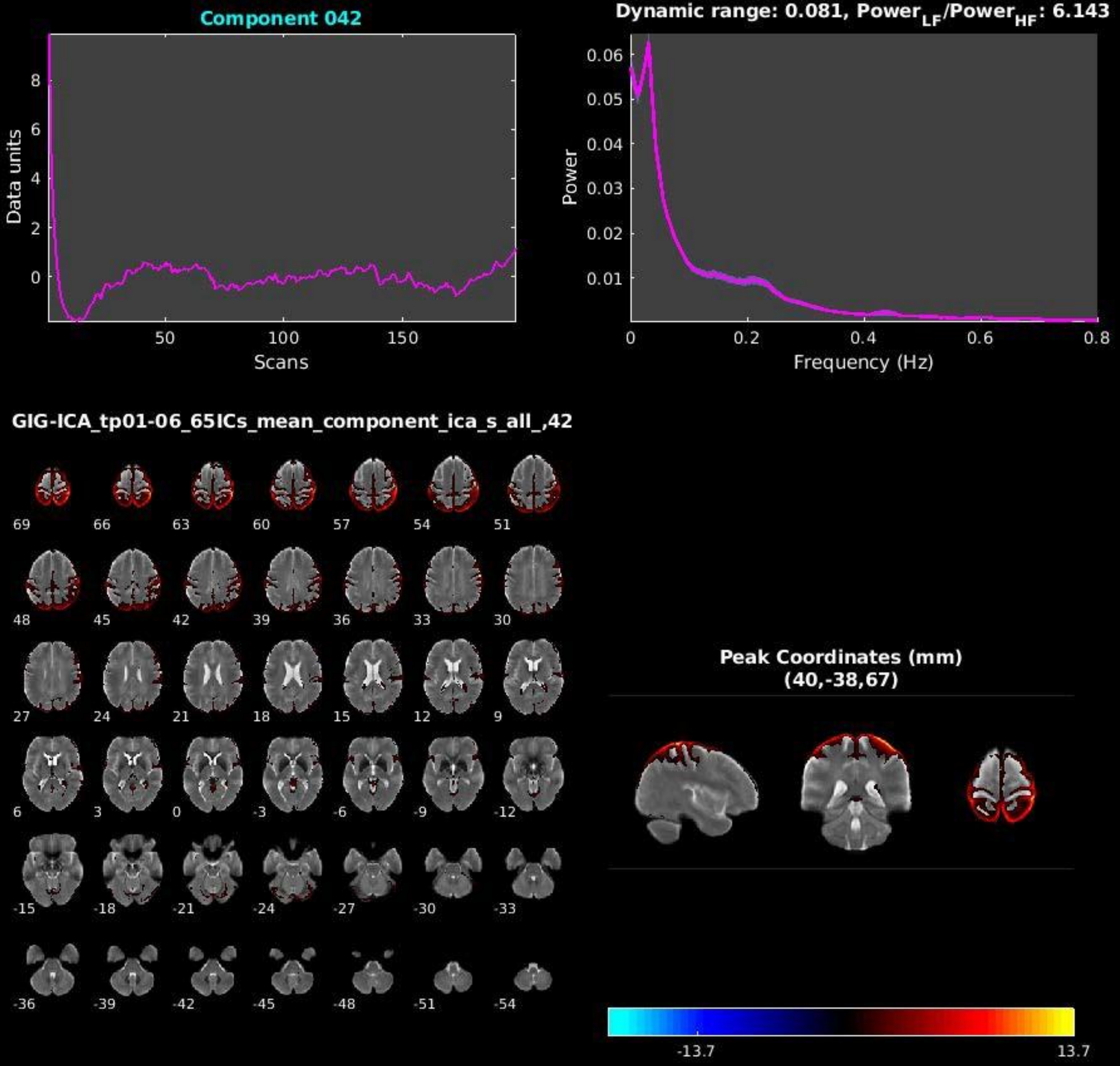

Component 042
Data units
Scans
Dynamic range: 0.081, Power_LF/Power_HF: 6.143
Power
Frequency (Hz)
GIG-ICA_tp01-06_65ICs_mean_component_ica_s_all_,42
Peak Coordinates (mm)
(40,-38,67)
-13.7
13.7

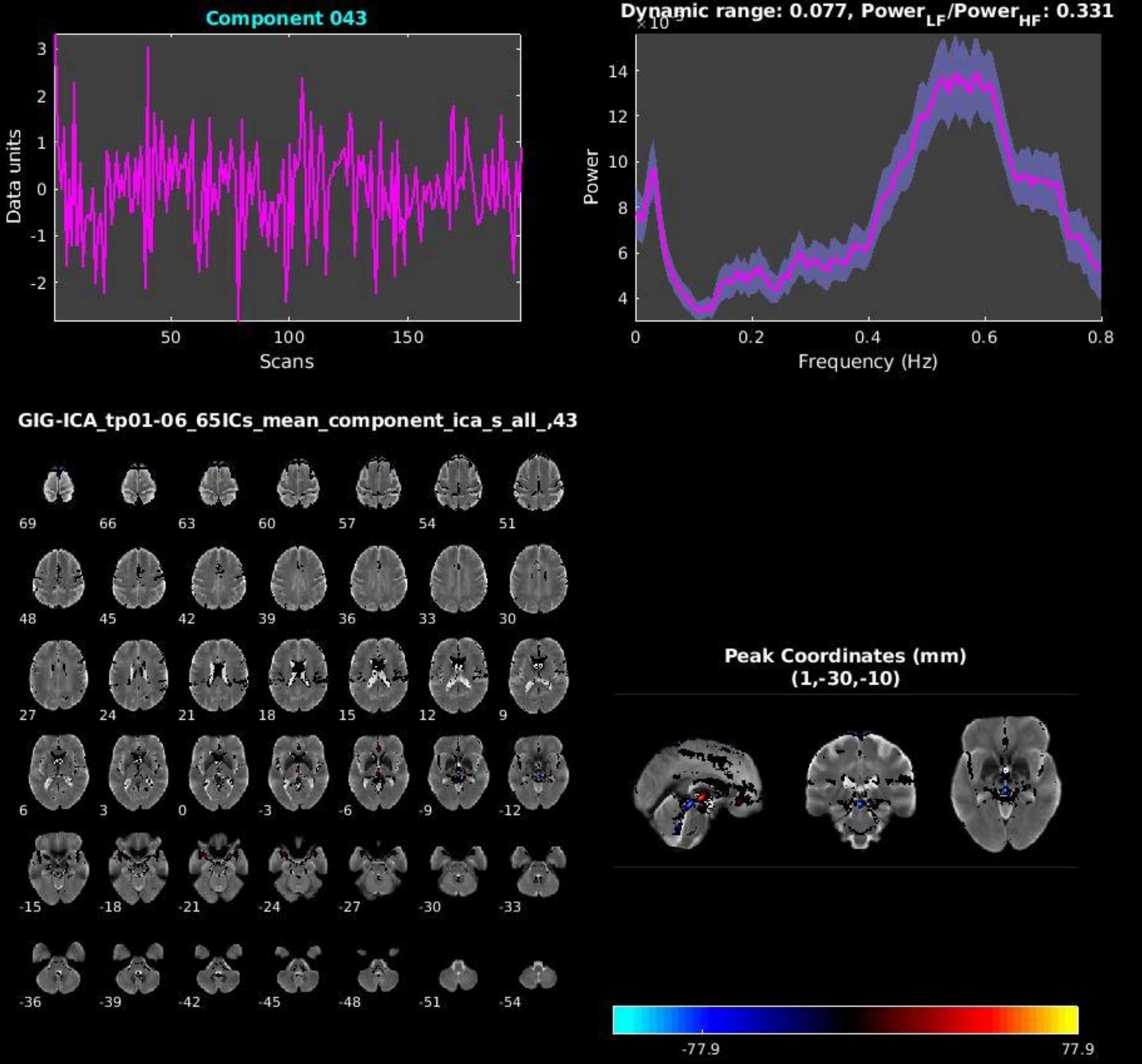
Component 043
Data units
Scans
Dynamic range: 0.077, Power$_{LF}$/Power$_{HF}$: 0.331
Power
Frequency (Hz)
GIG-ICA_tp01-06_65ICs_mean_component_ica_s_all_,43
Peak Coordinates (mm)
(1,-30,-10)
-77.9
77.9

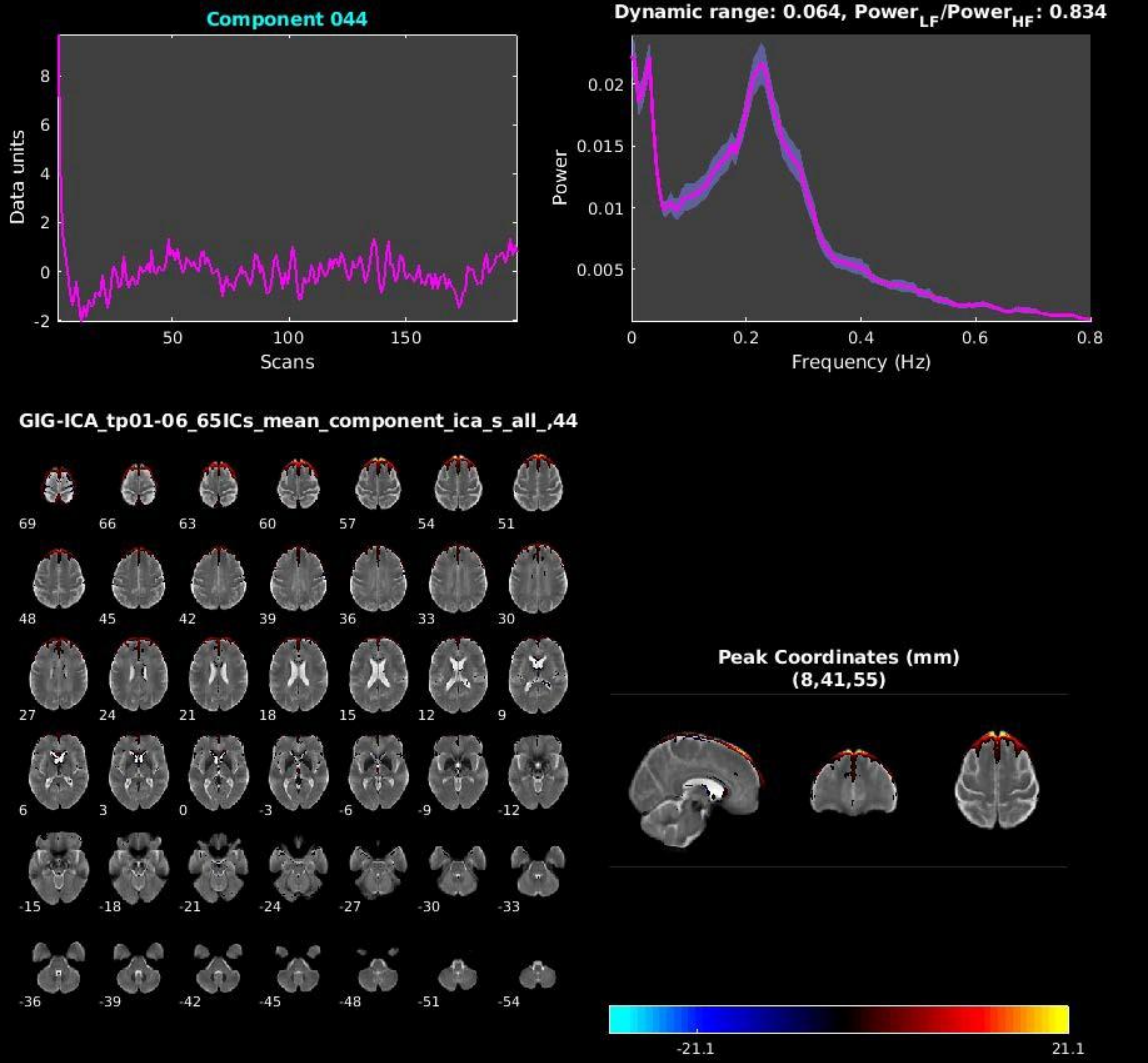
Component 044
Data units
Scans
Dynamic range: 0.064, Power_LF/Power_HF: 0.834
Power
Frequency (Hz)
GIG-ICA_tp01-06_65ICs_mean_component_ica_s_all_,44
Peak Coordinates (mm)
(8,41,55)
-21.1
21.1

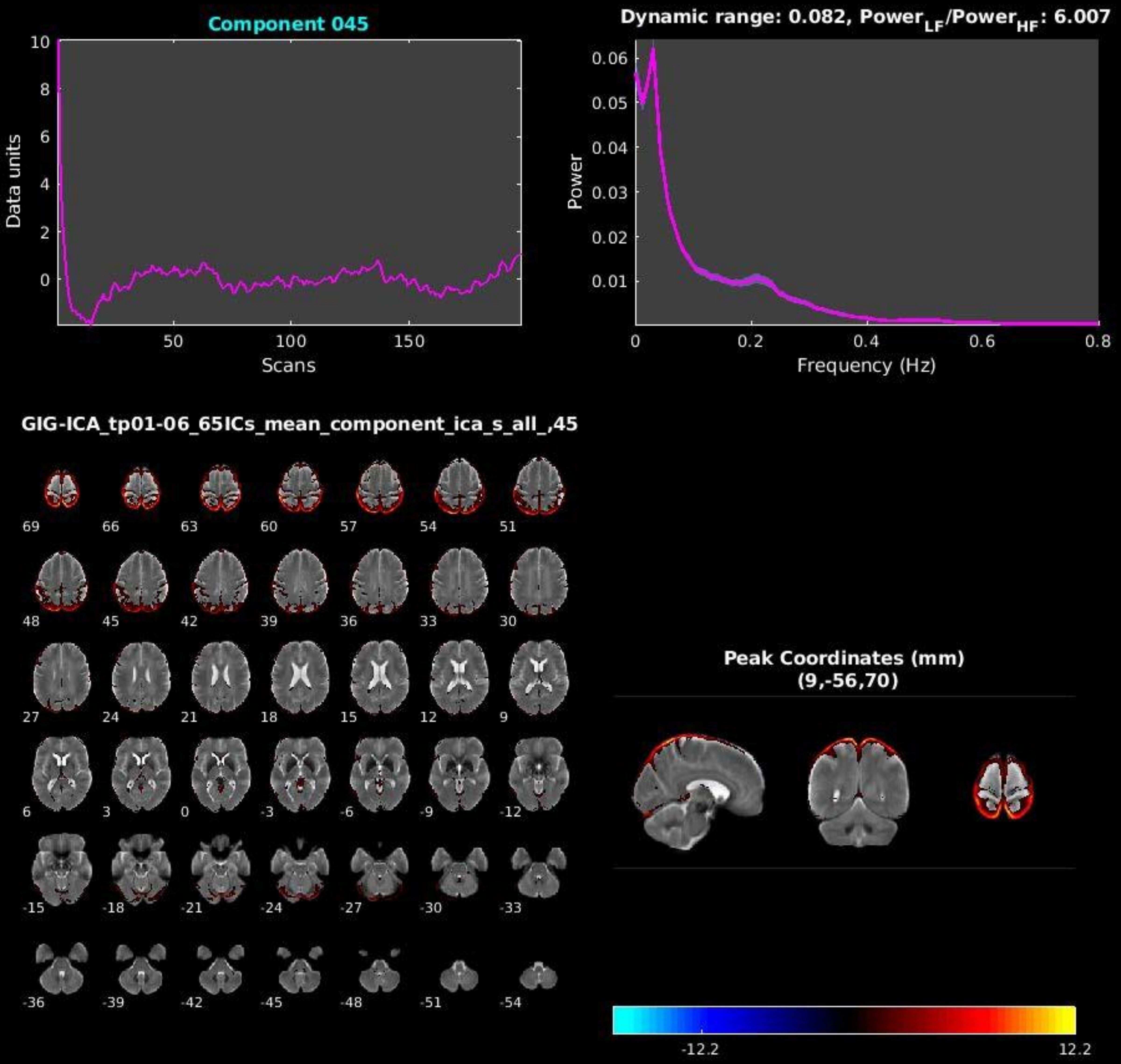
Component 045
Data units
Scans
Dynamic range: 0.082, Power_LF/Power_HF: 6.007
Power
Frequency (Hz)
GIG-ICA_tp01-06_65ICs_mean_component_ica_s_all_,45
69 66 63 60 57 54 51
48 45 42 39 36 33 30
27 24 21 18 15 12 9
6 3 0 -3 -6 -9 -12
-15 -18 -21 -24 -27 -30 -33
-36 -39 -42 -45 -48 -51 -54
Peak Coordinates (mm)
(9,-56,70)
-12.2
12.2

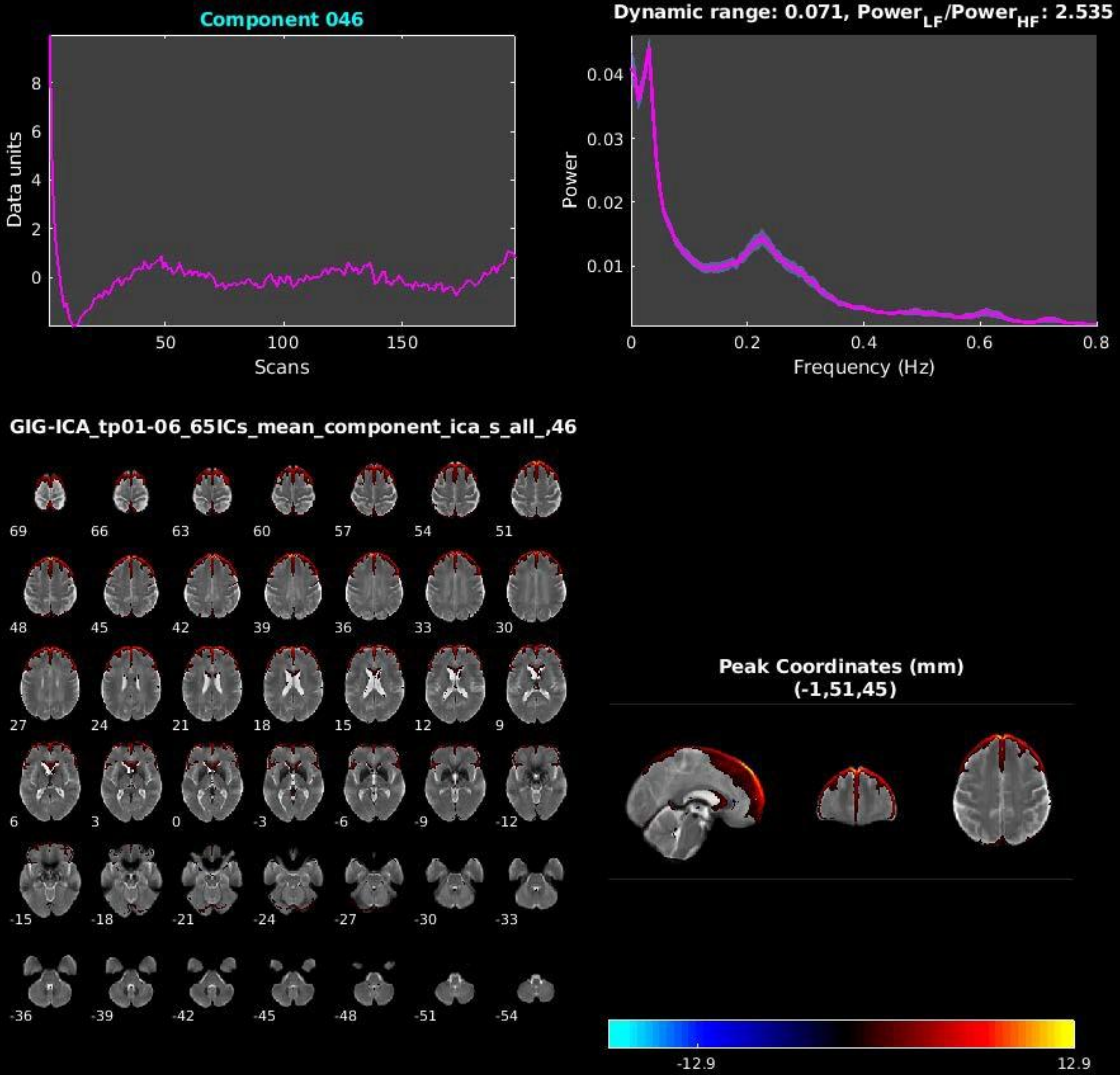
Component 046
Dynamic range: 0.071, Power$_{LF}$/Power$_{HF}$: 2.535
Data units
Scans
Power
Frequency (Hz)
GIG-ICA_tp01-06_65ICs_mean_component_ica_s_all_,46
Peak Coordinates (mm)
(-1,51,45)
-12.9
12.9

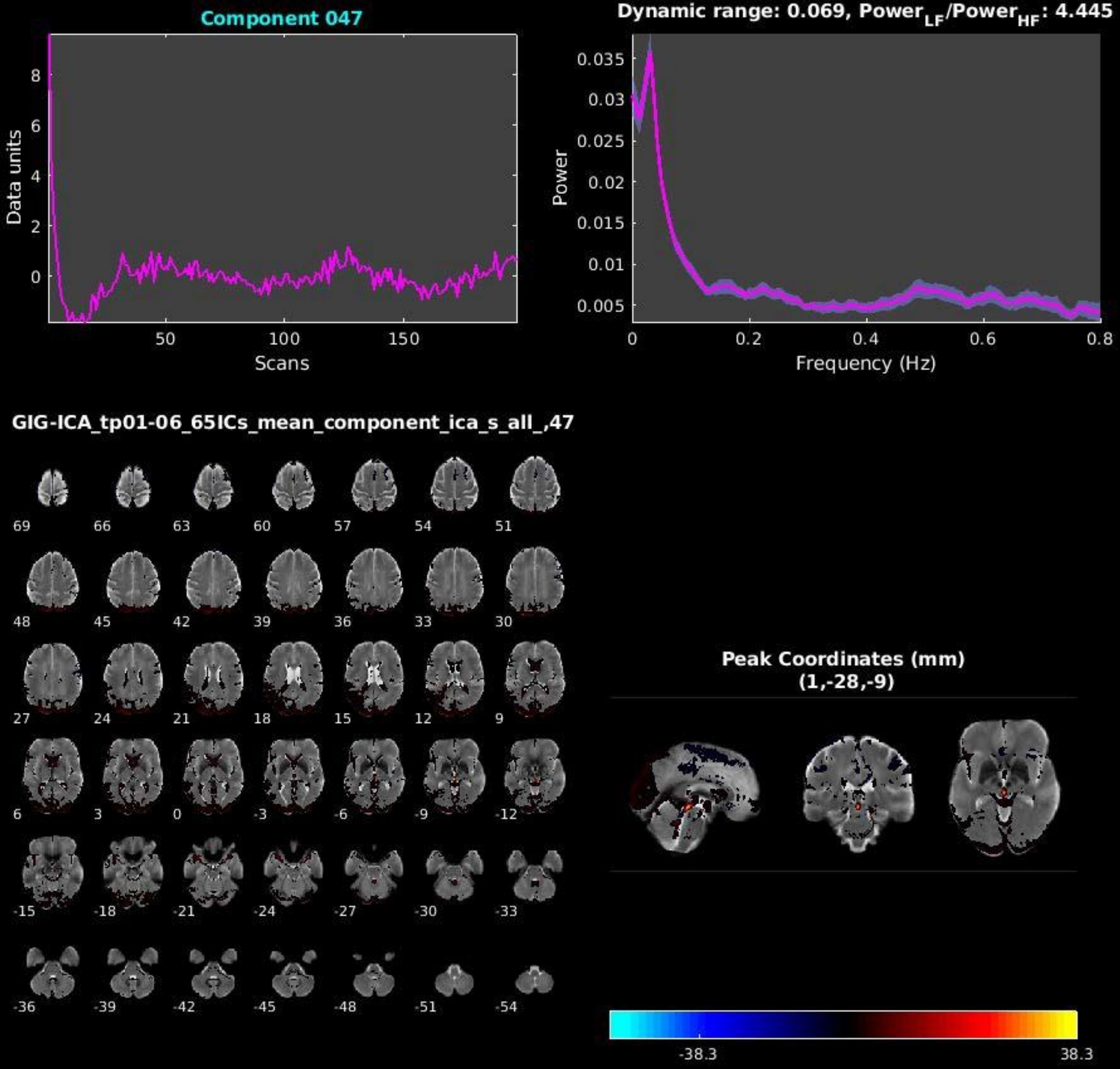

Component 047
Data units
Scans
Dynamic range: 0.069, $Power_{LF}/Power_{HF}$: 4.445
Power
Frequency (Hz)
GIG-ICA_tp01-06_65ICs_mean_component_ica_s_all_,47
Peak Coordinates (mm)
(1,-28,-9)
-38.3
38.3

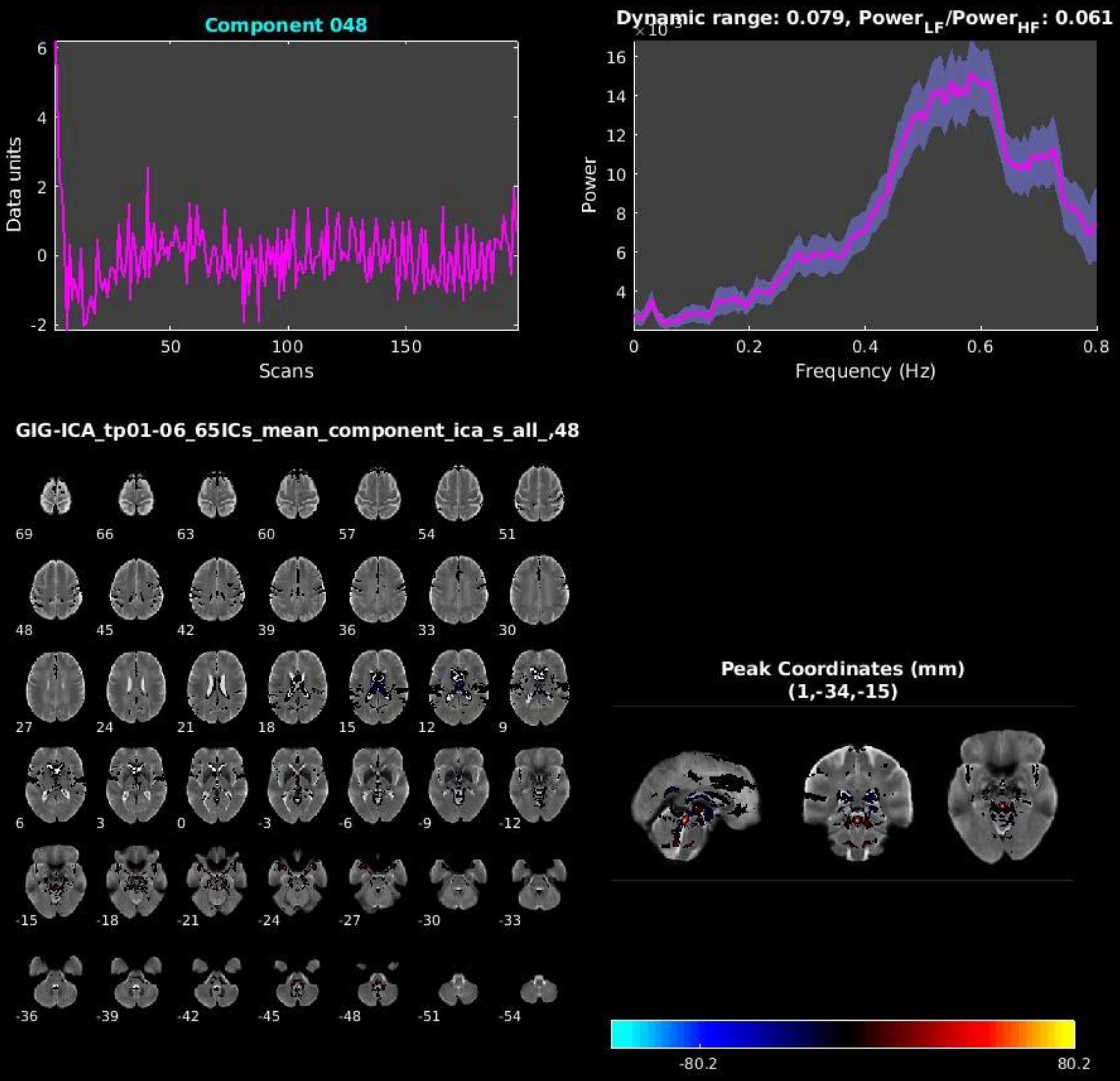
Component 048
Data units
Scans
Dynamic range: 0.079, Power_LF/Power_HF: 0.061
Power
Frequency (Hz)
GIG-ICA_tp01-06_65ICs_mean_component_ica_s_all_,48
Peak Coordinates (mm)
(1,-34,-15)
-80.2
80.2

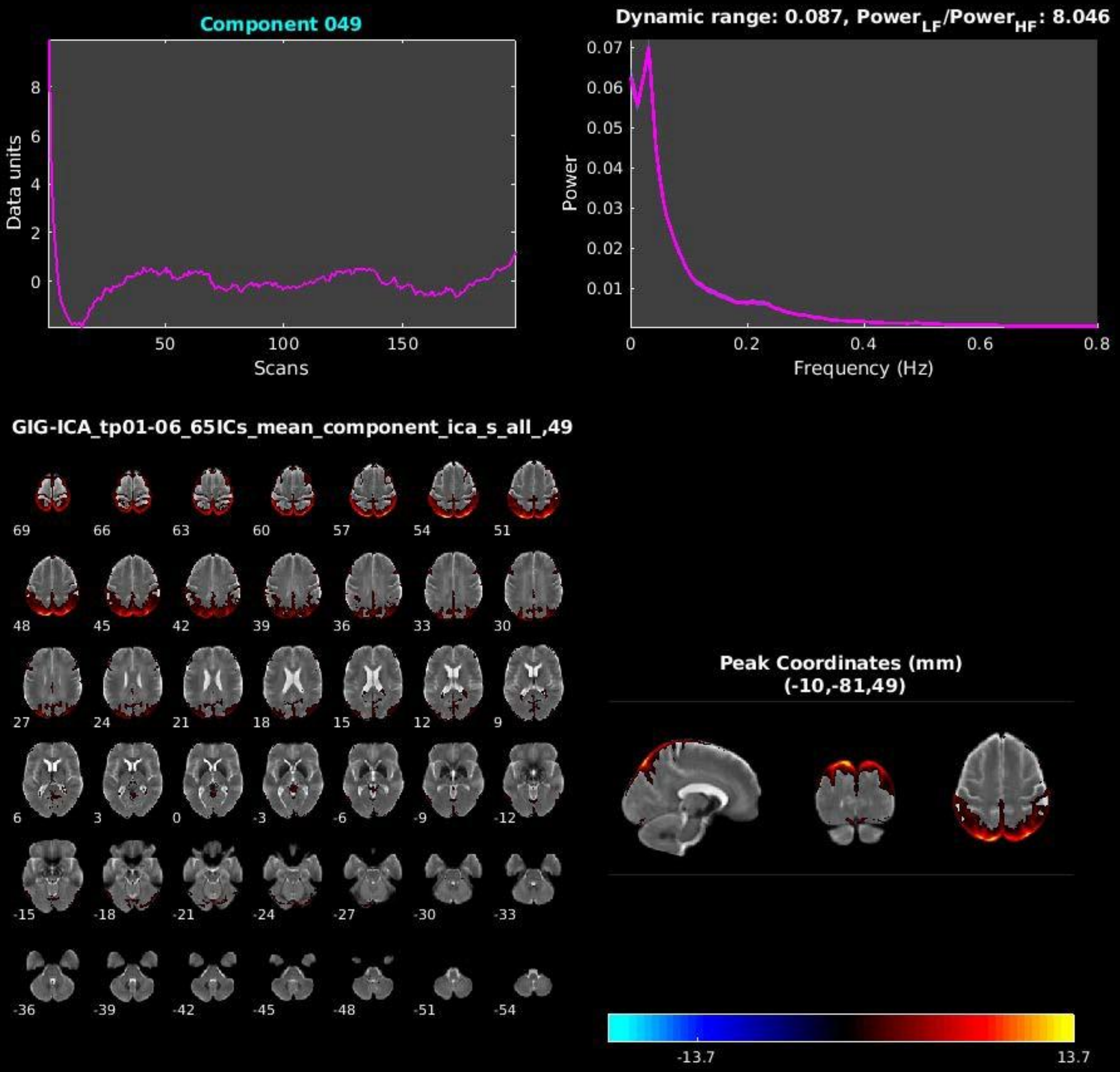

Component 049
Data units
Scans
Dynamic range: 0.087, Power$_{LF}$/Power$_{HF}$: 8.046
Power
Frequency (Hz)
GIG-ICA_tp01-06_65ICs_mean_component_ica_s_all_,49
Peak Coordinates (mm)
(-10,-81,49)
-13.7
13.7

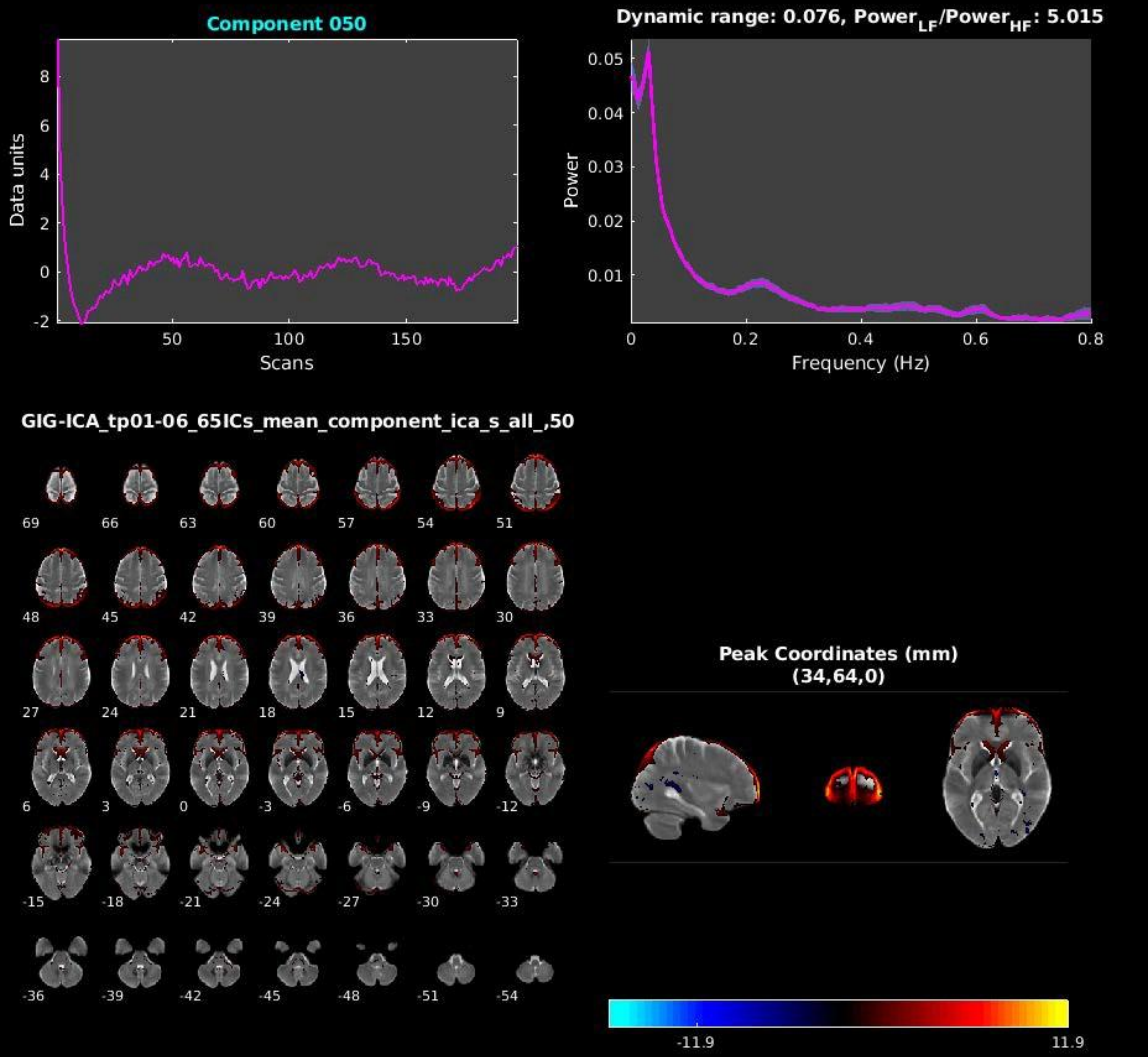
Component 050
Data units
Scans
Dynamic range: 0.076, Power$_{LF}$/Power$_{HF}$: 5.015
Power
Frequency (Hz)
GIG-ICA_tp01-06_65ICs_mean_component_ica_s_all_,50
Peak Coordinates (mm)
(34,64,0)
-11.9
11.9

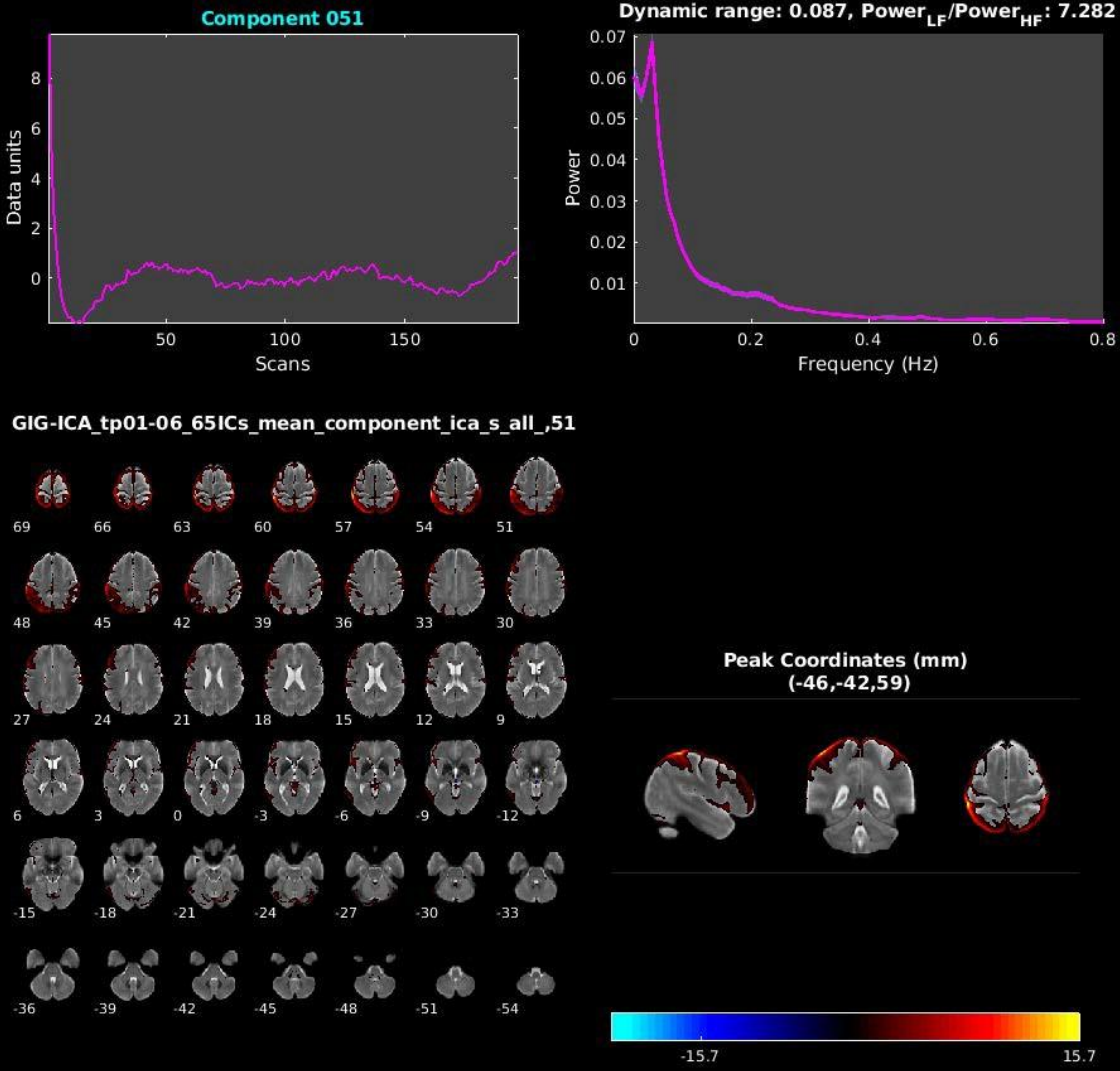
Component 051
Data units
Scans
Dynamic range: 0.087, Power_LF/Power_HF: 7.282
Power
Frequency (Hz)
GIG-ICA_tp01-06_65ICs_mean_component_ica_s_all_,51
69 66 63 60 57 54 51
48 45 42 39 36 33 30
27 24 21 18 15 12 9
6 3 0 -3 -6 -9 -12
-15 -18 -21 -24 -27 -30 -33
-36 -39 -42 -45 -48 -51 -54
Peak Coordinates (mm)
(-46,-42,59)
-15.7
15.7

SUPPLEMENTARY DATA

Component 052

Dynamic range: 0.066, PowerLF/PowerHF: 0.475

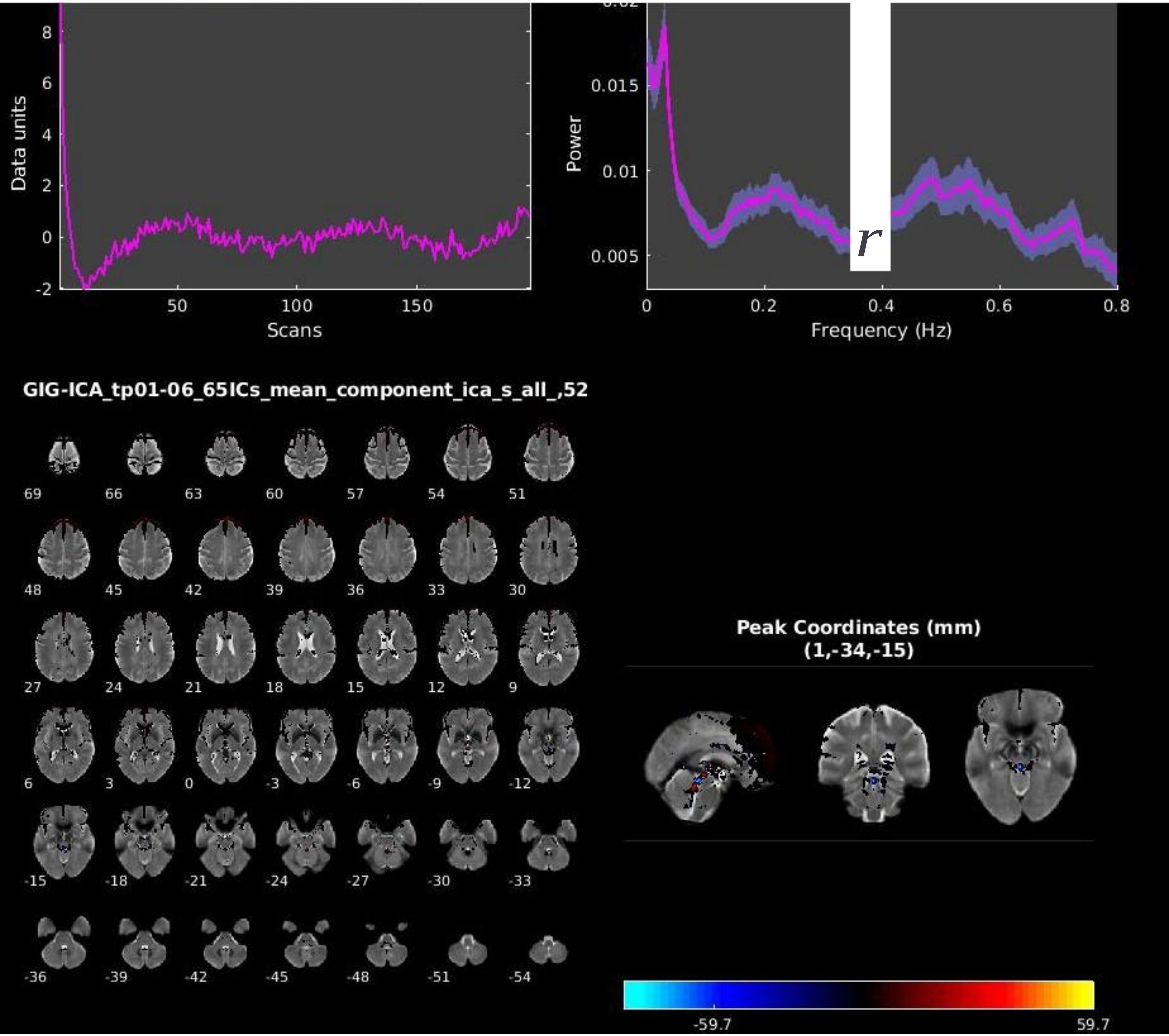

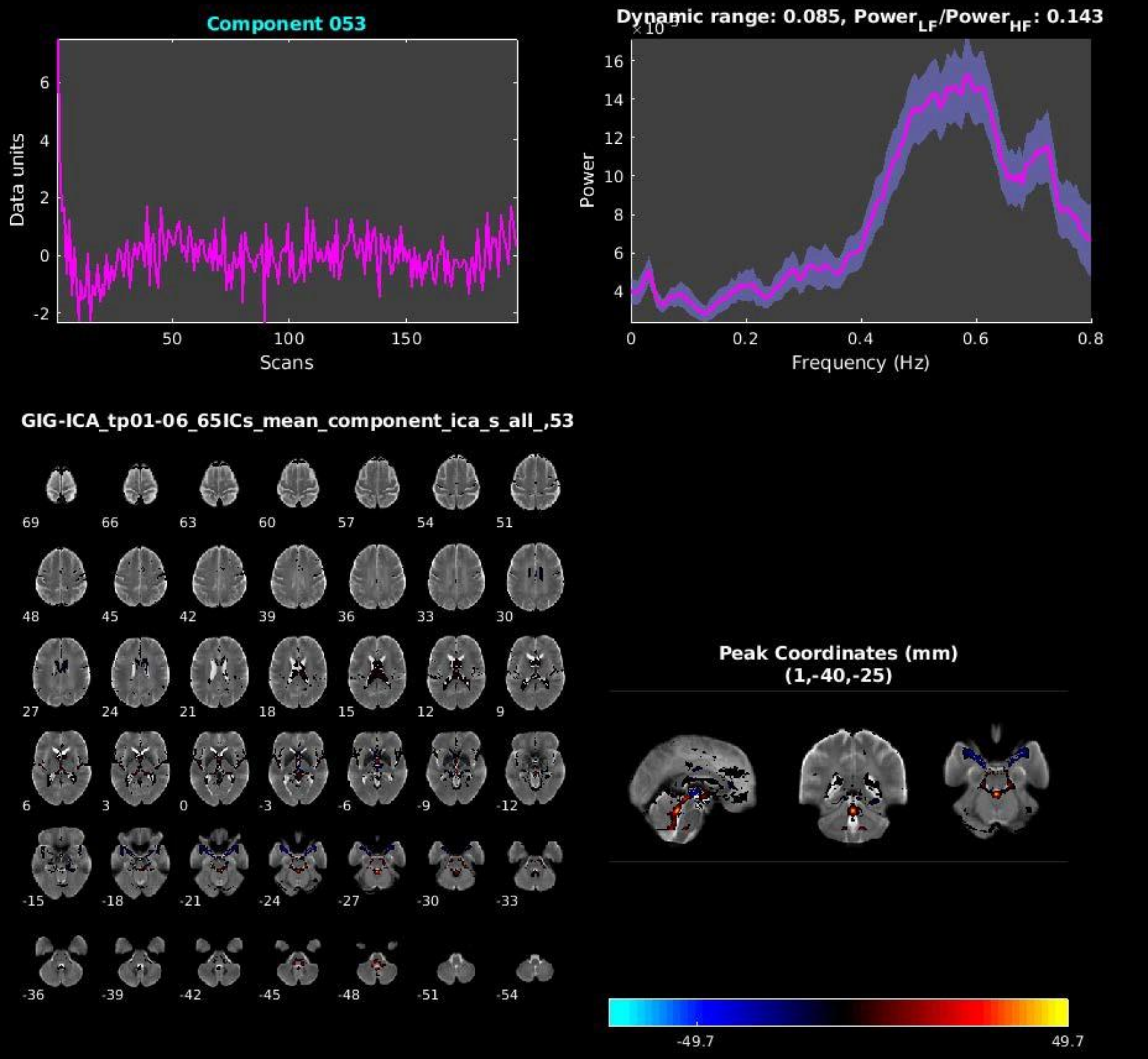
Component 053
Data units
Scans
Dynamic range: 0.085, Power_LF/Power_HF: 0.143
Power
Frequency (Hz)
GIG-ICA_tp01-06_65ICs_mean_component_ica_s_all_,53
Peak Coordinates (mm)
(1,-40,-25)
-49.7
49.7

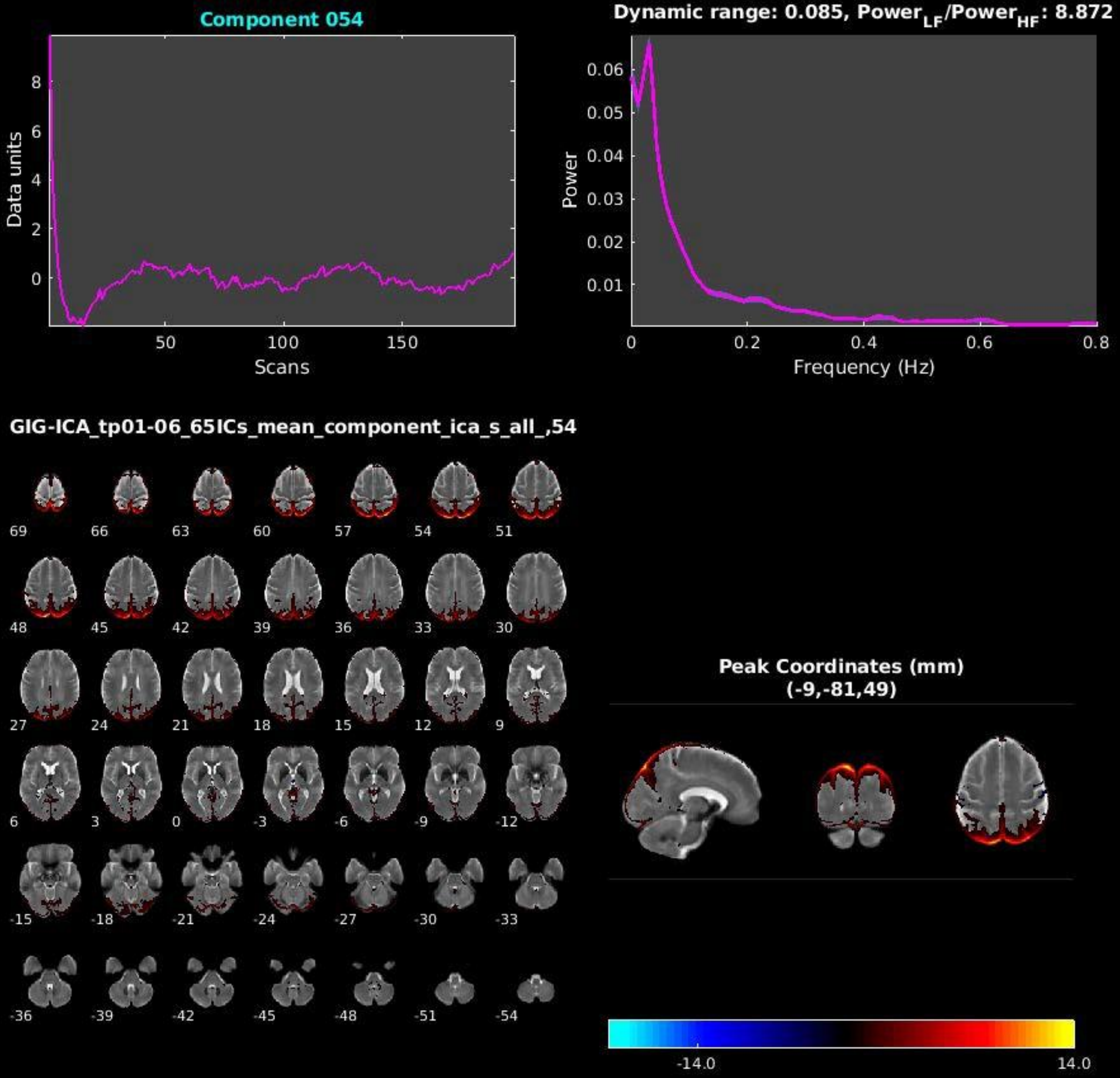
Component 054
Data units
Scans
Dynamic range: 0.085, Power$_{LF}$/Power$_{HF}$: 8.872
Power
Frequency (Hz)
GIG-ICA_tp01-06_65ICs_mean_component_ica_s_all_,54
Peak Coordinates (mm)
(-9,-81,49)
-14.0
14.0

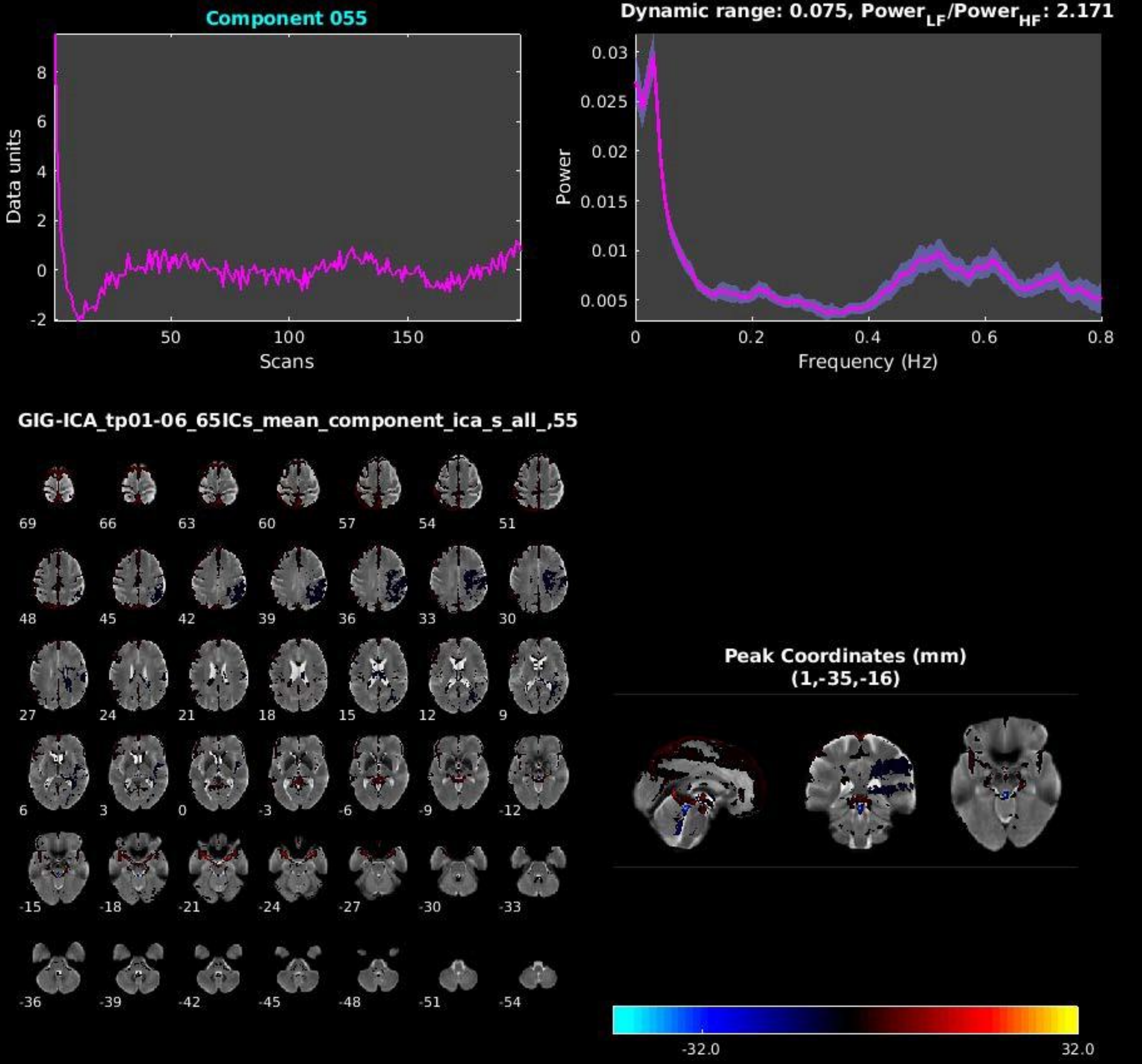
Component 055
Data units
Scans
Dynamic range: 0.075, Power$_{LF}$/Power$_{HF}$: 2.171
Power
Frequency (Hz)
GIG-ICA_tp01-06_65ICs_mean_component_ica_s_all_,55
Peak Coordinates (mm)
(1,-35,-16)
-32.0
32.0

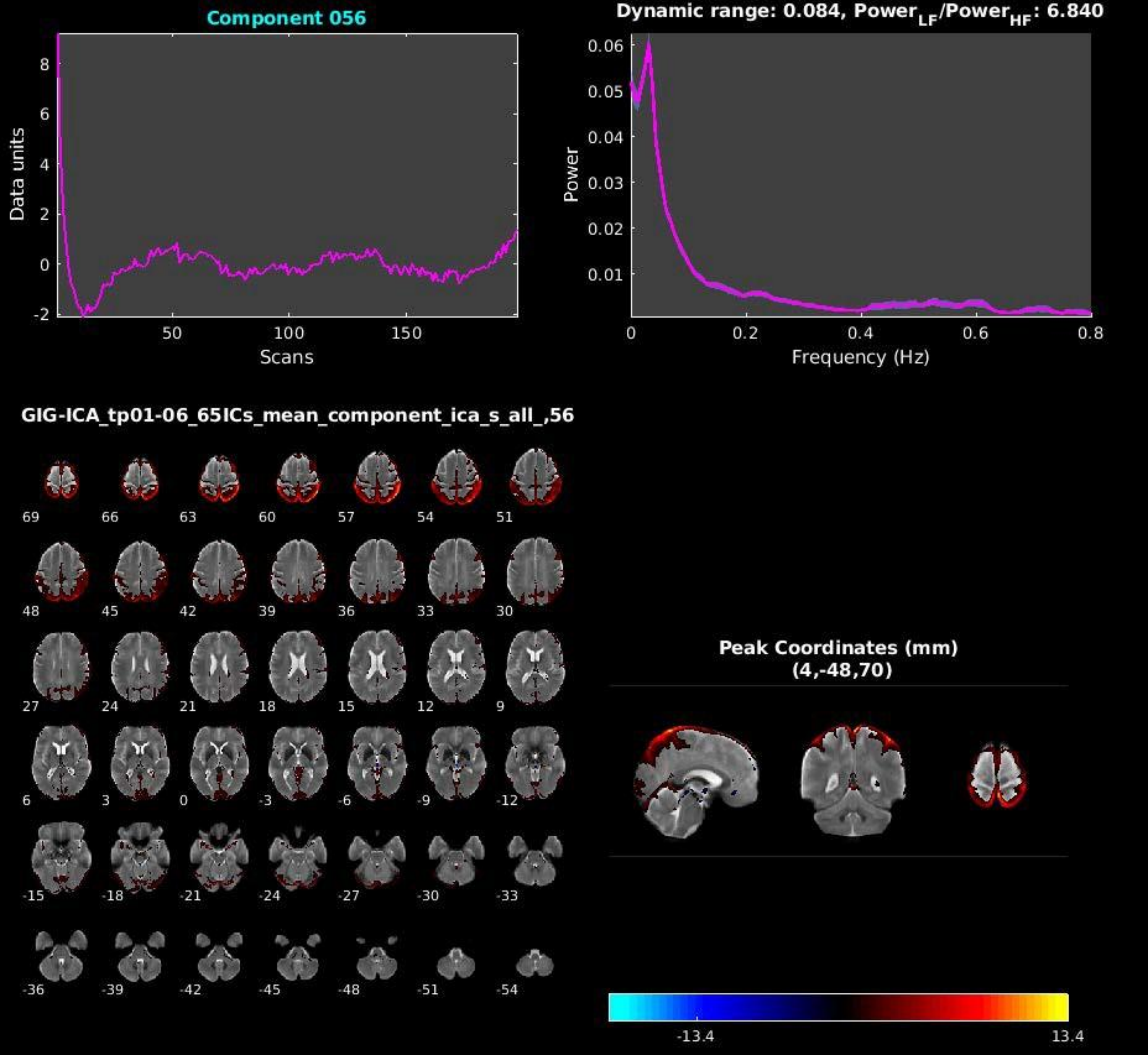
Component 056
Data units
Scans
Dynamic range: 0.084, Power_LF/Power_HF: 6.840
Power
Frequency (Hz)
GIG-ICA_tp01-06_65ICs_mean_component_ica_s_all_,56
Peak Coordinates (mm)
(4,-48,70)
-13.4
13.4

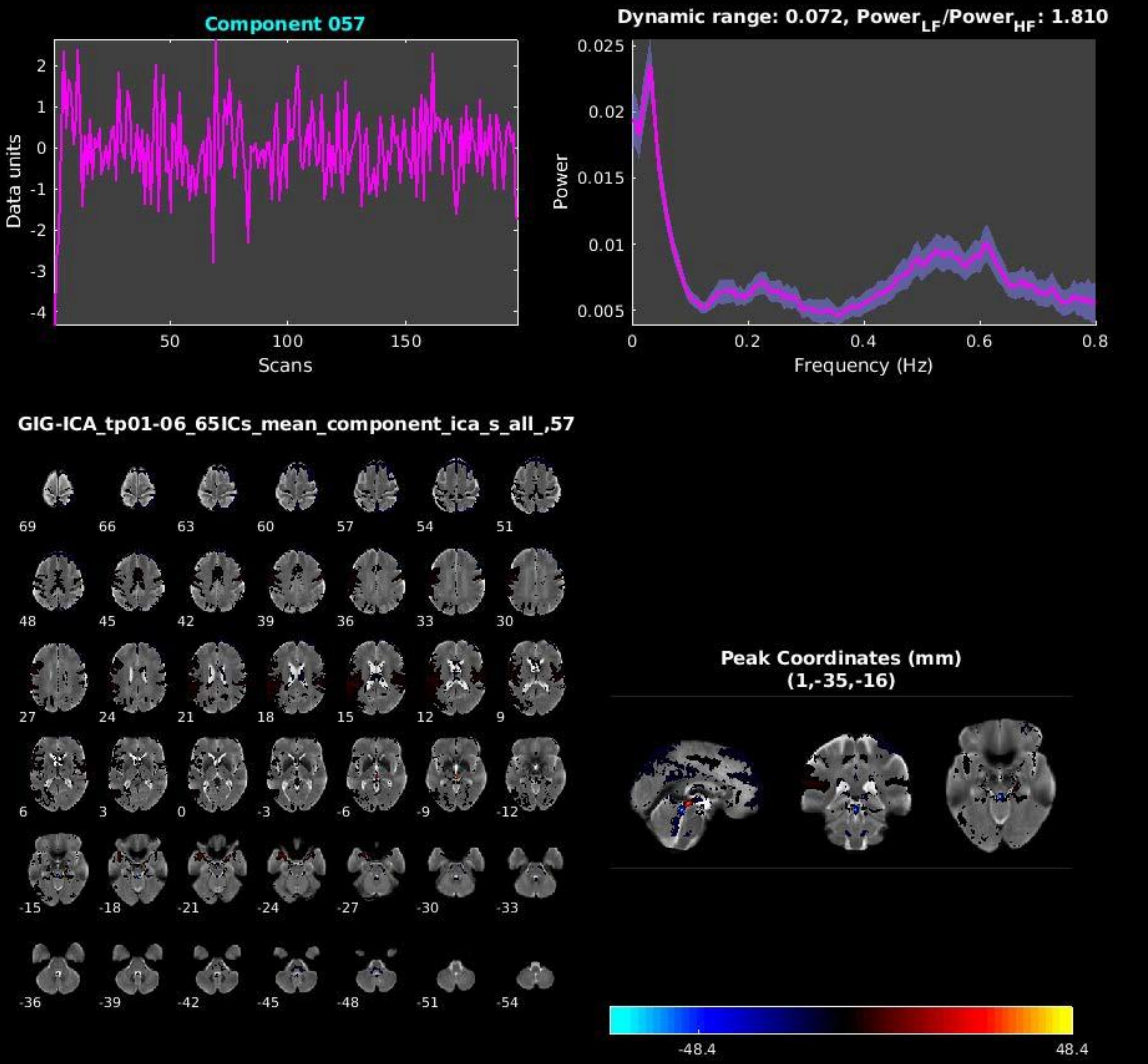
Component 057
Data units
Scans
Dynamic range: 0.072, Power$_{LF}$/Power$_{HF}$: 1.810
Power
Frequency (Hz)
GIG-ICA_tp01-06_65ICs_mean_component_ica_s_all_,57
Peak Coordinates (mm)
(1,-35,-16)
-48.4
48.4

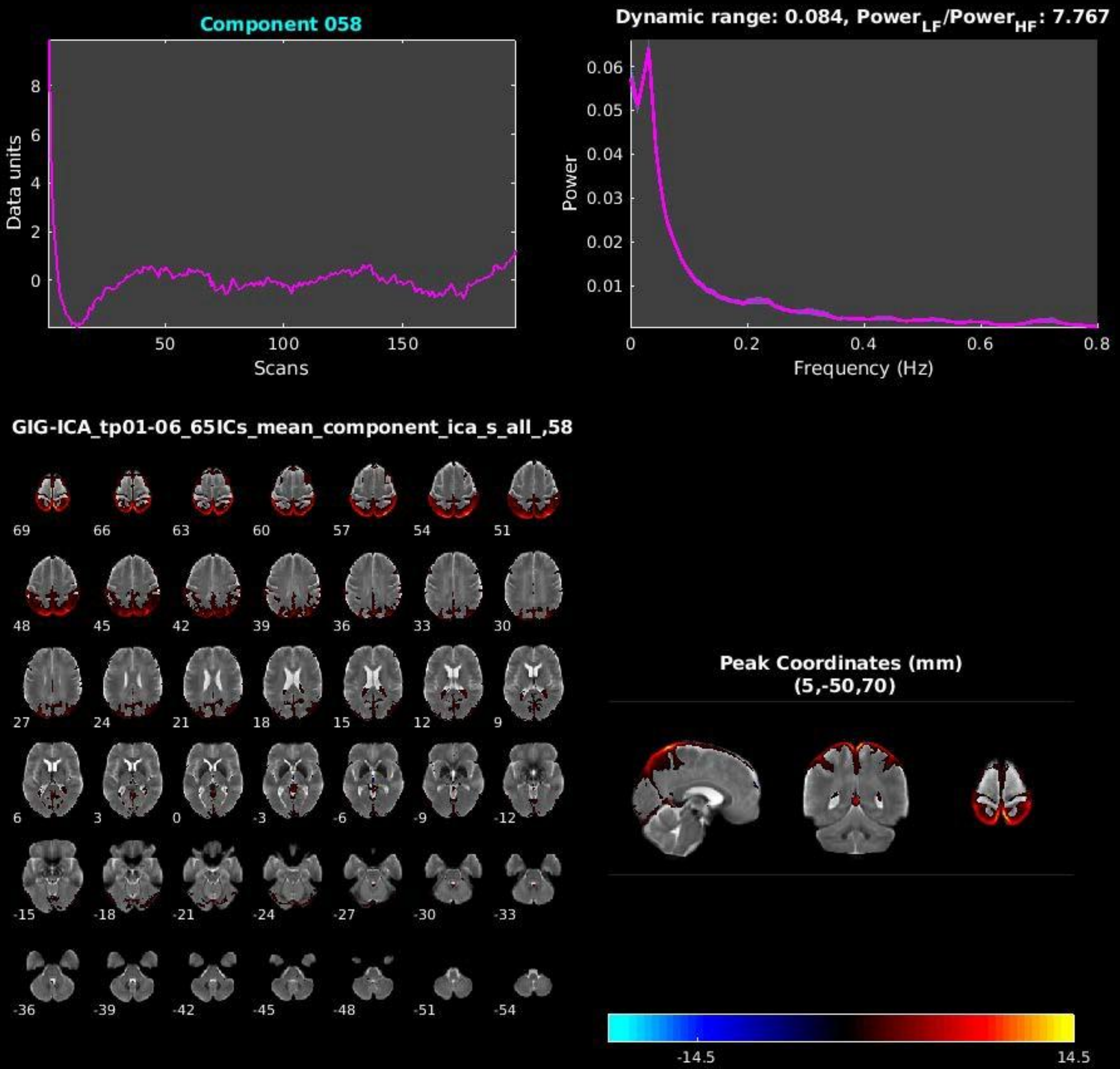

Component 058
Data units
Scans
Dynamic range: 0.084, Power_LF/Power_HF: 7.767
Power
Frequency (Hz)
GIG-ICA_tp01-06_65ICs_mean_component_ica_s_all_,58
Peak Coordinates (mm)
(5,-50,70)
-14.5
14.5

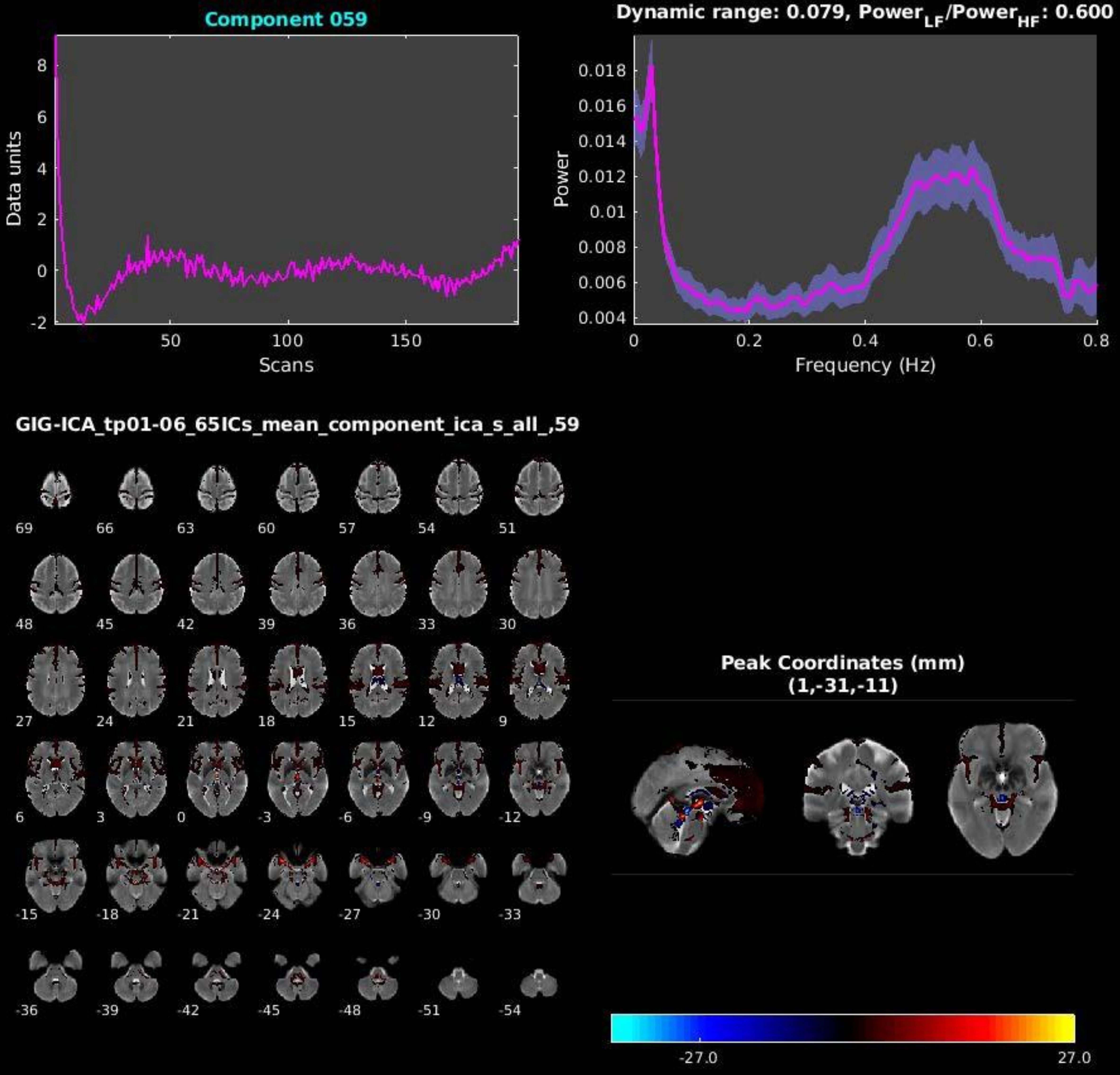
Component 059
Data units
Scans
Dynamic range: 0.079, Power$_{LF}$/Power$_{HF}$: 0.600
Power
Frequency (Hz)
GIG-ICA_tp01-06_65ICs_mean_component_ica_s_all_,59
Peak Coordinates (mm)
(1,-31,-11)
-27.0
27.0

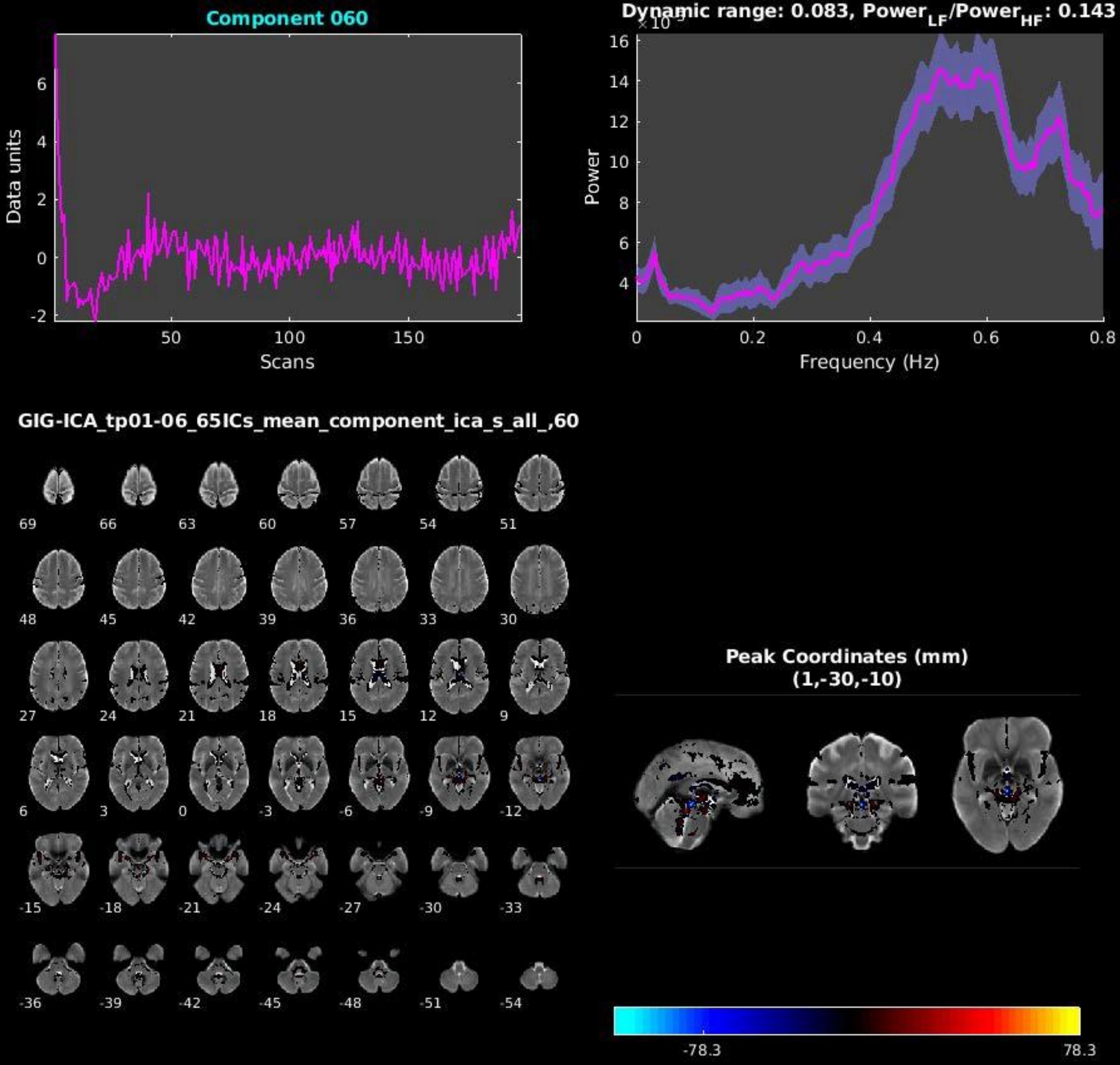
Component 060
Data units
Scans
Dynamic range: 0.083, Power$_{LF}$/Power$_{HF}$: 0.143
Power
Frequency (Hz)
GIG-ICA_tp01-06_65ICs_mean_component_ica_s_all_,60
Peak Coordinates (mm)
(1,-30,-10)
-78.3
78.3

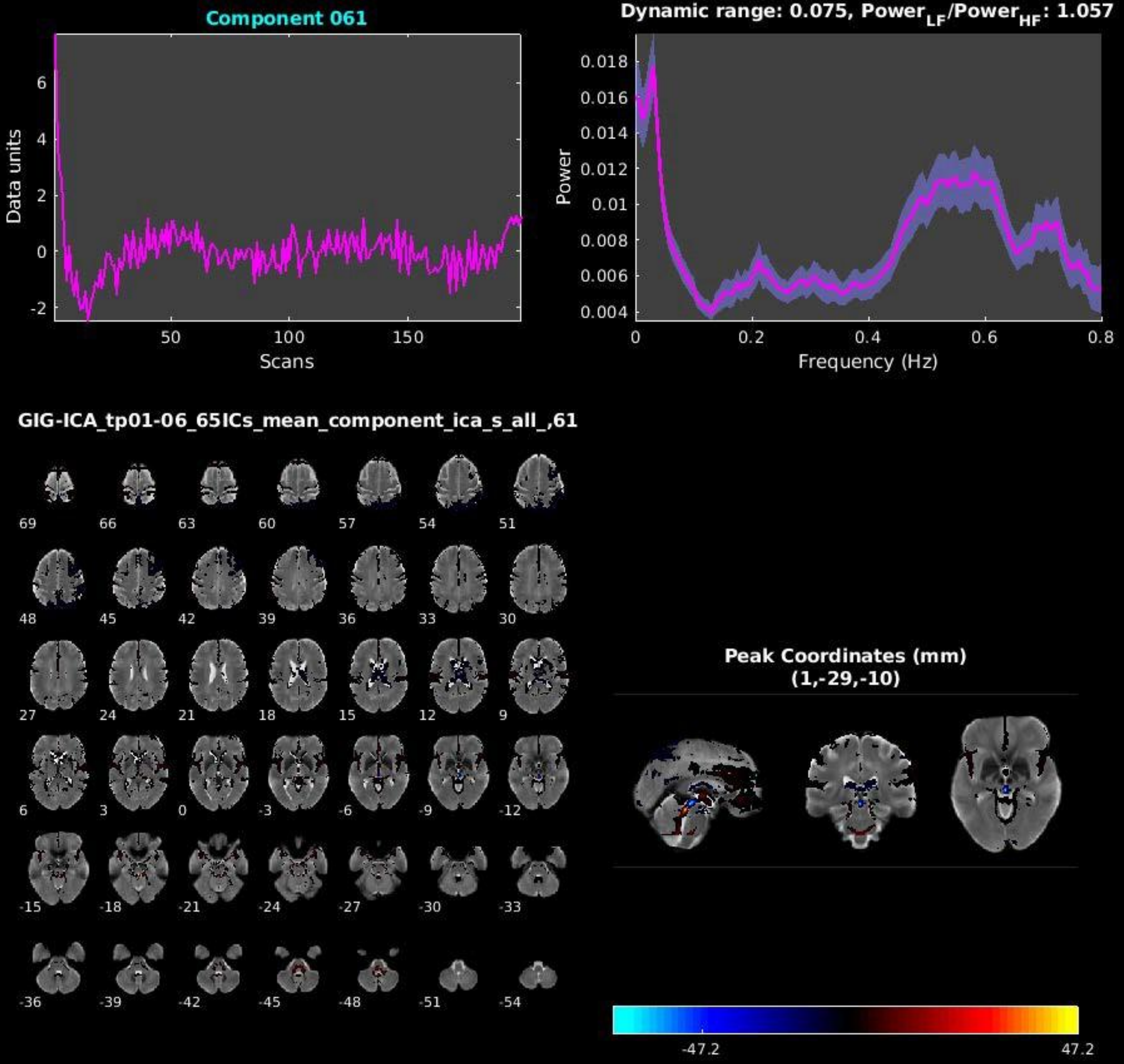
Component 061
Data units
Scans
Dynamic range: 0.075, Power_LF/Power_HF: 1.057
Power
Frequency (Hz)
GIG-ICA_tp01-06_65ICs_mean_component_ica_s_all_,61
Peak Coordinates (mm)
(1,-29,-10)
-47.2
47.2

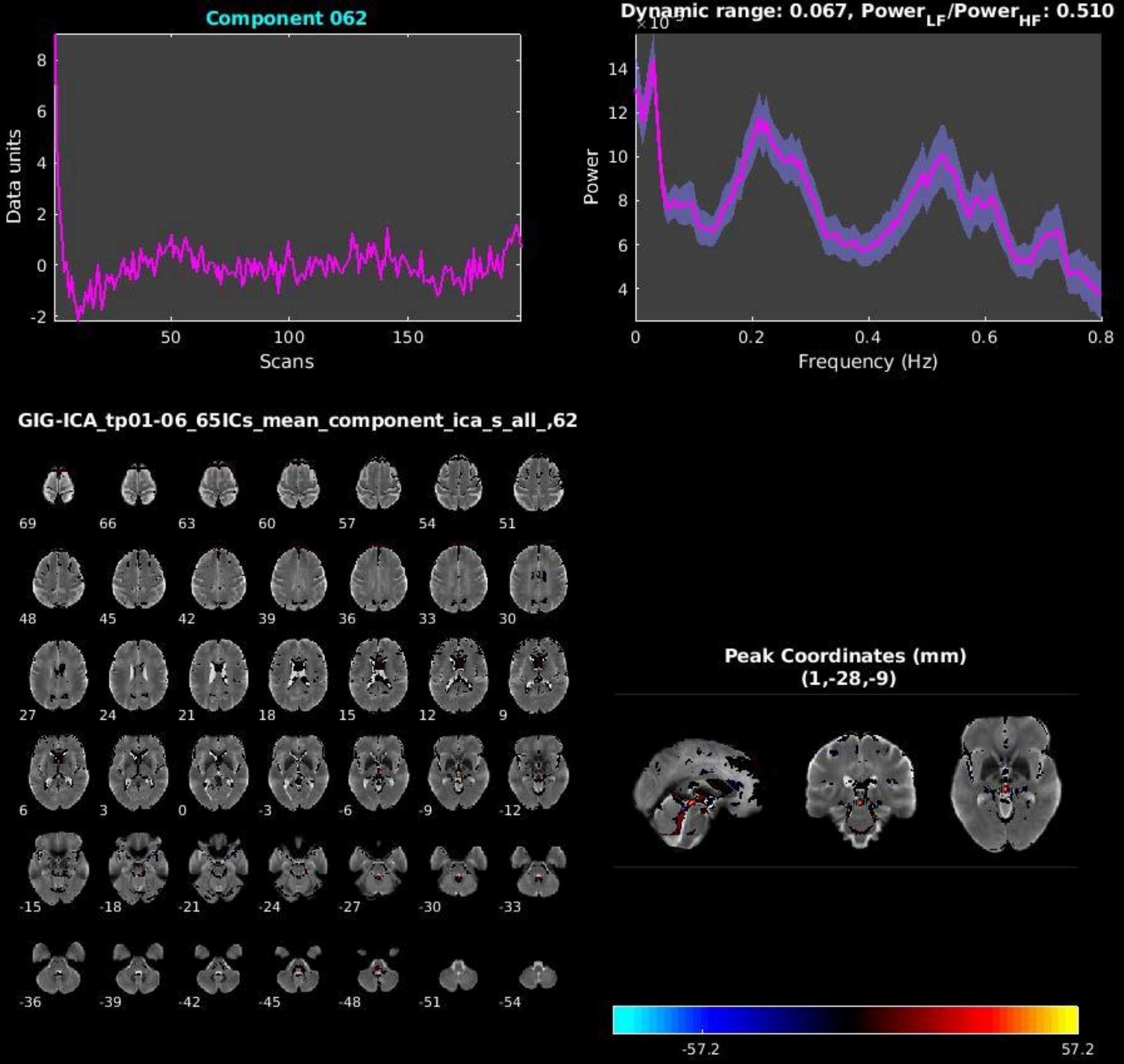
Component 062
Data units
Scans
Dynamic range: 0.067, Power_LF/Power_HF: 0.510
Power
Frequency (Hz)
GIG-ICA_tp01-06_65ICs_mean_component_ica_s_all_,62
69 66 63 60 57 54 51
48 45 42 39 36 33 30
27 24 21 18 15 12 9
6 3 0 -3 -6 -9 -12
-15 -18 -21 -24 -27 -30 -33
-36 -39 -42 -45 -48 -51 -54
Peak Coordinates (mm)
(1,-28,-9)
-57.2
57.2

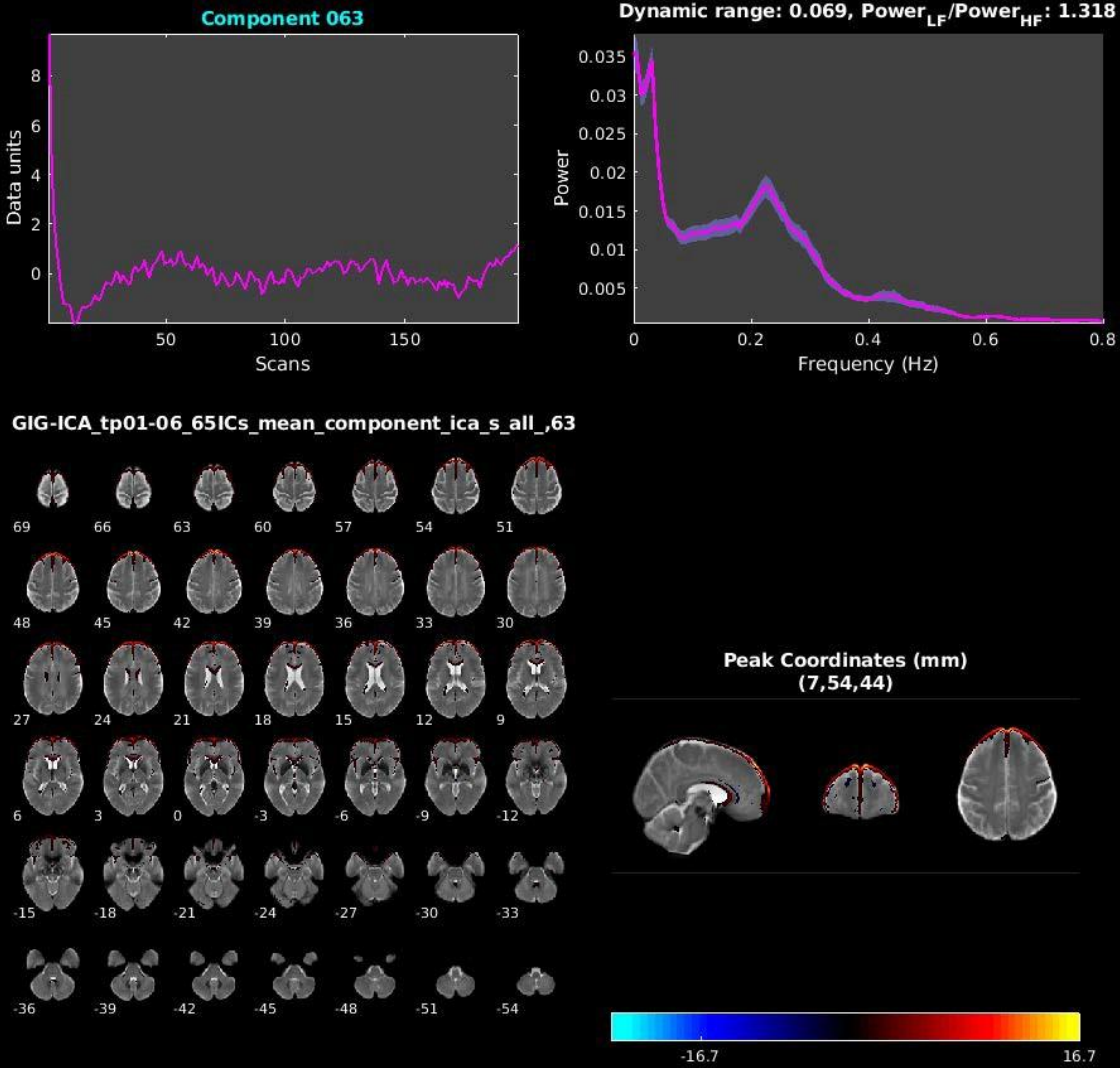

Component 063
Data units
Scans
0
2
4
6
8
50
100
150
Dynamic range: 0.069, Power$_{LF}$/Power$_{HF}$: 1.318
Power
Frequency (Hz)
0.035
0.03
0.025
0.02
0.015
0.01
0.005
0
0.2
0.4
0.6
0.8
GIG-ICA_tp01-06_65ICs_mean_component_ica_s_all_,63
69
66
63
60
57
54
51
48
45
42
39
36
33
30
27
24
21
18
15
12
9
6
3
0
-3
-6
-9
-12
-15
-18
-21
-24
-27
-30
-33
-36
-39
-42
-45
-48
-51
-54
Peak Coordinates (mm)
(7,54,44)
-16.7
16.7

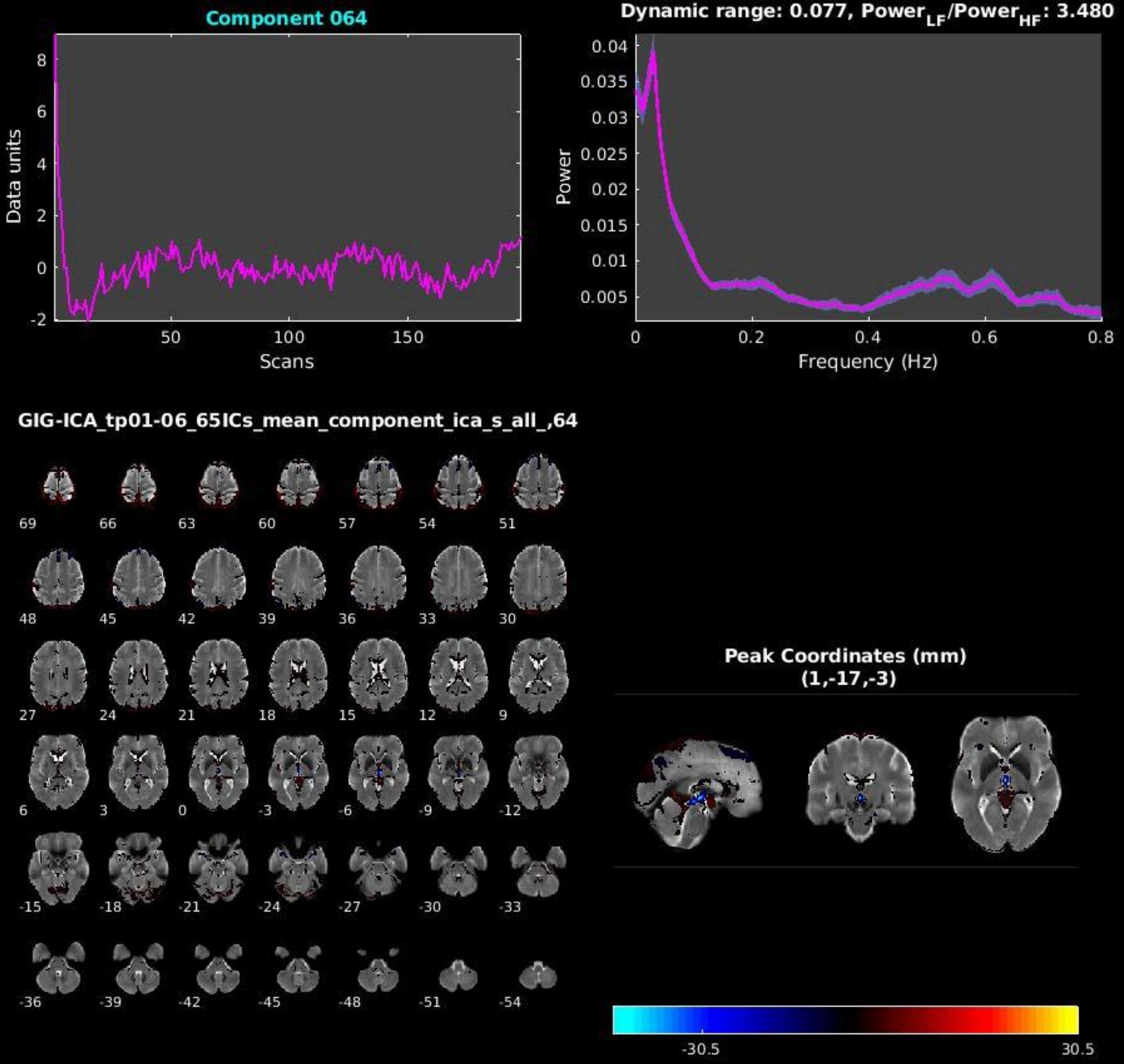

Component 064
Data units
Scans
Dynamic range: 0.077, Power_LF/Power_HF: 3.480
Power
Frequency (Hz)
GIG-ICA_tp01-06_65ICs_mean_component_ica_s_all_,64
69 66 63 60 57 54 51
48 45 42 39 36 33 30
27 24 21 18 15 12 9
6 3 0 -3 -6 -9 -12
-15 -18 -21 -24 -27 -30 -33
-36 -39 -42 -45 -48 -51 -54
Peak Coordinates (mm)
(1,-17,-3)
-30.5
30.5

SUPPLEMENTARY DATA

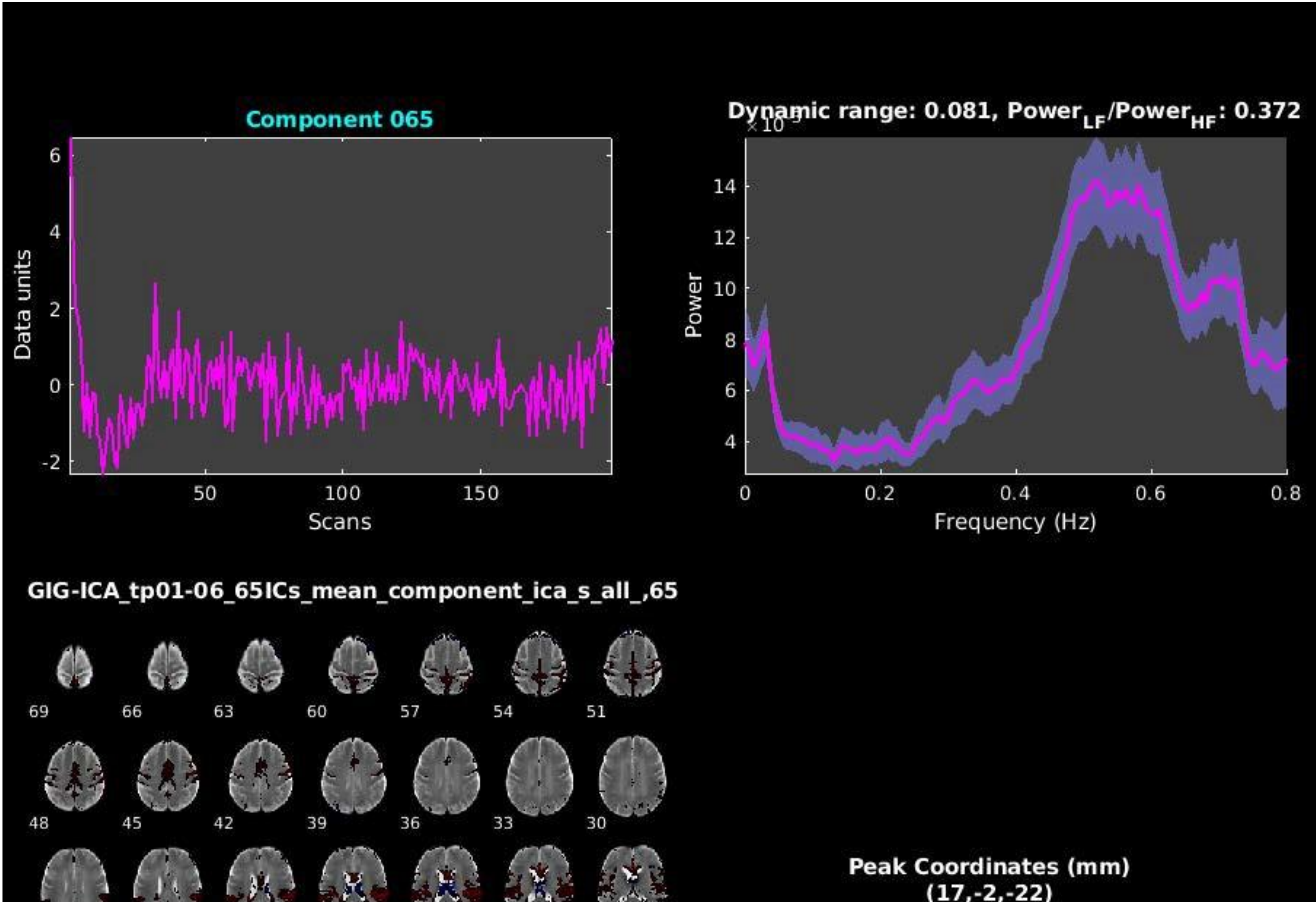

Supplement: Supplementary file 1 — The Supplementary data can be found online at: www.aginganddisease.org/EN/10.14336/AD.2024.0642. [file AD-16-3-1732-s.pdf]
